# Benchmarking food environment policies for the prevention of diet-related non-communicable diseases in Kenya: National expert panel’s assessment and priority recommendations

**DOI:** 10.1371/journal.pone.0236699

**Published:** 2020-08-06

**Authors:** Gershim Asiki, Milkah N. Wanjohi, Amy Barnes, Kristin Bash, Stella Muthuri, Dickson Amugsi, Danielle Doughman, Elizabeth Kimani, Stefanie Vandevijvere, Michelle Holdsworth

**Affiliations:** 1 African Population and Health Research Center, Nairobi, Kenya; 2 Department of Women’s and Children’s Health, Karolinska Institutet, Solna, Sweden; 3 Public Health, School of Health and Related Research (ScHARR), University of Sheffield, Sheffield, United Kingdom; 4 School of Population Health, The University of Auckland, Auckland, New Zealand; 5 French National Research Institute for Sustainable Development (IRD), NUTRIPASS Unit: IRD-Univ Montpellier-SupAgro, Montpellier, France; Cincinnati Children's, UNITED STATES

## Abstract

**Introduction:**

Unhealthy food environments drive the increase of diet-related non-communicable diseases (NCDs).

**Objective:**

We aimed to examine healthy food environment policies in Kenya and identify priorities for future action.

**Methods:**

Using the Healthy Food Environment Policy Index (Food-EPI) we collected evidence on the extent of government action to create healthy food environments across 13 policy and infrastructure support domains and 43 related good practice indicators between 2017 and 2018. A panel of 15 national experts rated the extent of government action on each indicator compared to the policy development cycle and international best practice respectively. Based on gaps found, actions to improve food environments in Kenya were identified and prioritized.

**Results:**

In the policy development cycle, 16/43 (37%) of good practice policy indicators were judged to be in ‘implementation’ phase, including: food composition targets, packaged foods’ ingredient lists/nutrient declarations; systems regulating health claims; restrictions on marketing breast milk substitutes; and school nutrition policies. Infrastructure support actions in ‘implementation’ phase included: food-based dietary guidelines; strong political support to reduce NCDs; comprehensive NCD action plan; transparency in developing food policies; and surveys monitoring nutritional status. Half (22/43) of the indicators were judged to be ‘in development’. Compared to international best practice, the Kenyan Government was judged to be performing relatively well (‘medium’ implementation) in one policy (restrictions on marketing breast milk substitutes) and three infrastructure support areas (political leadership; comprehensive implementation plan; and ensuring all food policies are sensitive to nutrition). Implementation for 36 (83.7%) indicators were rated as ‘low’ or ‘very little’. Taking into account importance and feasibility, seven actions within the areas of leadership, food composition, labelling, promotion, prices and health-in-all-policies were prioritized.

**Conclusion:**

This baseline assessment is important in creating awareness to address gaps in food environment policy. Regular monitoring using Food-EPI may contribute to addressing the burden of diet-related NCDs in Kenya.

## Introduction

An unhealthy diet is one of the major modifiable risk factors for non-communicable diseases (NCDs) in low- and middle-income countries (LMICs). NCDs account for an estimated 63% of mortality globally and 80% of the mortality occurs in LMICs [1, 2]. Diet-related risk factors have been increasingly shown to contribute to the burden of NCDs [3]. It has been established that unhealthy diets containing energy dense nutrient poor foods, high in fat, salt and added sugars now contribute to more NCDs than physical inactivity, alcohol and smoking combined [4]. Food environment—defined as the physical, economic, political and socio-cultural surroundings and conditions that influence what people eat—is the main driver of unhealthy diets [5]. Government policies that support healthy food environments are thus needed to address dietary risk factors for NCDs [5–7]. There is an expert consensus internationally on the policy actions needed by governments to create healthy food environments [5].

Many countries in sub-Saharan Africa (SSA) including Kenya are experiencing rapid urbanization, associated with increasing levels of overweight, obesity, and nutrition related NCDs with higher levels among urban residents, and women in particular [8, 9]. NCDs now account for 37% of all deaths in rural Kenya [10], while a fourfold increase in mortality due to NCDs was observed in urban Kenya between 2003 and 2012 [11]. The increase in the burden of NCDs in urban areas has been attributed to changing social and physical environments, food habits, and a proliferation of energy-dense nutrient poor foods and beverages entering the diet, often high in trans fats, salt, and sugar [12, 13]. Although the Kenyan national NCD prevention strategy recognizes NCDs as a pressing health concern and calls for improved policy formulation, including legislation and interventions, to promote healthy diets as a key strategy in the fight against NCDs, important gaps exist on the contextualization of available food policies and their evaluation in promoting healthy eating habits.

We recently conducted a multi-country assessment of NCD primary prevention policies for alcohol, tobacco, physical activity and nutrition in Kenya, Cameroon, South Africa, Malawi, and Nigeria and found that inadequate attention was paid by all the countries to address “best-buy” interventions for unhealthy diets [14]. In 2013, The International Network for Food and Obesity/NCDs Research, Monitoring and Action Support (INFORMAS) developed the Healthy Food Environment Policy Index (Food-EPI) tool and process, and recommended its use for assessing the level of implementation of policy actions by national-level governments compared to international best practice [15]. So far, the Food-EPI tool has been used in high-income countries and a few middle-income countries in Asia, Latin America, and recently in Africa [15–21]. We used the Food-EPI to examine healthy food environment policies in Kenya and identified priorities for future government action.

## Methods

**Fig 1:**
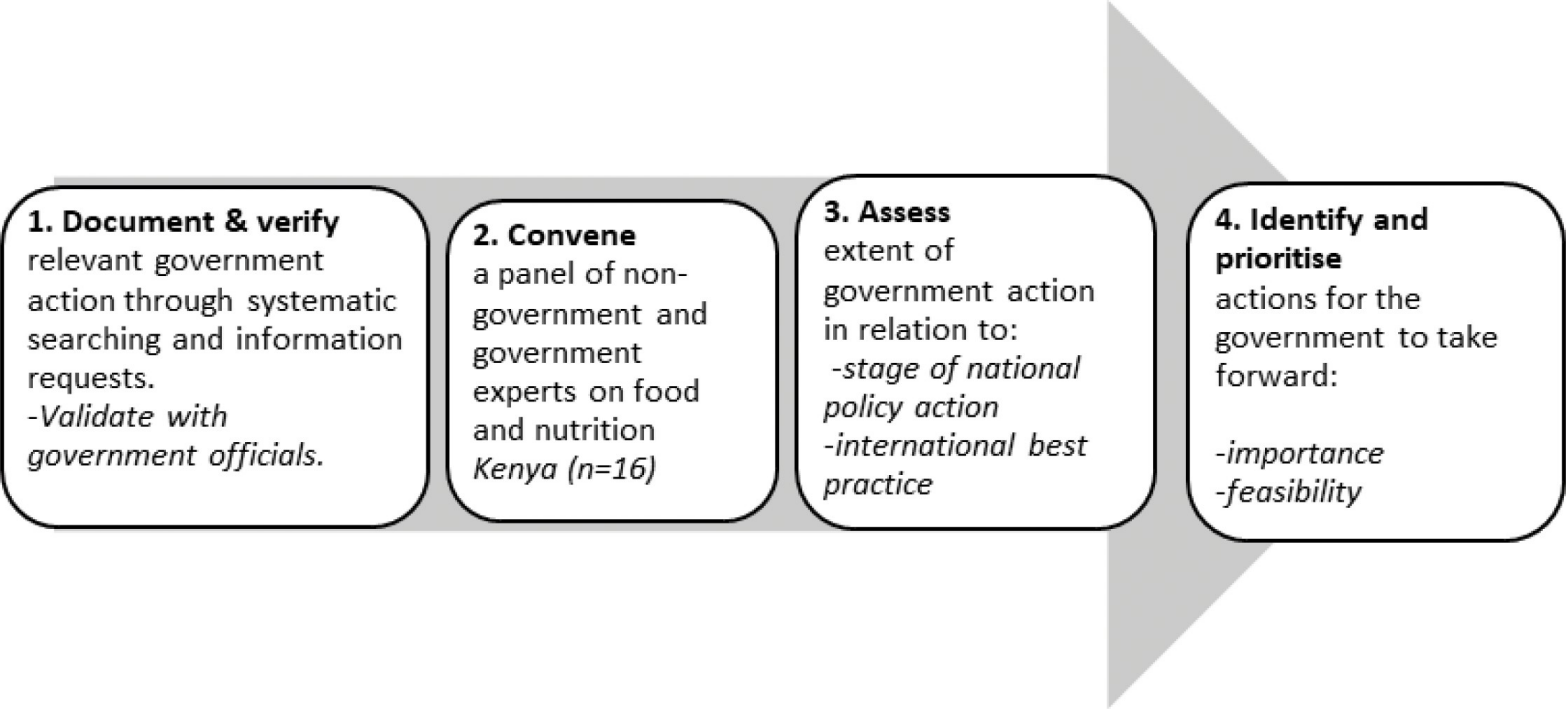
The food-EPI process in Kenya.

Between October 2017 and August 2018 a cross-country team of researchers undertook the Food-EPI process after training in the method from a Food-EPI expert (SV). The domains and indicators of good practice were tailored to the Kenyan context by the research team, in consultation with the INFORMAS team and according to the Food-EPI protocol [15] to assess Kenya’s policies for creating healthy food environments. The four steps taken in the Kenya Food-EPI processes are shown below (Fig 1).

### Stage 1: Document and verify

**Fig 2:**
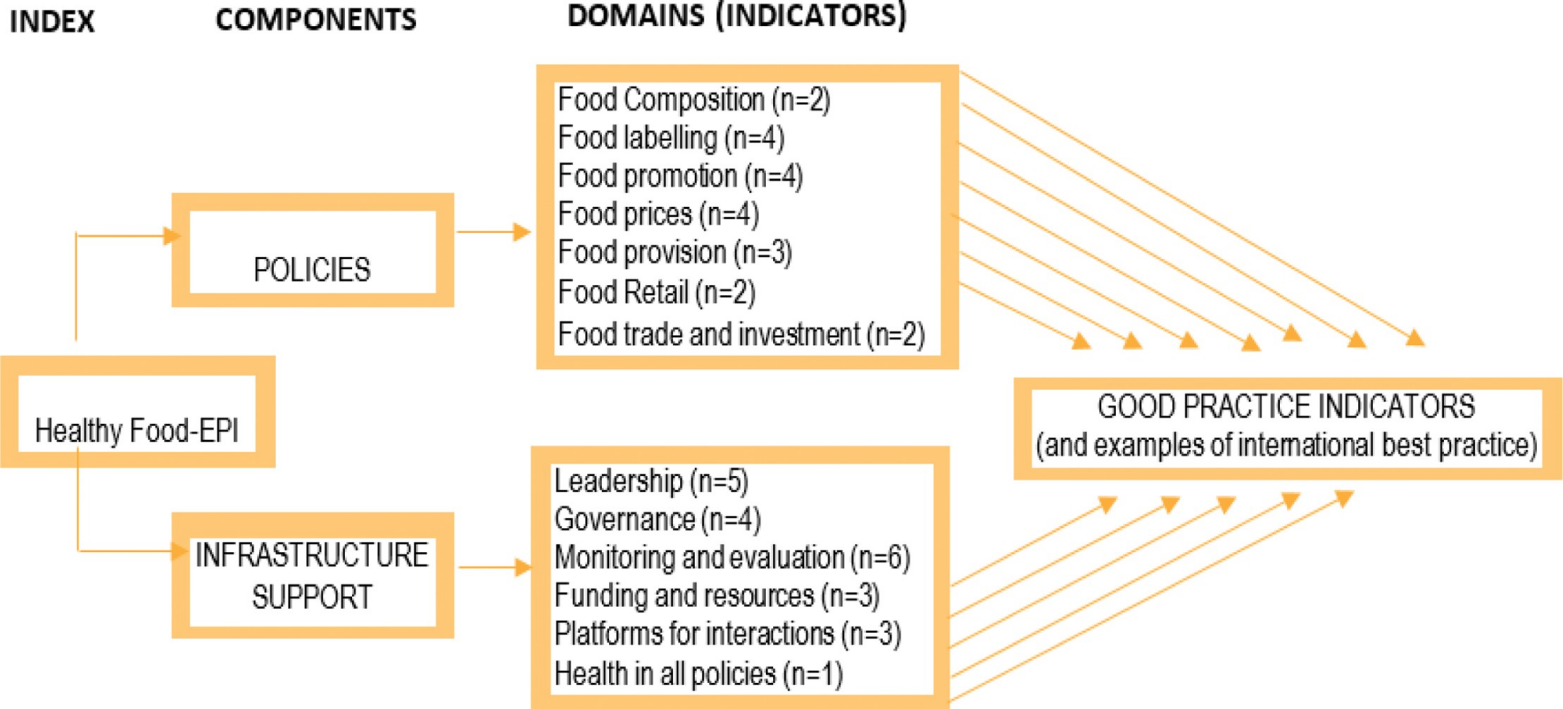
Components, domains and indicators of the adapted food-EPI tool used in Kenya.

This stage involved gathering evidence on the extent of government action to implement food environment policies across 13 policy and infrastructure support domains and 43 related sub-areas (indicators) of good practice (Fig 2). We searched government policies, work plans, and national strategies, as well as evidence of formal/informal activity across policy processes (from agenda-setting to implementation, monitoring and evaluation). We systematically searched government websites, websites of other institutions (e.g. FAO, WHO, UNICEF) and academic databases (for peer-reviewed articles) for evidence of action in addition to requests for information from relevant government authorities. Physical searches and reviews of documents not available online were also conducted. The research team compiled information on the extent to which policies existed in Kenya and were implemented across the 13 domains into a draft “evidence pack” (S1 Table).

The draft evidence pack was then shared with 21 key officials from units/divisions within government ministries such as Ministry of Health, Ministry of Education, Ministry of Trade, government parastatals (such as parastatals involved in food standards and regulation), the private sector (including food manufacturing companies) and non-government organizations, for feedback and validation. The officials were requested to review the evidence pack, validate the information summarised therein, and suggest any policies or government actions that had not been captured. This review and validation of the “evidence pack” by the stakeholders lasted for two months (April to May 2018). Feedback was received and incorporated into a final evidence pack in June and July 2018. In the evidence pack, information about action taken by the Government of Kenya to create healthier food environments was presented alongside examples of international best practice, as identified by INFORMAS.

### Stage 2: Convene

Invitation letters to attend a ratings workshop were sent with the evidence pack to a panel of experts on food and nutrition issues in Kenya from both non-governmental and governmental sectors. The evidence pack was shared with the experts two weeks before attending the rating workshop. The experts were asked to note down possible proposed actions for each of the Food EPI domains for discussion during the ratings workshop.

### Stage 3: Assess

**Fig 3:**
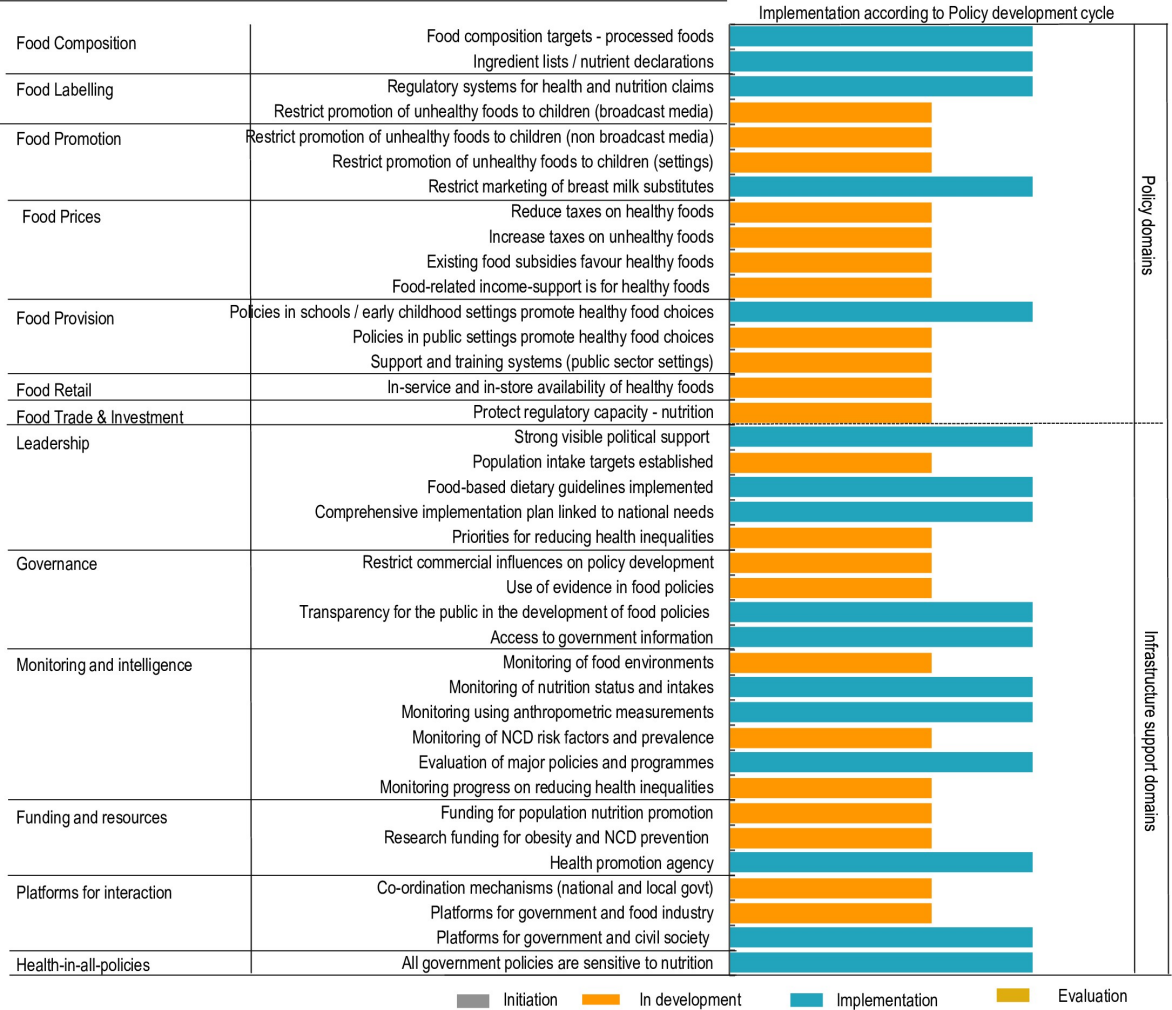
Implementation of food environment policy action and infrastructure support in relation to the policy development cycle.

**Fig 4:**
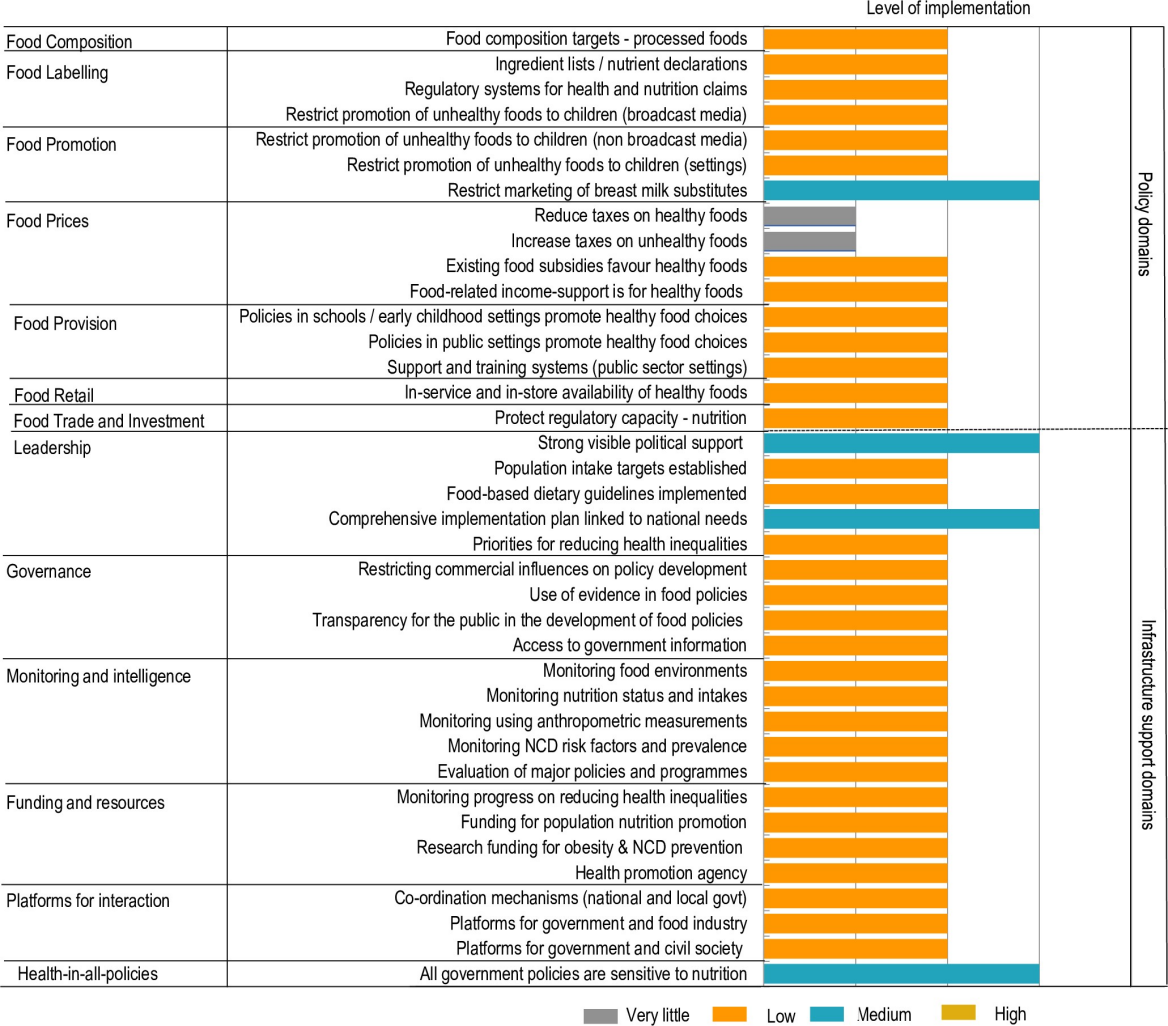
Implementation of food environment policy action and infrastructure support in relation to international best practice.

The expert panel ratings workshop was held on July 26, 2018 in Nairobi to rate the extent of government action to implement policies on food environments and infrastructure support against: (i) policy development cycle and (ii) international best practice. The ratings covered all 13 of the policy and infrastructure support domains and 43 indicators of good practice that are listed in Figs 2–4. Upon arrival, each expert was issued with a unique non-identifiable number and tablet computer for rating. Prior to the rating, experts provided demographic information relevant to their professional position, such as age, gender, type of employment and sector, and years of professional experience. The research team then presented a summary of the evidence of implementation for each indicator. This allowed clarification from the experts on the processes, followed by discussion to facilitate their understanding and judgement of the rating exercise. Experts first assessed implementation of each policy or government activity in relation to how far along the policy development cycle it was (*i*. *Initiation stage*, *ii*. *Policy development*, *iii*. *Implementation and iv*. *Evaluation phase*); rating Kenya’s activity to one of these stages of policy development. The experts were then asked to rate according to their perceived level of implementation of Kenya’s policies in relation to international best practices; judging each Food EPI indicator on a five-point Likert scale: (1) <20% implementation OR Very Low to No progress; (2)-20–40% implementation OR Low to Medium progress; (3) 40–60% implementation OR Good progress; (4) 60–80% implementation OR Very Good progress and (5) 80–100% implementation OR Excellent progress in relation to international best practices. If an expert did not feel confident in their ability to rate, they chose option (6)—*cannot rate*. In some cases, the experts engaged the research team on the evidence presented, for example sharing new and relevant evidence that had been missed in the evidence pack. Any new evidence suggested was documented by one of the team members but not considered during the rating exercise, since further review/verification was needed before inclusion in a revised evidence pack.

### Stage 4: Identify and prioritize

At the end of the workshop, the expert panel identified potential policy and infrastructure support actions that could be implemented by the government in Kenya. Those proposed actions for which there was a consensus were listed for ranking terms of their *importance* (the extent of significance of the anticipated value of the action), and then *feasibility* (how easily the action might be accomplished given political, budgetary and social realities). Since 23 actions had been proposed for both policies and infrastructure support, the ranking was undertaken on a scale of 1 to 23, with 1 as the most important or most feasible action and 23 as least important or least feasible action. This prioritization exercise was conducted independently by each participant using an electronic platform (survey CTO software) on individual handheld computers.

### Statistical analysis

Statistical analysis was conducted using Excel. Professional characteristics of the experts were summarized as count and percentages. Descriptive statistics (average and percentage) were computed to examine (i) ratings on how far along the policy development cycle, policies and government infrastructure support have been developed, (ii) ratings on the level of implementation of each policy and infrastructure support indicator against international benchmarks (ii) prioritization scores for importance and feasibility of policy actions and infrastructure support actions.

**Fig 5:**
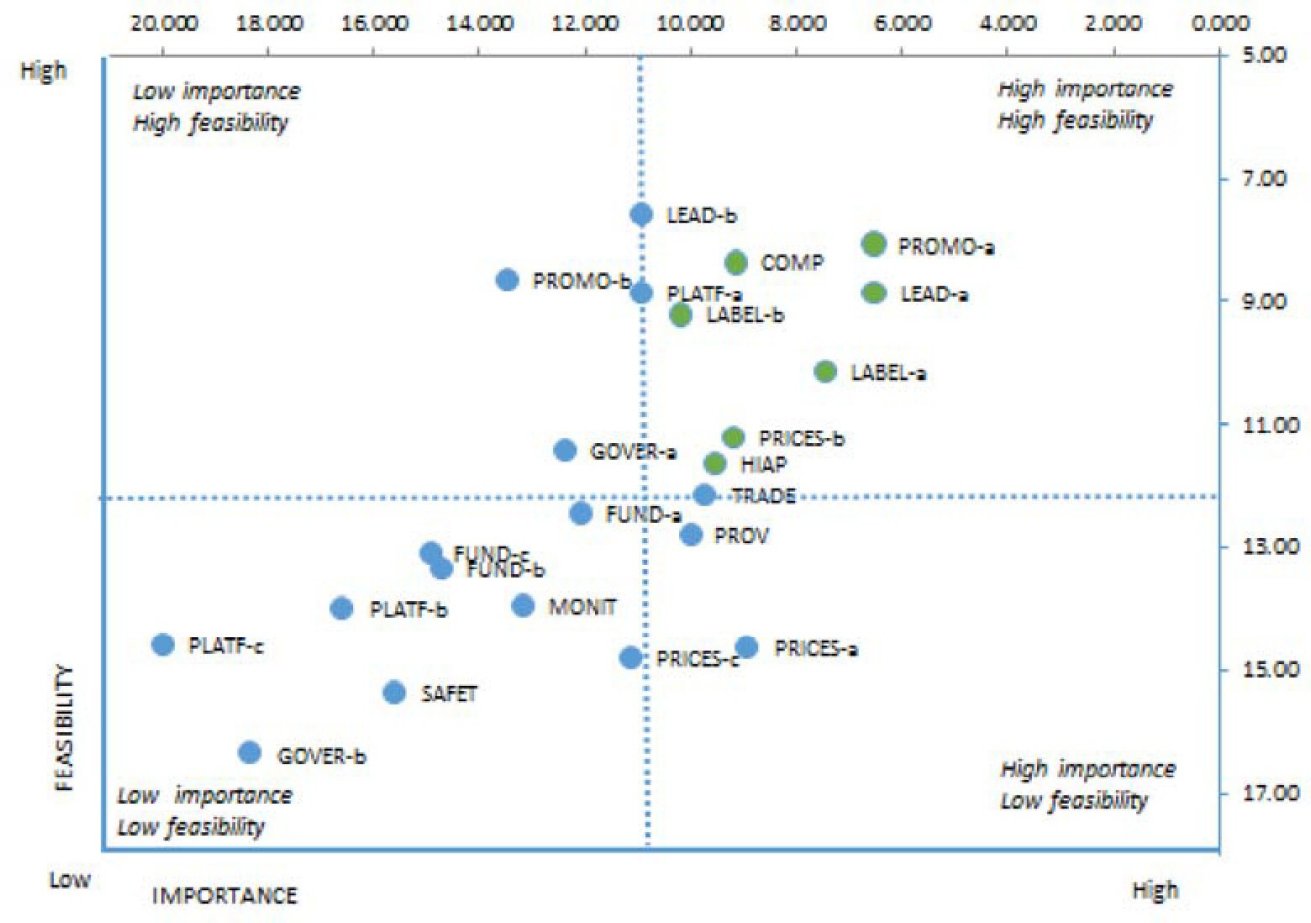
Final list of recommended actions for government in terms of importance and feasibility.

The mean rating for each indicator in the policy development cycle was subsequently categorized into four levels: “initiation” (<25%), “development” (26–50%), “implementation” (51–75%) and “evaluation” (>75%). For the level of implementation of policies and government infrastructure support in comparison to international best practice, ratings were also grouped into four categories: “very little, if any” (<25%), “low” (26–50%), “medium” (51–75%) and “high”‘ (>75%). Inter-rater reliability (Gwet AC2 coefficient) was calculated using Agreestat 2013.1, an Advanced Analytics, Gaithersburg, USA. In order to examine the differences in the ratings by professional characteristics of experts, we tested the distribution of the data using the Shapiro–Wilk test. As data were found to be non-normally distributed, we used Mann-Whitney U test to compare ratings based on experts’ professional background (government-employed versus NGO-employed), gender, and years of experience. Statistical significance was set as p value threshold of <0·05 for all analyses. For prioritization of proposed actions, the weights that the experts allocated to importance and feasibility were applied to their individual scores and the scores for importance and feasibility were then summed for each proposed action. Actions were ranked from higher to lower priority. Average points on *importance* and *feasibility* scales were mapped using a four-quadrant scatter graph using the INFORMAS protocol criteria. The actions were divided into four groups: (i) “relatively higher importance and relatively higher feasibility” group; (ii) “relatively higher importance and relatively lower feasibility group; (iii) “relatively lower importance and relatively higher feasibility” group; and (iv) “relatively lower importance and relatively lower feasibility” group. The points dividing the graph into the quadrants were calculated by summing the average score of all actions in each criterion and then dividing this sum by the total number of actions. The higher the points allocated to these two indicators the more likely the proposed policy actions to be assigned at the upper-right quadrant of the scatter graph (Fig 5).

### Ethics

The study received ethical approval from the AMREF Health Africa Ethics and Scientific Review Committee (ESRC P365/2017). All experts invited for the ratings workshop provided written informed consent before participating.

## Results

### Evidence included in the evidence pack

In stage 1, a total of 31 relevant policy documents were identified as providing relevant information on government action to create more healthy food environments in Kenya (Table 1). Most documents, except the Food, Drugs and Chemical Substance Act of 1978, were published or developed during 2003–2018. Five documents (National School Health Policy, the President’s Big Four Agenda, National Health Insurance Fund, East African Standards (2015), Nutrition Labelling—Requirements [KS EAS 803:2014]) were in draft stage. We included legislative documents to review government actions that might not have been included in the policy documents. Five documents were excluded because they were either outdated and replaced by newer versions or not relevant to food policies: Kenya National Diabetes Strategy-2010, Kenya Food Security and Nutrition Strategy-2008, Kenya Food Security Bill-2014, Kenyan Human Resources Strategy-2014, and Kenya National Plan of Action for Nutrition-1994. The evidence included in steps taken by government to create healthier food environments such as the National Nutrition Action Plan, School Nutrition and Meals Strategy for Kenya, Kenya National NCD strategy, National Guideline for Healthy Diets & Physical Activity and Kenya Health Sector Strategic and Investment Plan.

**Table 1:** Policy documents identified and included for review.

	INCLUDED		
	Name of Document	Date Published	Evidence Type
1	Kenya Food and Nutrition Security Policy	2011	Policy
2	Food Drugs and Chemical Substance Act	1978	Legislation
3	East African Standards—DRAFT	2015	Policy/strategy/guideline
4	Code of Advertising Practice and Direct Marketing	2003	Policy/strategy/guideline
5	National School Health Policy—DRAFT	DRAFT	Policy/strategy/guideline
6	Breast Milk Substitutes Act (Act No.34 of 2012)	2012	Legislation
7	Budget Statement for the Fiscal Year 2017–2018	2017	Budgetary document
8	Excise Duty Act	2015	Legislation
9	National Nutrition Action Plan	2012	Policy/strategy/guideline
10	School Nutrition and Meals Strategy for Kenya	2016	Policy/strategy/guideline
11	Kenya NCD National Strategy	2015	Policy/strategy/guideline
12	National Guideline for Healthy Diets & Physical Activity	2017	Policy/strategy/guideline
13	Kenya Health Bill	2016	Legislation
14	Kenya Health Sector Strategic and Investment Plan	2013	Policy/strategy/guideline
15	Mid-Term Review of the Kenya Health Sector Strategic Plan	2014	Report
16	Breastfeeding Mothers Bill	2007	Legislation
17	Access to information Bill	2013	Legislation
18	Public Participation Bill	2018	Legislation
19	Kenya Health Policy 2014–2030	2014	Policy/strategy/guideline
20	Constitution of Kenya	2010	Legislation
21	Kenya Vision 2030	2008	Policy/strategy/guideline
22	Kenya National Cancer Control Strategy	2017	Policy/strategy/guideline
23	Health in All Policies: Actions in the African Region	2013	Report
24	Nutrition Labelling—Requirements [KS EAS 803:2014]-DRAFT	2014	Policy/strategy/guideline
25	Use of Nutrition and Health Claims—Requirements	2014	Policy/strategy/guideline
26	Labeling of Prepackaged Foods—Specification	2014	Policy/strategy/guideline
27	Food Drugs and Chemical Substance Act	2015	Legislation
28	Big Four Agenda-DRAFT		Policy/strategy/guideline
29	National Research Fund	2015	Policy/strategy/guideline
30	National Science and Technology and Innovation Act	2013	Legislation
31	National Health Insurance Fund-DRAFT	-	Policy/strategy/guideline

### Characteristics of experts

In total, 42 experts were invited (stage 3) to assess and rate government action in Kenya: 16 attended the rating workshop and 15 scored the indicators because one expert arrived too late to participate in the rating. The majority of those who participated in the rating exercise were aged 35–50 years, and had worked for an average period of 11 years. Seven experts were from government, 5 were from non-government organizations, and 2 from parastatals. Men (47%) and women (53%) were represented nearly equally. Twenty-six experts who were invited did not attend the rating workshop. However, every area of expertise (apart from trade) was represented among those who attended (Table 2). Other participants had expertise in nutrition and dietetics, clinical nutrition, and nutrition laboratory services.

**Table 2:** Characteristics of experts who were invited for the policy rating workshop.

Characteristics	Participated		Not participated	
Employed	Number	%	Number	%
Government	8	53.3	13	50
Non-Government	7	46.7	13	50
**Expertise**	** **	** **		
Clinical nutrition	2	13.3	1	3.8
Advocacy	2	13.3	2	7.7
Standards	1	6.7	2	7.7
Trade			2	7.7
Medical specialist	1	6.7	3	11.5
Academia	3	20.0	4	15.4
Nutrition and dietetics	2	13.3	4	15.4
Public health nutrition	1	6.7	8	30.8
Gender and social services	1	6.7		
Nutrition laboratory	1	6.7		
Economics	1	6.7		
**Age**				
<35	4	26.7		
35–50	7	46.7		
>50 years	4	26.7		
**Gender**				
Men	7	46.7		
Women	8	53.3		
**Total**	**15**	**100.0**	**26**	**100.0**

*The expert who attended but did not rate policies was a public health nutrition expert from an NGO (not included in the table).

### Ratings for food environment policy action and infrastructure support against the policy development cycle

The inter-rater reliability of ratings performed by experts for the policy development cycle was 0.73 (95% CI; 0.59–0.86). As shown in Fig 3, government policy action for approximately one-third of the indicators (16/43) was rated in the implementation phase (5 policy, and 11 infrastructure support actions) including: food composition targets, packaged foods’ ingredient lists/nutrient declarations; systems regulating health claims; restrictions on marketing breast milk substitutes; and school nutrition policies. Among infrastructure support actions in the implementation phase were: food-based dietary guidelines; strong political support to reduce NCDs; comprehensive NCD action plan; transparency in developing food policies; and surveys monitoring nutritional status. The existence of a health promotion agency, platform for government and civil society and ensuring that all government policies are sensitive to nutrition were among those considered to be in the implementation phase. There were 22/43 areas of government policy action that were judged to be only “in development”, including action to restrict the promotion of unhealthy foods to children through the media. All indicators for food prices, food retail, and food trade/investment were rated in the development phase. For food provision, policies to promote healthy food in public settings, support and training systems in public sector settings were also judged to be in development stage. In terms of infrastructure support, indicators captured in Fig 3 were judged as in development. No indicators were judged as at the evaluation or initiation phase. No evidence of any government action was documented for five policy areas of good practice, relating to establishing food composition standards/targets for out-of-home meals in food service outlets; front-of-pack or menu board labelling systems; risk assessments for trade agreements; or zoning laws on the density/location of healthy/unhealthy food service outlets. (These are not shown in the scorecard).

### Ratings for extent of policy implementation against international best practice

The inter-rater reliability of ratings performed by experts for extent of policy implementation compared with international best practice was 0.55 (95% CI; 0.45–0.65). The Government of Kenya was assessed to be performing relatively well (medium implementation) in only four areas of good practice namely, restricting the marketing of breast milk substitutes, demonstrating political leadership, having a comprehensive implementation plan linked to national needs and ensuring all policies are sensitive to nutrition. Only one of these was from the food policy domain. Thirty-six indicators were rated as low or very little implementation, including recent government policy action on food composition, food labelling, food provision, food retail and food trade and investment. Infrastructure support actions relating to governance, monitoring and intelligence, funding and resources and platforms for interaction were also rated as low. Among the food prices indicators, reduction of taxes on healthy foods and increasing taxes on unhealthy foods were rated as very little in implementation at the level of international best practice (Fig 4).

### Comparison of ratings by demographic characteristics of experts

Ratings of scores for the policy development stage across all domains varied by characteristics of the experts. Women rated higher than men (mean score 2.5 vs 1.9; Mann–Whitney U test, p < 0.001), government experts rated higher than non-government actors (2.3 vs 2.0; p = 0.026), those who had worked for less than10 years had higher scores than those who had worked for 10 years or more (2.4 vs 2.1; p = 0.016) and those aged less than 35 years had higher scores than those older (2.6 vs 2.1; p < 0.01). Comparing the ratings of policies by experts against international benchmarks revealed a statistically significant difference between the ratings across all domains; men and women (1.7 vs 2.0; p = 0.034), experts who worked for less than 10 years compared to those who worked 10 or more years (2.1 vs 1.7; p = 0.003) and experts younger than 35 years compared to those 35 years or older (2.2 vs 1.7; p <0.001). There was no statistical difference in rating for government and non-government actors (1.9 vs 1.8; p = 0.187).

### Priority recommendations for government action

The expert panel identified 23 potential policy and infrastructure support actions that could be implemented by the government in Kenya. The top 7 actions, rated by experts in terms of importance and feasibility, are shown in the right upper quadrant of the scatter graph (Fig 5) and listed in Table 3. These mainly focused on ensuring the agro-food system is healthier, more sustainable and financially accessible and covered policy and infrastructure support actions. In terms of policy actions, the top 4 priorities of high importance and feasibility identified by the expert panel were:

- Legislation for nutrition standards for processed food industry;
- Elimination of trans fats from edible fats and vegetable oils and label them, if present in processed foods. Fried foods such as donuts, potato chips, bread and cakes in Kenya contain such trans fats.
- Introduction of nutrition labelling for processed foods and particularly in relation to trans fats consumption, and a policy that defines some levels of fats in foods to be labelled with “traffic lights” warning system following guidelines from FAO/WHO and other international best practices; and
- Subsidies to lower the price of healthy foods, especially fruit and vegetables by offering tax relief or reduction of taxes to farmers and traders of healthy foods.

In terms of infrastructure support, the top three priority actions of high importance and feasibility were:

- government monitoring of food environments of all age groups,
- integration of sustainability into Kenya’s food policy for sustainable food systems, and
- Integration of nutrition and health in all stages of government planning and budgeting.

Other policies considered important but less feasible included: tax increases on unhealthy foods and drinks, and trade policy to regulate food safety and nutritional quality in relation to NCDs of imported food.

**Table 3:** Actions identified and prioritized by the expert panel for creating healthier food environments in Kenya.

**Top four Food Policy priorities of highest importance and feasibility**		
1	Food Promotion	Ensure legislation for nutrition standards for processed food industry
2	Food Labelling	Eliminate trans fats as far as possible and label them in packaged foods with warnings using the “traffic lights” labelling
3	Food labelling	Introduce nutrition labelling for processed foods
4	Food Prices	Subsidise the price of healthy foods, especially fruit and vegetables
**Top three infrastructure priorities of highest importance and feasibility**		
1	Monitoring and intelligence	Government should monitor food environments of all age groups
2	Health in all policies	Integrate nutrition and health in all stages of government planning and budgeting
3	Leadership	Integrate sustainability into Kenya’s food policy for sustainable food systems
**Actions of high importance, but less feasible at this present time**		
1	Food Prices	Increase taxes on unhealthy foods and drinks
2	Trade and investment	Trade policy to regulate food safety and nutritional quality of imported food in relation to NCDs

## Discussion

This study has established the extent to which the Kenyan government is implementing policies for healthy food environment along with policy development cycle and against international benchmarks, based on ratings by an expert panel drawn from both government and non-government sectors. We also assessed suggested priorities for future action by government.

Overall, the ratings against the policy development cycle were higher than those against international best practice, with up to one-third of the indicators of government policy action rated to be in the ‘implementation’ phase, and half of the policy indicators rated as in the development stage. No indicator was ranked in the evaluation phase. Comparing against international benchmarks, no indicator was rated as high implementation and there were only four indicators scored as moderate implementation. More than two-thirds (36/43) of the indicators were rated as low or little implementation. These findings suggest that most government policy action to create healthier food environments in Kenya is still at the development stage, with action that is more fully developed not fully meeting international benchmarks. The ratings in our study are much lower than those observed in other LMICs using the same Food-EPI tool. In Thailand, high implementation was reported for 5/30 indicators [19], while in Malaysia, as in Kenya, no indicator was rated as high implementation. However, more indicators (38%) were rated as medium in Malaysia compared to what we found in Kenya (9%) [18].

In the ratings for policy development cycle, five targets were rated as being implemented in the food policy domain compared to 11 in the infrastructure support domain. In the food policy domains, the experts rated for all indicators for food composition and food labelling, and one indicator each for food promotion and food provision in implementation phase, while food prices and food retail policies were considered in development phase. Among infrastructure support domains, the indicators rated as medium included; three indicators under leadership, two indicators under governance, three indicators under monitoring and intelligence, one in funding and resources, one under platforms for interaction, and the target for health in all policies [22]. Most of these policies are driven by the Ministry of Health with some involvement of other stakeholders. Policies regarding food prices were least developed, possibly because of interference from the food industry [23].

In the rating against international benchmarks, the experts only rated restricting breast milk substitutes to be in a moderate implementation phase under the food policy domain. This action may be well implemented as a consequence of support for improving maternal and child health in Kenya, rather than explicit policy commitment on NCD prevention. In the infrastructure support domain, strong visible political support, comprehensive implementation plan linked to national needs, and health in all polices were at medium implementation.

There was coherence in the rating along the policy- development cycle with the rating against international benchmarks. The four areas rated as moderate implementation against international benchmarks included, restricting promotion of breast milk substitutes, strong visible political support, comprehensive implementation plan linked to national needs and health. These policies were also labelled as implementation phase in the development cycle.

Comparing rating of policies by demographic characteristics of the participants revealed striking differences by gender, years of experience, and age of the experts for both policy development cycle and rating against international benchmarks. Women, younger experts less than 35 years of age, and those that had worked for less than 10 years rated policies more positively than their counterparts who were the men, experts older than 35 years and those who worked more than 10 years. This may imply that the older and more experienced experts had a better understanding of policy gaps having experienced them over time compared to younger and less experienced experts whose ratings might be based on limited experience. This diversity emphasizes the importance of capturing a broad range of views in expert panels.

There was also a statistically significant difference between government and non-government experts in rating of policies against international benchmarks, with government actors rating higher than non-government actors. These differences may be a reflection of understanding of the implementation of policies in Kenya, or alternatively, government actors may give a more positive self-assessment due to cognitive and motivational biases [24, 25]. Lack of exposure of non-government actors to actual implementation could also potentially lead to their low rating [26]. In contrast, there was consensus in the ratings of government and non-government actors for the policy development cycle. This is perhaps because most policies are developed in consultation with non-government actors and, as a result, there may be similar levels of understanding.

The experts reached consensus on priority actions for the government to implement in Kenya. There were seven actions prioritized, including four in policy and three on infrastructure support which were rated with the highest importance and feasibility. These focused on ensuring the Kenyan agricultural-food system would be healthier, more sustainable, and financially accessible; for example, a proposal to restrict advertisements of unhealthy foods such as trans fats by enforcement of standards and punitive measures, establishing food composition guidelines and standards for processed foods to guide on energy density for different target groups; offering tax relief or reduction to farmers and traders of healthy foods to promote healthy food consumption; introducing tax policies to favor production and consumption of healthy foods. The infrastructure support actions recommended included putting in place a strong monitoring and intelligence system that will enable government to monitor food environments of all age groups, having health in all policies requiring integration of health and nutrition in all stages of planning and budgeting to highly impact on nutrition including mainstreaming of nutrition in health system and finally taking leadership in Integrating sustainability into Kenya’s food policy for sustainable food systems. The experts felt regulating food prices by increasing taxes on unhealthy foods and drinks and having a trade policy to regulate food safety and nutritional quality for imported food would be important actions to implement but are less feasible to enact.

### Limitations

This study only examined formal national policies and published articles from academic databases but not sub-national policies or community based bye-laws. Kenya has a decentralized system of governance through county governments; some counties could have developed and implemented policies at the county level that were not captured in this study. However, in the Kenyan context the role of policy-making is reserved for central government, with the county taking the role of implementation. This study did not aim to identify how and why policies have or have not been successfully implemented as planned. Such information is needed to design interventions. The process of rating could be judged as subjective, since some participants did not read the evidence pack until the day of the rating workshop, however we addressed this challenge by presenting the evidence pack to the participants for each indicator before rating thus minimizing inter-rater variability. Lastly, personal experience may have overridden the use of the evidence pack during the workshop, which could explain the diversity of ratings by expert’s experience, age and sector, however our inter-rater reliability was within acceptable limits.

## Conclusion

This study has provided a first step in benchmarking government action to improved food environments in Kenya using the Food-EPI tool. It has generated important findings and baseline data to increase the accountability of the government and its partners, by providing evidence on levels of action/inaction on food environments. As such, it represents a step towards identifying and addressing gaps in action to create a healthier food environment in Kenya. The recommended actions are ambitious and will only be met through a collaborative effort between government and non-government actors. The Food-EPI process has established an important baseline and reference point to measure progress in the future. It will be important to repeat this process over time to determine Kenya’s progress in developing and implementing policies for prevention of diet-related NCDs.

## Supporting information

S1 Table Evidence pack for Kenya’s healthy food enviroment policy assessment. (PDF)

S2 Table Raw data collected from national experts. (XLSX)
