# Supplementary material for: Benchmarking food environment policies for the prevention of diet-related non-communicable diseases in Kenya: National expert panel’s assessment and priority recommendations
Source: PLoS One. 2020 Aug 6;15(8):e0236699. doi: 10.1371/journal.pone.0236699 (PMC7410300; doi:10.1371/journal.pone.0236699)
Supplement: S1 Table — (PDF) [file pone.0236699.s001.pdf]

APHRC

African Population and
Health Research Center

INFORMAS

Benchmarking food environments

Assessing government policies and actions to create healthy food environments in Kenya

An International Network for Food and Obesity/Non-Communicable Diseases Research, Monitoring and Action Support (INFORMAS) healthy food environment policy index (Food-EPI) assessment

October 2018

DEFINITIONS

Benchmark: A best practice exemplar or a standard or point of reference, against which aspects of food environments or policies can be assessed and compared.

Civil society: The aggregate of non-governmental organisations, institutions and individuals that manifest interests and will of citizens (academia, professional organisations, public interest NGOs and citizens).

Codex recommendations: The Codex Alimentarius or "Food Code" was established by the Food and Agriculture Organization (FAO) and the World Health Organization (WHO) in 1963 to develop harmonized international food standards, which protect consumer health and promote fair practices in food trade.

Domains: Different aspects of the food environment that can be influenced by governments to create readily accessible, available and affordable healthier food choices, are represented as domains. There are seven domains under a 'policy' component and six domains under an 'infrastructure support' component.

Diet-related non-communicable diseases (NCDs): Type 2 diabetes, cardiovascular diseases and nutrition-related cancers, excluding: micronutrient deficiencies, under-nutrition, stunting, osteoporosis, mental health and gastrointestinal diseases.

Food environments: The collective physical, economic, policy and sociocultural surroundings, opportunities and conditions that influence people's food and beverage choices and nutritional status.

Good practice indicators / statements: Statements that describe the measures (policy actions and infrastructure support) that governments put in place to contribute towards a healthy food environment. There are 43 good practice statements in total.

Government: National and county or local government, including Councils, County Health Boards and Public Health Units.

Government-funded settings: Government departments and agencies, publicly funded schools, publicly funded early childhood education services, elderly homes, hospitals and prisons.

Government implementation: Refers to the intentions and plans of the government, government funding for implementation of actions undertaken by non-governmental organisations, and actions and policies implemented by the government.

Healthy foods: Foods recommended in national food-based dietary guidelines, dietary guidelines or food-based standards.

Healthy food environments: Environments in which the foods, beverages and meals that contribute to a population diet, meeting national dietary guidelines, are widely available, affordably priced and widely promoted.

International examples: International (or sub-national, e.g. regional or city-wide) examples of government measures (policy actions and infrastructure support) that have been put in place and which contribute towards a healthy food environment. The international examples are real-life descriptions that fully or partially equate to the good practice statements.

Nutrients of concern: Salt/sodium, fat, saturated fat, trans fat (trans fatty acids), added sugar.

Platforms: Formal government mechanisms (e.g. standing committees, ad hoc committees, advisory groups, taskforces, boards, joint appointments) for interaction on particular issues.

Population nutrition promotion: Population promotion of healthy eating and healthy food environments for the prevention of obesity and diet-related NCDs, excluding all one-on-one promotion (primary care, antenatal services, maternal and child nursing services etc.), food safety, micronutrient deficiencies (e.g. folate fortification) and under-nutrition.

Unhealthy foods: Processed foods or non-alcoholic beverages high in saturated fats, trans fats (trans fatty acids), and added sugars or salt/sodium.

INTRODUCTION

Africa is experiencing a nutrition transition, characterised by changing dietary patterns that are related to rapid urbanisation (1). The overconsumption of unhealthy (energy-dense and nutrient-poor) foods is implicated in the onset and progression of diet-related non-communicable diseases (NCDs) (2,3). Unhealthy diets are also associated with lower micronutrient intake, which remains prevalent in Africa (4,5). As a consequence, obesity and diet-related NCDs are rapidly increasing and becoming a critical public health problem (3). Kenya exemplifies this trend in the African region, whereby NCDs account for about a third of all deaths and half of all hospital admissions, and are now recognised as a pressing public health concern; as highlighted in the Kenya National Strategy for the Prevention and Control of Non-Communicable Disease (6). Dietary transitions, resulting from rapidly changing social and physical environments, and changing food habits or practices, driven in part by a proliferation of so called 'fast-foods' that are often high in saturated fats, trans-fats, salt, and sugar, have been documented in Kenya (7,8). This, combined with reduced energy expenditure, which is increasingly and particularly common among urban dwellers, means that NCDs and their risk factors are advancing more rapidly (8,9).

Women are at considerably higher risks for NCDs. In Kenya, the prevalence of overweight and obesity among women increased by about 10 fold (25% to 33%) from 2008 to 2014 (10). The Kenya National Strategy for the Prevention and Control of Non-Communicable Diseases calls for improved policy formulation, legislation, and interventions to promote healthy diets as a key strategy in the fight against NCDs. The strategy document however, points to a paucity of data to facilitate appropriate planning, policy formulation, regulations, and legislation, in empowering individuals to make informed decisions with regards to prevention and control of NCDs. For successful development or strengthening of policies and interventions, it is important that we first understand what NCD-related nutrition polices exist and, crucially, as part of this, to understand how government is working to create healthy food environments, which can be defined as:

> *the collective physical, economic, policy and sociocultural surroundings, opportunities and conditions that influence people's food and beverage choices and nutritional status.*

We need to understand the extent of implementation of policy and actions to improve food environments in Kenya, in order to ensure that government actions match the magnitude of the burden that unhealthy diets creates.

Realising that comprehensive actions by governments and the food industry are needed to achieve global targets to halt the rise in obesity and diet-related NCDs, the International Network for Food and Obesity/Non-Communicable Diseases Research, Monitoring and Action Support (INFORMAS) was formed to monitor and benchmark food environments, relevant government policies, and private sector actions, with the goal of increasing accountability and action by relevant stakeholders. INFORMAS developed a Healthy Food Environment Policy Index (Food-EPI) tool and process for use in assessing the extent of government policy action and related infrastructure support in comparison to international best practice in the creation of healthy food environments to reduce obesity, NCDs, and their related inequalities. Benchmarking the extent of government policy implementation on food environments among countries internationally has the potential to support increased government actions globally to reduce obesity and diet-related NCDs. Indeed, a key goal of the Food-EPI assessment is to identify and prioritise actions needed to address critical gaps in food environment policy so as to contribute to addressing obesity and NCDs (11).

The Food-EPI assessment comprises a 'policy' component with seven domains on specific aspects of food environments, and an 'infrastructure support' component with six domains to strengthen systems to prevent obesity and diet-related NCDs (Figure 1).

Figure 1. The Healthy Food Environment Policy Index (Food-EPI)

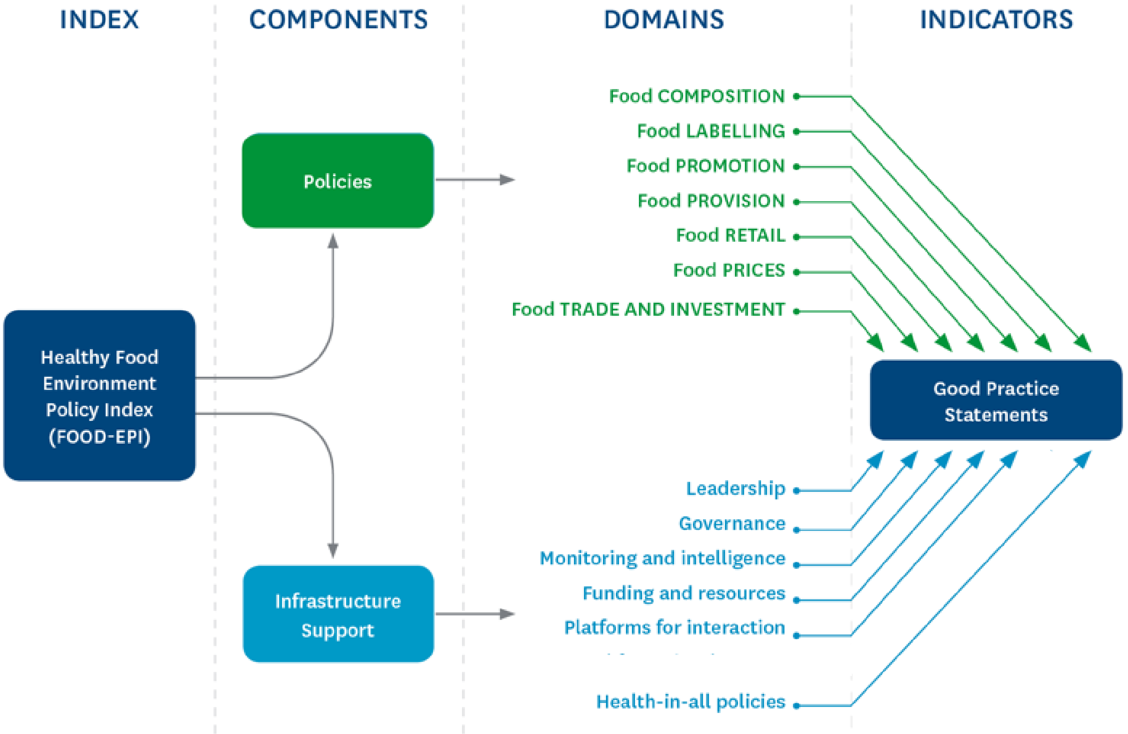

The domains included were derived from an INFORMAS review of policy documents and revised through a week-long consultation process with international experts, including experts from low and middle-income countries. The domains included in the 'infrastructure support' component are based on the WHO 'system building blocks' approach for health systems. The proposed Food-EPI is consistent with and supportive of the list of proposed policy options for Member States included in the WHO's Global Action Plan for the Prevention and Control of NCDs (2013–2020) and the World Cancer Research Fund (WCRF) International NOURISHING Food Policy Framework for Healthy Diets. For each of the 13 domains, good practice indicators are proposed, and for each of these, benchmarks or best practice exemplars that have been introduced by governments at the forefront of creating and implementing food policies for good health, have been collected.

You have been invited to take part in an expert panel workshop to implement the Healthy Food Environment Policy Index (Food-EPI) in Kenya.

The overarching question we are hoping to answer through the Food-EPI process is:
How much progress has the Kenyan government made towards best practice in improving food environments and implementing obesity/NCD prevention policies and actions?

METHODS FOR THE IMPLEMENTATION OF THE FOOD-EPI

First step: Collation of evidence of government policy action to improve healthy food environments
The **first key step** in the Food-EPI process entailed the collection of relevant policy documents relating to healthy food environment and supporting evidence of policy action and implementation for all of the 43 good practice indicators of the Food-EPI (across the 13 Food-EPI domains). This collated evidence has been validated by government officials, non-government experts, and researchers, to ensure that no evidence had been overlooked or misrepresented. The collated evidence is presented in this current evidence pack by domain and by the associated good practice indicators. This **evidence document** is an attempt to collate all relevant legislation, policies, strategies, and evidence of their implementation for each of the domains.

Second step: National expert panel meeting
The **second key step** of the process involves rating of government action and also the identification and prioritisation of future actions that the Kenya government could implement to improve the healthiness of food environments in the country. This is achieved at a face-to-face, national expert panel meeting.

You have been invited to take part in this second key step – the expert panel meeting - and will be asked to rate government policy action (based on the evidence that is presented in this evidence pack) as well as identify and prioritise future actions that the Kenyan government could implement to improve the healthiness of food environments.

You will be asked to rate government policy actions against 1) the development cycle and also 2) in relation to international best practice benchmarks.

Instructions for rating within the expert panel meeting

You will be asked to assess government policy for each good practice indicator using two rating scales, with rating for both completed electronically on a hand-held device, and with responses analysed immediately. The rating scales that you are asked to use are:

1) Rating scale to assess government action in relation to the development cycle:
   You are asked to consider the evidence provided for each good practice indicator and use your expert judgement to rate where the Kenyan government policy action lies along the following development cycle:

   1. Initiation phase
   2. Development phase
   3. Implementation phase
   4. Evaluation phase
   5. Cannot rate

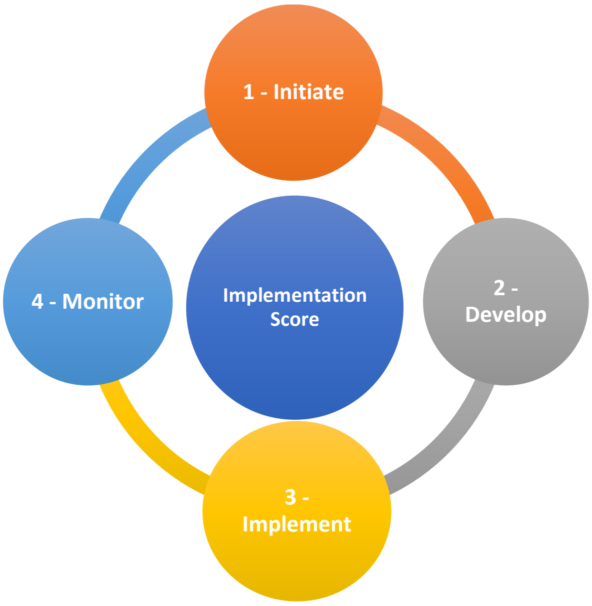

The option of ‘cannot rate’ is also possible for cases where you do not feel comfortable to rate.

The rating you give should reflect your personal view of which stage within this cycle you think Kenyan policy action currently is.

2) Rating scale to assess government actions in relation to international best practice benchmarks:
You are asked to consider the evidence provided for each good practice indicator and your expert judgement to rate Kenyan government policy action in relation to the international best practice exemplars that are provided within this evidence pack, using the following rating scale:

1. <20% implementation, Very Low to No progress in relation to best practice
2. 20-40% implementation, Low to Medium progress in relation to best practice
3. 40-60% implementation, Good progress in relation to best practice
4. 60-80% implementation, Very Good progress in relation to best practice
5. 80-100% implementation, Excellent progress in relation to best practice
6. Cannot rate

As above, the option of ‘cannot rate’ is an option for cases where you do not feel comfortable to rate.

Your rating score should reflect current level of government implementation rather than any change or improvement over time. In addition, policies and actions should not be confused with outcomes (such as population nutrition and obesity and NCD prevalence).

FOOD-EPI ASSESSMENT DOMAINS

1 FOOD COMPOSITION 10
2 FOOD LABELLING 13
3 FOOD PROMOTION 17
4 FOOD PRICES 22
5 FOOD PROVISION 26
6 FOOD IN RETAIL 31
7 FOOD TRADE AND INVESTMENT 33
8 LEADERSHIP 36
9 GOVERNANCE 42
10 MONITORING AND EVALUATION 45
11 FUNDING AND RESOURCES 49
12 PLATFORMS FOR INTERACTION 51
13 HEALTH IN ALL POLICIES 54

Food Environment Policy Index: POLICY Domains

DOMAIN 1 - FOOD COMPOSITION (COMP)

There are government systems implemented to ensure that, where possible, processed foods minimise the energy density and nutrients of concern (e.g. salt, fat, saturated fat, trans-fat, added sugar).

Good Practice Indicator - COMP 1: Food composition targets/standards have been established for processed foods by the government for the content of the nutrients of concern in certain foods or food groups if they are major contributors to population intakes of these nutrients of concern (trans-fats and added sugars in processed foods, salt in bread salt in snacks etc.).

Evidence of policy action:

A food fortification strategy is stipulated as one of the ways of addressing micronutrient deficiencies. This is included in the Kenya Food and Nutrition Security Policy (2011) and the amendment of the Food Drugs and Chemical Substance Act 2012, which made it mandatory for packaged wheat flour, maize meal, fats/oils to be fortified following the East African Standards for fortification. **However**, these standards do not address food composition standards for processed foods on the use of fats, salts, or sugars in relation to NCDs.

The Food, Drugs and Chemical Substances Act Cap 254 (Amendment) 2015 dictates that edible vegetable fats and oils shall be free from trans fatty acids, and that food deemed to be free from trans fatty acids shall contain such amounts of trans fatty acids as may be permitted by the Codex Allimentarius (0.1 g trans-fat per 100 g or per 100 ml).

Development of an updated Food Composition Table for Kenya has been completed and will be launched in August 2018, led by the Ministry of Health and the Ministry of Agriculture, Livestock and Fisheries in Kenya and the Food and Agriculture Organization of the United Nations (FAO) in consultation with contributors from various academic and research institutions, laboratories, and programmes. The updated food composition table has more elaborate presentation of the nutrient information and contains more nutrient components compared to the older version that was developed in 1993.

The Kenya National Strategy for Preventions and Control of NCDs recommends a 15% reduction of salt intake at population level, as a means of promoting healthy diets and reducing the modifiable risk factors for non-communicable diseases

International best practice examples (benchmarks)

ARGENTINA: In December 2013, the Government adopted a law on mandatory maximum levels of sodium permitted in meat products and their derivatives, breads and farinaceous products, soups, seasoning mixes and tinned foods (Act 26905) which entered into force in December 2014. The law is applicable to salt levels in restaurant dishes. The law includes gradual reductions (between 5% and 18% of reduction). Infringements by producers and importers may be sanctioned, the most severe penalties being fines of up to one million pesos, in case of repeat infringements up to ten million pesos, and the closing of the business for up to five years (1, 2). The text of the legislation and specific reduction targets can be found on the Ministry of Health of Argentina website (3). The legislation is embedded into a wider initiative (Less Salt, More Life) which also includes the reduction of salt in processed foods through voluntary agreements with manufacturers, retailers and bakers, and public awareness of the health effects and the need to reduce discretional salt. To date about 60 companies representing 487 processed food products and more than 9000 bakeries have signed the voluntary agreement (2).

SOUTH-AFRICA: In 2013, the South African Department of Health adopted mandatory targets for salt reduction in 13 food categories (including bread, breakfast cereals, margarines and fat spreads, savoury

snacks, processed meats as well as raw-processed meat sausages, dry soup and gravy powders and stock cubes) by means of regulation (Foodstuffs, Cosmetics and Disinfectants Act). There is a stepped approach with food manufacturers given until June 2016 to meet one set of category-based targets and another three years until June 2019 to meet the next (2, 4). The specific reduction targets for each of the food groups can be found in the Staatskoerant of 20 March 2013 (5).

DENMARK: A law introduced in 2003 prohibits the sale of products containing *trans*-fats, a move that effectively bans its use in products destined for sale on the Danish market (2, 6). The law is enforced by local authorities under the supervision of the Danish Veterinary and Food Administration. Infringement of the law may incur a fine or imprisonment, and companies can be prosecuted according to the Danish Penal Code.

EU & UK: In 2012, under the directive 2012/12/EU of The European Parliament and the Council, an amendment of Council Directive 2001/112/EC outlined that addition of sugars is no longer authorised in fruit juice (7). Similarly, added sugar in fruit juice is no longer permitted under The Fruit Juices and Fruit Nectars Regulations 2013 (8).

UK: In August 2016 the Government published 'Childhood obesity: A plan for action'. This included a commitment for Public Health England (PHE) to oversee a sugar reduction programme. This challenges all sectors of the food industry to reduce by 20% by 2020 the level of sugar in the categories that contribute most to the intakes of children up to 18 years. Industry was also challenged to achieve a 5% reduction in the first year of the Programme. An assessment of progress, based on analysis of data for the year ending August/September 2017, compared to the baseline year of 2015 was done. There have been reductions in sugar levels in 5 out of the 8 food categories where progress has been measured. for retailers own brand and manufacturer branded products there has been a 2% reduction in total sugar per 100g.Reference: https://assets.publishing.service.gov.uk/government/uploads/system/uploads/attachment_data/file/709008/Sugar_reduction_progress_report.pdf

FRANCE: As part of the French National Nutrition and Health Programme (PNNS), the Ministry of Health established a Charter of Engagement with the food industry (2008). One area of action is improving the nutritional composition of food products by reducing the amount of salt, sugar, total and saturated fats and increasing the amount of fibre. Any entity with an economic interest in the food industry is eligible to submit nutritional commitments. Nine principles are detailed: compliance, honesty, efficiency, retroactivity, fairness, transparency, monitoring, updating, and confidentiality. Commitments must be clear, accurate, precise, dated, and controllable. To date, over 35 companies have made voluntary commitments, which are reviewed and approved by an external committee of 24 public sector experts to ensure they are "significant". There is a strict follow-up. The approved charters are signed by the food industry and monitored by the Food Quality Observatory (created in 2008) (2, 9).

Good Practice Indicator - COMP 2: Food composition targets/standards have been established by the government for out-of-home meals in food service outlets (such as fast food joints, food kiosks, check-check joints, restaurants, and other local food vendors) for the content of the nutrients of concern in certain foods or food groups if they are major contributors to population intakes of these nutrients of concern (e.g. trans fats, added sugars, salt, saturated fat, saturated fat (e.g. for commercial frying fats/oils).

Evidence of policy action:
None found.

International best practice examples (benchmarks)

US: In June 2015, the US Food and Drug Administration determined that partially hydrogenated oils (PHOs), the primary source of trans fats, are not "generally recognized as safe (GRAS)" for any use in food. Food manufacturers had three yeas to remove them from products. As of 18 June 2018, food manufacturers and restaurants are no longer allowed to produce foods that contain PHOs. (13.)

NEW YORK: In 2009, New York City established voluntary salt guidelines for various restaurant and store-bought foods. In 2010, this city initiative evolved into the National Salt Reduction Initiative that encouraged nationwide partnerships among food manufacturers and restaurants involving more than 100 city and state health authorities to reduce excess sodium by 25% in packaged and restaurant foods. The goal is to reduce Americans' salt intake by 20% over five years. The National Salt Reduction Initiative has worked with the food industry to establish salt reduction targets for 62 packaged foods and 25 restaurant food categories for 2012 and 2014. The commitments and achievements of companies have been published online (14).

NEW ZEALAND: In New Zealand, The Chip group, funded 50% by the Ministry of Health and 50% by industry, aims to improve the nutritional quality of deep-fried chips served by food service outlets by setting an industry standard for deep frying oils. The standard for deep frying oil is maximum 28% of saturated fat, 3% linoleic acid and 1% of *trans*-fat. The Chip group oil logo for use on approved oil packaging was developed in 2010 (15).

THE NETHERLANDS: On January 2014, the Dutch Ministry of Health, Welfare and Sport signed an agreement with trade organizations representing food manufacturers, supermarkets, hotels, restaurants, caterers and the hospitality industry to lower the levels of salt, saturated fat and calories in food products. The agreement includes ambitions for the period up to 2020 and aims to increase the healthiness of the food supply (2, 16).

DOMAIN 2 - FOOD LABELLING (LABEL)

There is a regulatory system implemented by the government for consumer-oriented labelling on food packaging and menu boards in food outlets to enable consumers to easily make informed food choices and to prevent misleading claims.

Good Practice Indicator - LABEL1: Ingredient lists and nutrient declarations in line with Codex recommendations are present on the labels of packaged foods.

Evidence of policy action:

The Draft East African Standards (2015), Nutrition Labelling - Requirements [KS EAS 803:2014] attempt to harmonise regional activities and build client oriented infrastructure complying with international standards in East African Countries (Burundi, Kenya, Rwanda, Tanzania, and Uganda). They provide the specifications for labelling of food products, including ingredient lists/nutrition information panels, as details that should be included in food labels. These standards, developed for individual food categories and items, have been adopted and are implemented by the Kenya Bureau of Standards (KEBS). The draft document is currently available for public (consumers) review.

The Food, Drugs and Chemical Substances Act Cap 254 (Amendment) 2015 requires that all foods containing edible fats or oils shall declare the actual level of trans fatty acids in the front of the package label. It also dictates that labelling of foods free from trans fatty acids should be done in accordance with the Kenya Bureau of Standards, Draft East African Standards, and other national standards.

The Kenya Bureau of Standards (KEBS) is a statutory body established under the Standards Act (CAP 496) of the laws of Kenya, and that commenced its operations in 1974. KEBS is charged with providing tests in areas of biochemistry, microbiology, food and agriculture, engineering, and textiles. KEBS is responsible for monitoring food ingredients and testing nutrient declarations, including random tests of foods available in the market. The KEBS standard KS EAS 38 addresses general labelling of processed food that is, requiring a listing of ingredients and provision of nutrition profile.

The Kenya National Strategy for Preventions and Control of NCDs strategy objective 3, focuses on promoting healthy lifestyles and implementing interventions to reduce the modifiable risk, factors for non-communicable diseases, and recommends the implementation of health related legislations and regulations on salt, saturated and trans fatty acids and refined sugar content of processed foods and the packaging, labeling and marketing of food products and beverages.

International best practice examples (benchmarks) for LABEL1

MANY COUNTRIES: In a wide range of countries producers and retailers are required by law to provide a comprehensive nutrient list on pre-packaged food products (with limited exceptions), even in the absence of a nutrition or health claim. The rules define which nutrients must be listed and on what basis (e.g. per 100g/per serving) (20).

SOME COUNTRIES: A more limited number of countries (about N=10) require that nutrient lists on pre-packaged food must, by law, include the *trans*-fat content of the food. Specific rules generally define how the *trans*-fat content must be listed, and on what basis (e.g. per 100g/100ml or per serving). If the *trans*-fat content falls below a certain threshold, it may be listed as 0g (e.g. less than 0.5g per serving, or less than 0.3g per 100g of food product)(20).

US: The US Food and Drug Administration proposed updates to the Nutrition Facts label on food packages. Information on the amount of added sugars (in grams and as percent Daily Value) now needs to be included on the label, just below the line for total sugars (21).

Good Practice Indicator - LABEL2: Robust, evidence-based regulatory systems are in place for approving/reviewing claims on foods, so that consumers are protected against unsubstantiated and misleading nutrition and health claims.

Evidence of policy action:

The Draft East African Standards (2015), Use of Nutrition and Health Claims - Requirements [KS EAS 804 and KS EAS 805:2014] provide the specifications against which claims on foods may be tested. KEBS is responsible for reviewing and approving claims on food, and monitoring food ingredients and testing nutrient declarations, including random tests of foods available in the market for the protection of misleading nutrition and health claims.

The Advertising Standards Body of Kenya - Code of Advertising Practice and Direct Marketing 2003, was an initiative of the Marketing Society of Kenya (MSK) and the Association of Practitioners in Advertising (APA), aiming to create a review process leading to the adoption of a comprehensive and up-to date Advertising Code of Practice and Guiding Principles for the Kenyan Market. While self-regulated, the actions of this body are subject to and guided by Kenyan law. The goal is to provide a tangible framework under which all professional parties in the marketing process forge a responsible attitude. Promotional content of display material, menus, labels and packaging also fall within the definition.

The Kenya National Strategy for Preventions and Control of NCDs , objective 3, focuses on promoting healthy lifestyles and implement interventions to reduce the modifiable risk, factors for non-communicable diseases, and recommends the implementation of health related legislations and regulations on salt, saturated and trans fatty acids and refined sugar content of processed foods and the packaging, labeling and marketing of food products and beverages.

International best practice examples (benchmarks) for LABEL2

AUSTRALIA/NEW ZEALAND: A law (Standard 1.2.7)(25), approved in 2013, regulates the use of nutrition content and health claims on food labels in Australia and New Zealand. Health claims must be based on pre-approved food-health relationships or self-substantiated according to government requirements and they are only permitted on foods that meet nutritional criteria, as defined by a nutrient profiling model (Nutrient Profiling Scoring Criterion (NPSC)) taking into account energy, sodium, saturated fat and total sugar content of foods, as well as protein, fibre, fruit, vegetable, nut and legume content of foods. Although nutrition content claims also need to meet certain criteria set out in the Standard, there are no generalized nutritional criteria that restrict their use on "unhealthy" foods such as for health claims. The industry needed to comply with this new legislation by January 2016. Food Standards Australia New Zealand has developed an online calculator to help food businesses to calculate a food's nutrient profiling score(26).

INDONESIA: Regulation HK.03.1.23.11.11.09909 (2011) (27) on "The Control of Claims on Processed Food Labelling and Advertisements" establishes rules on the use of specified nutrient content claims (i.e. levels of fat for a low fat claim). The Regulation applies to any food product or beverage which has been processed. Generally, any nutrition or health claim may only be used on processed foods or beverages if they do not exceed a certain level of fat, saturated fat and sodium per serving (13g total fat, 4g saturated fat, 60mg cholesterol and 480mg sodium). The Regulation sets out certain exceptions from this rule, whereby products exceeding these limits may still contain certain nutrient or health claims ("low in [name of nutrient]" and "free from [name of nutrient]" claims; claims related to fibre, phytosterol and phytostanol; certain disease risk reduction claims)(20).

US: Nutrient-content claims are generally limited to a list of nutrients authorized by the Food and Drug Administration (Food Labelling Guide 1994, as last revised in January 2013). Packages containing a nutrient-content claim must include a disclosure statement if a serving of food contains more than 13g of fat, 4g of saturated fat, 60mg of cholesterol, or 480mg of sodium. Health claims are generally not permitted if a food contains more than 13g of fat, 4g of saturated fat, 60mg of cholesterol, or 480mg of sodium. Sugar and whole grain content are not considered (20, 28).

Good Practice Indicator - LABEL3: A single, consistent, interpretive, evidence-informed front-of-pack supplementary nutrition information system, which readily allows consumers to assess a product's healthiness, is applied to packaged foods.

Evidence of policy action:
None found.

International best practice examples (benchmarks)for LABEL3:

FRANCE: On 26 January 2016, the French Ministry of Health introduced Article 5 of the Health Act that recommended introducing a system of nutrition labelling. The Directorate-General for Health requested public health France to design the nutrition labelling and the decision to recommend the Nutriscore system was informed by research that trialled four different types of nutrition labels in 80 supermarkets. The Nutriscore label uses a nutrient profiling system based on the UK Food Standards Agency model. It classifies foods and drinks according to five categories of nutritional quality indicated via a colour scale ranging from dark green to dark red. Each colour is also associated with a letter from A (dark green) to E (dark red). The score takes into account for every 100grams of product whether the contents of the product include nutrients and food that should be favoured (fibre, protein, fruits and vegetables) or nutrients that should be limited (energy, saturated fat, sugars and salt). The amount of nutrients per 100g is scored using a points systems (0-40 for negative nutrients and 0-15 for positive nutrients). The score is then calculated by subtracting the negative nutrient points from the positive nutrient points.

UK**:** Traffic light labelling has been recommended for use in the UK since 2006. In 2013, the Government published national guidance for voluntary 'traffic light' labelling for use on the front of pre-packaged food products. The label uses green, amber and red to identify whether products contain low, medium or high levels of energy, fat, saturated fat, salt and sugar. The format of the label and thresholds for nutrients of concern for red, amber and green can be found elsewhere (29). A combination of colour coding and nutritional information is used to show how much fat, salt and sugar and how many calories are in each product. The voluntary scheme is used by all the major retailers and some manufacturers (29). Traffic lights are displayed on about two thirds of UK food products.

ECUADOR: A regulation of the Ministry of Public Health published in November 2013 (No. 4522, El Reglamento de Etiquetado de Alimentos Procesados) requires packaged foods to carry a "traffic light" label in which the levels of fats, sugar and salt are indicated by red (high), amber (medium) or green (low). Full compliance with the regulation was required by 29 August 2014 (20). The legislation including format of the label and thresholds for nutrients of concern for red, amber and green can be found online (30).

AUSTRALIA/NEW ZEALAND: The government approved a 'Health Star Rating' (HSR) system as a voluntary scheme for industry adoption. The system takes into account four aspects of a food associated with increasing risk for chronic diseases; energy, saturated fat, sodium and total sugars content along with certain 'positive' aspects of a food such as fruit and vegetable content, and in some instances, dietary fibre and protein content. Star ratings range from ½ star (least healthy) to 5 stars (most healthy). Implementation of the HSR system began in June 2014 and is overseen by the Australia and New Zealand Ministerial Forum on Food Regulation, the Front-of-Pack Labelling Steering Committee, the Trans-Tasman Health Star Rating Advisory Committee, the New Zealand Health Star Rating Advisory Group and a recently established Technical Advisory Group. The Technical Advisory Group is currently evaluating progress as well as conducting a formal review of the HSR system, including an assessment of the underlying algorithm. In New Zealand, as of March 2016, about 900 products have stars on them (March 2016) (31).

CHILE: In 2012, the Chilean Government approved a Law of Nutritional Composition of Food and Advertising (Ley 20, 606)(32). In June 2015, the Chilean authority approved the regulatory norms required for the law's implementation (Diario Oficial No 41.193). The regulatory norms define limits for calories (275 calories/100g or 70 calories/100mL), saturated fat (4g/100g or 3g/100mL), sugar (10g/100g or 5g/100mL) and sodium (400mg/100g or 100mg/100mL) content considered "high" in foods and beverages. All foods that exceed these limits need to have a front-of-package black and white warning

message inside a stop sign that reads “HIGH IN” followed by CALORIES, SATURATED FAT, SUGAR or SODIUM, as well as “Ministry of Health”. A warning message needs to be added to products per nutrient of concern exceeding the limit (e.g. a product high in fat and sugar will have 2 stop signs). The regulatory norms provide specifications for the size, font, and placement of the warning message on products. The limits for calories, saturated fat, sugar and sodium are being implemented using an incremental approach, reaching the defined limits by 1 July 2018 (20). Although no studies are available yet, the regulation is reported to be already well implemented with many products already carrying the warning labels.

Good Practice Indicator - LABEL4: A consistent, single, simple, clearly-visible system of labelling the menu boards of quick service restaurants (i.e. fast food chains) is applied by the government, which allows consumers to interpret the nutrient quality and energy content of foods and meals on sale.

Evidence of policy action:
None found.

International best practice examples (benchmarks) for LABEL4

SOUTH KOREA: Since 2010, the Special Act on Safety Control of Children’s Dietary Life has required all chain restaurants with 100 or more establishments to display nutrient information on menus including energy, total sugars, protein, saturated fat and sodium (20).

TAIWAN: Since July 2015, convenience store chains, drink vendor chains and fast food chains have to label the sugar and caffeine content of prepared-when-ordered drinks (e.g. coffee-and-tea-based drinks, fruit and vegetable juices) according to a regulation based on the Food Safety and Sanitation Act. The amount of sugar added to drinks (specified in sugar cubes) and its calorie content have to be displayed on drink menus and/or notice boards in a prescribed minimum font. In addition, different colours have to be used to signal the level of caffeine contained in coffee drinks (20).

US: Section 4205 of the Patient Protection and Affordable Care Act (2010) (35) requires that all chain restaurants with 20 or more establishments display energy information on menus. The implementing regulations were published by the Food and Drug Administration on 1 December 2014. Implementation was delayed several times but implemented in May 2018. Two states (California and Vermont), seven counties (e.g. King County, WA and Albany County NY) and two municipalities (e.g. New York City, Philadelphia) have already implemented regulations requiring chain restaurants (often chains with more than a given number of outlets) to display calorie information on menus and display boards. These regulations will be pre-empted by the national law once implemented; local governments will still be able to enact menu labelling regulations for establishments not covered by national law. The regulations also require vending machine operators of more than 20 vending machines to post calories for foods where the on-pack label is not visible to consumers by 26 July 2018 (20).

AUSTRALIA: Legislation in Australian Capital Territory (Food Regulation 2002) and the States of New South Wales (Food Regulation 2010), Queensland (Food Regulation 2017) and South Australia (Food Regulation 2002) requires restaurant chains (e.g. fast food chains, ice cream bars) with ≥20 outlets in the state (or seven in the case of ACT), or 50 or more across Australia, to display the kilojoule content of food products on their menu boards. Average adult daily energy intake of 8700kJ must also be prominently featured. Other chains/food outlets are allowed to provide this information on a voluntary basis, but must follow the provisions of the legislation (20).

NEW YORK: Following an amendment to Article 81 of the New York City Health Code (addition of section 81.49), chain restaurants are required to put a warning label on menus and menu boards, in the form of a salt-shaker symbol (salt shaker inside a triangle), when dishes contain 2,300 mg of sodium or more. It applies to food service establishments with 15 or more locations nationwide. In addition, a warning statement is required to be posted conspicuously at the point of purchase: “Warning: [salt shaker symbol] indicates that the sodium (salt) content of this item is higher than the total daily recommended

limit (2300 mg). High sodium intake can increase blood pressure and risk of heart disease and stroke." This came into effect 1 December 2015 (20, 36).

DOMAIN 3 - FOOD PROMOTION (PROMO)

There is a comprehensive policy implemented by the government to reduce the impact (exposure and power) of promotion of unhealthy foods to or for children across all media.

Good Practice Indicator - PROMO1: Effective policies are implemented by the government to restrict exposure and power of promotion of unhealthy foods to or for children through broadcast media (TV, radio).

Evidence of policy action for PROMO1:

The Advertising Standards Body of Kenya - Code of Advertising Practice and Direct Marketing 2003 (Parts I and II) states that in advertising targeted at children, it should be realized that because of the credulity and lack of experience of a child, the Code will be interpreted much more narrowly, as children would be likely to attach or associate a more literal meaning to advertising. The Advertising Standard Body of Kenya is responsible for the implementation of these restrictions.

- It restricts the advertisements addressed to or targeted towards children or that are likely to influence children, that contain any statements or visual presentations which might result in harming them mentally, morally, physically or emotionally.
- Advertisements should not exploit the natural credulity or gullibility of children or their lack of experience and should not strain their senses of loyalty.
- Promotional content of display material, menus, labels and packaging also fall within the definition.

However, this act is not specific to restrictions on unhealthy foods.

Development of policies to reduce the impact on children of marketing of foods and nonalcoholic beverages high in saturated fats, trans fatty acids, free sugars, or salt , is a key indicator in the Kenya National Strategy for prevention and Control of NCDs' monitoring frame work

International best practice examples (benchmarks) for PROMO1:

NORWAY/SWEDEN: Under the Broadcasting Act, advertisements (food and non-food) may not be broadcast on television directed to children or in connection with children's programs. This applies to children 12 years and younger (41).

QUEBEC (CANADA): In Quebec, most citizens speak French and it is the only province in Canada, where children below 13 years old are protected under the Consumer Protection Act since 1980 (42). In Québec, the Consumer Protection Act prohibits commercial advertising (including food and non-food) directed at children less than 13 years of age through television, radio and other media. To determine whether or not an advertisement is directed at persons under thirteen years of age, account must be taken of the context of its presentation, and in particular of: a) the nature and intended purpose of the goods advertised; b) the manner of presenting such advertisement; and c) the time and place it is shown. A cut-off of 15% share of children audience is used to protect children from TV advertising (43). Any stakeholder involved in a commercial process (from the request to create an advertisement to its distribution, including its design) may be accused of not complying with the legislation in force. Per indictment, that person is liable to: a fine ranging from $600 to $15,000 (in the case of a natural person); a fine ranging from $2,000 to $100,000 (in the case of a legal person). Notably, for the rest of Canada, child-directed food marketing is self-regulated using the Canadian Children's Food and Beverage Advertising Initiative (CAI) by Advertising Standards Canada (ASC) through The Broadcast Code for Advertising to Children.

CHILE: In 2012, the Chilean government approved a Law of Nutritional Composition of Food and Advertising (Ley 20, 606)(32). In June 2015, the Chilean authority approved the regulatory norms required for the law's implementation (Diario Oficial No 41.193). The regulatory norms define limits for calories, saturated fat, sugar and sodium content considered "high" in foods and beverages. The regulatory norms define limits for calories (275 calories/100g or 70 calories/100mL), saturated fat (4g/100g or 3g/100mL), sugar (10g/100g or 5g/100mL) and sodium (400mg/100g or 100mg/100mL) content considered "high" in foods and beverages. The law restricts advertising directed to children under the age of 14 years of foods in the "high in" category. The regulatory norms define advertising targeted to children as programmes directed to children or with an audience of greater than 20% children, and according to the design of the advertisement. Promotional strategies and incentives, such as cartoons, animations, and toys that could attract the attention of children are included in the ban. The regulation is scheduled to take effect 1 July 2016 (41). Chile outlaws Kinder Surprise eggs and prohibit toys in McDonald's 'Happy Meals' as part of this law (44).

IRELAND: Advertising, sponsorship, teleshopping and product placement of foods high in fats, sugars and salt, as defined by a nutrient profiling model, are prohibited during children's TV and radio programmes where over 50% of the audience are under 18 years old (Children's Commercial Communications Code, 2013 revision). In addition, there is an overall limit on advertising of foods high in fats, sugars and salt adverts at any time of day to no more than 25% of sold advertising time and to only one in four advertisements. Remaining advertising targeted at children under the age of 13 must not include nutrient or health claims or include licensed characters (41).

SOUTH KOREA: TV advertising to children less than 18 years of age is prohibited for specific categories of food before, during and after programmes shown between 5-7pm and during other children's programmes (Article 10 of the Special Act on the Safety Management of Children's Dietary Life, as amended 2010) (41, 45).

Good Practice Indicator - PROMO2: Effective policies are implemented by the government to restrict exposure and power of promotion of unhealthy foods to or for children through non-broadcast media (e.g. Internet, social media, food packaging, sponsorship, religious events, outdoor advertising including around schools).

Evidence of policy action for PROMO2:

The Advertising Standards Body of Kenya - Code of Advertising Practice and Direct Marketing 2003 (Parts I and II) states that in advertising targeted at children, it should be realized that because of the credulity and lack of experience of a child, the Code will be interpreted much more narrowly, as

children would be likely to attach or associate a more literal meaning to advertising. The Advertising Standard Body of Kenya is responsible for the implementation of these restrictions.

- It restricts the advertisements addressed to or targeted towards children or that are likely to influence children, that contain any statements or visual presentations which might result in harming them mentally, morally, physically or emotionally.
- Advertisements should not exploit the natural credulity or gullibility of children or their lack of experience and should not strain their senses of loyalty.
- Promotional content of display material, menus, labels and packaging also fall within the definition.

However, this act is not specific to restrictions on unhealthy foods.

- The draft school health policy (2017) prohibits the promotion of unhealthy foods in schools and marketing of any foods and beverages around the schools, it recommends that the schools actively promotes healthy foods instead.
- Development of policies to reduce the impact on children of marketing of foods and nonalcoholic beverages high in saturated fats, trans fatty acids, free sugars, or salt , is one of the key indicator in the Kenya National Strategy for prevention and Control of NCDs' monitoring frame work.
- The World Health organization and the Ministry of Health are in the process of piloting tools for profiling foods that are marketed for children. The tool will be useful in making decisions on the foods whose marketing should be restricted.

International best practice examples (benchmarks) for PROMO2:

CHILE: In 2012, the Chilean government approved a Law of Nutritional Composition of Food and Advertising (Ley 20, 606)(32). In June 2015, the Chilean authority approved the regulatory norms required for the law's implementation (Diario Oficial No 41.193). The regulatory norms define limits for calories, saturated fat, sugar and sodium content considered "high" in foods and beverages. The regulatory norms define limits for calories (275 calories/100g or 70 calories/100mL), saturated fat (4g/100g or 3g/100mL), sugar (10g/100g or 5g/100mL) and sodium (400mg/100g or 100mg/100mL) content considered "high" in foods and beverages. The law restricts advertising directed to children under the age of 14 years of foods in the "high in" category. The regulatory norms define advertising targeted to children as websites directed to children or with an audience of greater than 20% children, and according to the design of the advertisement. Promotional strategies and incentives, such as cartoons, animations, and toys that could attract the attention of children are included in the ban. The regulation took effect 1 July 2016 and applies to all advertising media (41). Chile outlaws Kinder Surprise eggs and prohibit toys in McDonald's 'Happy Meals' as part of this law (44).

LONDON: Junk food adverts are to be banned from London's tube and bus network by Sadiq Khan, the city's mayor, in an attempt to curb an epidemic of childhood obesity. Ads that promote foods and drinks that are high in salt, fat and sugar will no longer be accepted on tubes, buses and overground trains that are part of the Transport for London (TfL) network – nor in stations. The ban is currently out to consultation.

QUEBEC (CANADA): In Quebec, most citizens speak French and it is the only province in Canada, where children below 13 years old are protected under the Consumer Protection Act since 1980(42). In Québec, the Consumer Protection Act prohibits commercial advertising directed at children less than 13 years of age through all media. To determine whether or not an advertisement is directed at persons under thirteen years of age, account must be taken of the context of its presentation, and in particular of: a) the nature and intended purpose of the goods advertised; b) the manner of presenting such advertisement; and c) the time and place it is shown (43). Any stakeholder involved in a commercial process (from the request to create an advertisement to its distribution, including its design) may be accused of not complying with the legislation in force. Per indictment, that person is liable to: a fine ranging from $600 to $15,000 (in the case of a natural person); a fine ranging from $2,000 to $100,000 (in the case of a legal person).Notably, for the rest of Canada, child-directed food

marketing is self-regulated using the Canadian Children's Food and Beverage Advertising Initiative (CAI) by Advertising Standards Canada (ASC) through The Broadcast Code for Advertising to Children.

Good Practice Indicator - PROMO3: Effective policies are implemented by the government to ensure that unhealthy foods are not commercially promoted to or for children <u>in settings where children gather</u> (e.g. preschools, schools, sport and cultural events).

- The draft school health policy (2017) prohibits the promotion of unhealthy foods in schools and marketing of any foods and beverages around the schools, it recommends that the schools actively promotes healthy foods instead.
- Development of policies to reduce the impact on children of marketing of foods and nonalcoholic beverages high in saturated fats, trans fatty acids, free sugars, or salt , is a key indicator in the Kenya National Strategy for prevention and Control of NCDs' monitoring frame work.
- The World Health organization and the Ministry of Health are in the process of piloting tools for profiling foods that are marketed for children. The tool will be useful in making decisions on the foods whose marketing should be restricted.

<u>**Evidence of policy action on PROMO3:**</u>
The **Draft National School Health Policy (2017)** includes a thematic area on school meals highlighting that schools shall ensure that no unhealthy foods are allowed or promoted in school, and that schools shall actively promote healthy foods. The policy also prohibits marketing of any foods and beverages within and around schools. It is important to note that this policy document is still under development by the government and other stakeholders.

<u>**International best practice examples (benchmarks) for PROMO3:**</u>

<u>CHILE</u>: In 2012, the Chilean government approved a Law of Nutritional Composition of Food and Advertising (Ley 20, 606)(32). In June 2015, the Chilean authority approved the regulatory norms required for the law's implementation (Diario Oficial No 41.193). The regulatory norms define limits for calories, saturated fat, sugar and sodium content considered "high" in foods and beverages. The regulatory norms define limits for calories (275 calories/100g or 70 calories/100mL), saturated fat (4g/100g or 3g/100mL), sugar (10g/100g or 5g/100mL) and sodium (400mg/100g or 100mg/100mL) content considered "high" in foods and beverages. The law restricts advertising directed to children under the age of 14 of foods in the "high in" category on school grounds, including preschools, primary and secondary schools. Promotional strategies and incentives, such as cartoons, animations, and toys that could attract the attention of children are included in the ban. The law is scheduled to take effect 1 July 2016 (21).

<u>SPAIN</u>: In 2011 the Spanish Parliament approved a Law on Nutrition and Food Safety (Ley 17/2011), which stated that kindergartens and schools should be free from all advertising. Criteria for the authorisation of food promotion campaigns, nutritional education and promotion of sports or physical activity campaigns were developed jointly by the Spanish Agency for Consumer Affairs, Food Safety and Nutrition (AECOSAN) and the Regional Health Authorities and implemented in July 2015. AECOSAN and the Spanish Regional Education and Health Administrations monitor the enforcement of the law (41).

<u>URUGUAY</u>: In September 2013, the government of Uruguay adopted Law No 19.140 "Alimentación saludable en los centros de enseñanza" (Healthy foods in schools)(50). The law prohibits the advertising and marketing of foods and drinks that don't meet the nutrition standards [referenced in Article 3 of the law, and outlined in school nutrition recommendations published by the Ministry of Health in 2014]. Advertising in all forms is prohibited, including posters, billboards, and use of

logos/brands on school supplies, sponsorship, and distribution of prizes, free samples on school premises and the display and visibility of food. The implementation of the law started in 2015 (41)

HUNGARY: Based on Section 8 of Act XLVIII on Basic Requirements and Certain Restrictions of Commercial Advertising Activities (2008), Hungary prohibits all advertising directed at children under 18 in child welfare and child protection institutes, kindergartens, elementary schools and their dormitories. Health promotion and prevention activities in schools may only involve external organizations and consultants who are recommended by the National Institute for Health Development according to Section 128(7) of the Ministerial Decree 20/2012 (VIII.31.) on the Operation of Public Education Institutions and the Use of Names of Public Education Institutions (41).

Good Practice Indicator - PROMO4: Effective policies are implemented by the government to restrict the marketing of breast milk substitutes.

Evidence of policy action for PROMO4:

The Breast Milk Substitutes Act (2012) restricts the advertisement, marketing, promotion, labelling of packaging, educational and information material related to breast milk substitutes in Kenya. A national multi-disciplinary Maternal Infant and Young Child Committee was established in 2014, with the main responsibility of monitoring the implementation of the act citing the need for controlled marketing of breast milk substitutes to/for infants 0 – 6 months, and rather, the promotion of exclusive breastfeeding when feasible.

- Although the Breast Milk Substitutes Act 2012 and a national Maternal Infant and Young Child Committee are in place, the implementation and monitoring has not progressed effectively due to delays in developing the Breast Milk Substitutes Act Regulations, which are currently in draft form. The draft regulations are currently ready for submission to the Parliament to be discussed and enacted into law. The development of the BMS implementation framework which will guide the implementation and monitoring of the BMS act is also underway.

The Advertising Standards Body of Kenya - Code of Advertising Practice and Direct Marketing 2003 (Parts I and II) also restricts the advertisement and marketing of breast milk substitutes, and baby teats, which are only allowed with approval from a health professional and with information that is scientific and factual.

In the most recent 2018 WHO status report on the implementation of the International Code of Marketing of Breast-milk Substitutes, Kenya was 1 of only 35 countries to be classified as having full provisions of the International Code of Marketing of Breast-milk Substitutes (and subsequent resolutions adopted by the World Health Assembly) covered in law (18)

International best practice examples (benchmarks) for PROMO4:

SOME COUNTRIES: As of 2018, a total of 35 countries have enacted legislation or adopted regulations, decrees or other legally binding measures encompassing all or nearly all provisions of International Code of Marketing of Breast-milk Substitutes and subsequent resolutions adopted by the World Health Assembly (18).

DOMAIN 4 - FOOD PRICES (PRICES)

Food pricing policies (e.g., taxes and subsidies) are aligned with health outcomes by helping to make healthy eating choices easier and cheaper.

Good Practice Indicator - PRICES1: Taxes or levies on healthy foods are minimised to encourage healthy food choices where possible (e.g. low or no sales tax, excise, value-added or import duties on fruit and vegetables).

Evidence of policy action for PRICES1:
The Budget Statement for the Fiscal Year 2017/2018 proposed that ordinary bread and maize flour be VAT exempt (zero rated), to make these commodities (considered as essential foods) affordable to poorer families, ensuring target populations have adequate food, **rather than** to promote healthy eating/food choices.

The Government intervenes in maize markets (as well as others such as wheat) through trade and marketing policies, given that maize is a staple food/core to energy intake (accounting for 36% of energy intake), and that maize prices are a crucial social and political issue. Tariffs are imposed on maize imports as a way to regulate the market/prices, **rather than** as a direct mechanism to promote maize consumption as a 'healthy' food (12, 13, 14).

International best practice examples (benchmarks) for PRICES 1:
AUSTRALIA: Goods and services tax (GST) exemption exists for basic foods (including fresh fruits and vegetables)(51).

TONGA: In 2013, as part of a broader package of fiscal measures, import duties were lowered from 20% to 5% for imported fresh, tinned or frozen fish in order to increase affordability and promote healthier diets (52).

POLAND: In Poland, the basic rate of tax on goods and services is 22%, while the rate is lower (7%) for goods related to farming and forestry and even lower (3%) for unprocessed and minimally processed food products (53).

FIJI: To promote fruit and vegetable consumption, Fiji has removed the excise duty on imported fruits, vegetables and legumes. It has also decreased the import tax for most varieties from the original 32% to 5% (exceptions: 32% remains on tomatoes, cucumbers, potatoes, squash, pumpkin and 15% remains on coconuts, pineapples, guavas, mangosteens) and removed it for garlic and onions (52).

Good Practice Indicator - PRICES2: Taxes or levies on unhealthy foods (e.g. sugar-sweetened beverages, foods high in nutrients of concern) are in place and increase the retail prices of these foods by at least 10% to discourage unhealthy food choices where possible, and these taxes are reinvested to improve population health.

Evidence of policy action for PRICES2:
Policy regulation on charging excise tax/ duty on the local production and sale of sugar is in the initial stages of development by the Ministry of Health - Unit of Non-Communicable Diseases and the Unit of Human Nutrition, and the Kenya Revenue Authority among others. The proposed policy regulation requires that an excise tax is levied on sugar production and sales in the country. The health risks associated with consumption of sugar is one of the references for the proposed regulations.

An Excise Duty Act imposes excise duty rates on various unhealthy food items since they are regarded as luxury foods. These include fruit juices and other non-alcoholic beverages (whether or not containing added sugar or other sweetening at Kshs 5 to 10 per litre) and food supplements at 10%.

International best practice examples (benchmarks) for PRICES2:

MEXICO: In December 2013, the Mexican legislature passed two new taxes as part of the national strategy for the prevention of overweight, obesity and diabetes. An excise duty of 1 peso ($0.80) per litre applies to sugary drinks. Sugary drinks are defined under the new law as all drinks with added sugar, excluding milks or yoghurts. This is expected to increase the price of sugary drinks by around 10%. An ad valorem excise duty of 8% applies to foods with high caloric density, defined as equal to or more than 275 calories per 100 grams. The food product categories that are affected by the tax include chips and snacks; confectionary; chocolate and cacao based products; puddings; peanut and hazelnut butters. The taxes entered into force on 1 January 2014. The aim is for the revenue of taxes to be reinvested in population health, namely providing safe drinking water in schools, but there is no evidence (yet) that this is the case as the taxes are not earmarked (52, 54).

HUNGARY: A "public health tax" adopted in 2012 is applied on the salt, sugar and caffeine content of various categories of ready-to-eat foods, including soft drinks (both sugar- and artificially-sweetened), energy drinks and pre-packaged sugar-sweetened products. The tax is applied at varying rates. Soft drinks, for example, are taxed at $0.24 per litre and other sweetened products at $0.47 per litre. The tax also applies to products high in salt, including salty snacks with >1g salt per 100g, condiments with >5g salt per 100g and flavourings >15g salt per 100g (52, 55).

FRENCH POLYNESIA: Various food and beverage taxes have been in place since 2002 to discourage consumption and raise revenue, e.g. domestic excise duty on sweetened drinks and beer; import tax on sweetened drinks, beer and confectionery; tax on ice cream. Between 2002 and 2006, tax revenue went to a preventive health fund; from 2006, 80% has been allocated to the general budget and earmarked for health. The tax is 40 CFP (around $0.44) per litre on domestically-produced sweet drinks, and 60 CFP (around $0.68) per litre on imported sweet drinks (52).

ST HELENA: In effect since 27 May 2014, a £0.75 per litre excise duty (about $1.14) is applied to high-sugar carbonated drinks in St Helena (Customs and Excise Ordinance Chapter 145, Section 5). High sugar carbonated drinks are defined as drinks containing ≥15 grams of sugar per litre (52).

UK: In April 2018, the UK Government's Soft Drinks Industry Levy came into effect. The Levy applies to any pre-packaged soft drink with added sugar, containing at least 5g of sugar per 100ML of prepared drink (56). Drinks fall into two bands: one for total sugar content above 5g per 100ml (to be taxed at 18 pence per L), and a second, higher band for the most sugary drinks with more than 8g per 100ml (to be taxed at 24 pence per L). Milk-based drinks, milk substitute drinks, pure fruit juices, or any other drinks with no added sugar, alcohol substitute drinks and soft drinks of a specified description which are for use for medicinal or other specified purposes are exempt from the levy. The levy applies to soft drinks produced and packaged in the UK and soft drinks imported into the UK. The measure will raise an estimated £520 million a year, and will be spent on doubling funding for sport in primary schools. Secondary schools will meanwhile be encouraged to offer more sport as part of longer school days. Pure fruit juices and milk-based drinks will be excluded, as well as small producers.

BAHRAIN: The Kingdom of Bahrein has introduced an excise tax which went into effect on 30 December 2017. The excise tax rate imposed by the law is a 100% tax rate on energy drinks and a 50% tax rate on soft drinks. Soft drinks are defined as any aerated beverage except unflavoured aerated water and include any concentrates, powder, gel or extracts intended to be made into an aerated beverage. Any person intending to import or produce the excisable good are required to register for the tax.

Good Practice Indicator - PRICES3: The intent of existing subsidies on foods, including infrastructure funding support (e.g. research and development, supporting markets or transport systems), is to favour healthy rather than unhealthy foods.

Evidence of policy action for PRICES3:

The Budget Statement for the Fiscal Year 2017/2018 indicated that national government disbursed a total of Kshs 7.3 billion, while county governments provided Kshs 2 billion, to cater for a doubling of food rations, cash transfers, and other measures ensuring target populations have adequate food, **rather than** to specifically promote healthy food.

International best practice examples (benchmarks) for PRICES3:

***CANADA*:** Launched on April 1, 2011, Nutrition North Canada (NNC) is a Government of Canada subsidy program that helps provide Northerners in isolated communities with improved access to perishable, nutritious food. NNC provides a retail-based subsidy that enables local retailers and registered suppliers to access and lower the cost of perishable healthy foods like meat, fish, eggs, milk and bread, as well as fruits and vegetables, all of which must be transported by air to these isolated communities. NNC has two subsidy levels: level 1 which is the higher subsidy, is granted to foods that are deemed the most perishable and most nutritious and level 2, the lower subsidy, is applied to other staple food items.

SINGAPORE: The government, through the Health Promotion Board (HPB) increases the availability and use of healthier ingredients through the "Healthier Ingredient Scheme" (formerly part of the "Healthier Hawker" programme, launched in 2011), which provides in the first instance transitional support to oil manufacturers and importers to help them increase the sale of healthier oils to the food service industry (59). The Healthier Ingredient Subsidy Scheme offers a subsidy to suppliers stocking healthier items. Cooking oil is the first ingredient under the scheme, which subsidises oils with a saturated fat level of 35 per cent or lower.

Good Practice Indicator - PRICES4: The government ensures that food-related income support programmes are for healthy foods.

Evidence of policy action for PRICES4:

The Budget Statement for the Fiscal Year 2017/2018 indicated that there has been a doubling of food rations and cash transfers to families in need. To enable the country to maintain adequate food reserves and ensure productivity of the lands, the government allocated Kshs 1.3 billion for strategic grain reserves, and Kshs 0.1 billion for mechanisation of agriculture. **However**, these strategies are not specifically targeted towards supporting healthy foods.

Five Cash Transfer (CT) programmes are in place in Kenya supporting approximately 2 million people (11% of the absolute poor). These are: Cash Transfer for Orphans and Vulnerable Children (CT-OVC), the Older Persons Cash Transfer (OPCT), the Urban Food Subsidy Cash Transfer (UFS-CT), the Persons with Severe Disability Cash Transfer (PWSD-CT) and the Hunger Safety Net Program (HSNP). Most are managed by the Ministry of Labour, Social Security and Services (MLSSS), with the exception of the HSNP which is managed by the National Drought Management Authority (NDMA), with support from NGOs. Despite rising investments, CT programmes have remained fragmented and largely uncoordinated. It is important to note that many of these CT programmes are not directly food-related, and do not directly promote consumption of 'healthy foods' (15). Some counties have established their own CT programmes.

International best practice examples (benchmarks) for PRICES4:

UK: The British Healthy Start programme provides pregnant women and/or families with children under the age of four with weekly vouchers to spend on foods including milk, plain yoghurt, and fresh and frozen fruit and vegetables. Participants or their family must be receiving income support/jobseekers allowance or child tax credits. Pregnant women under the age of 18 can also apply. Full national implementation of the programme began in 2006 (52).

US: In 2012, the USDA piloted a "Healthy Incentives Pilot" as part of the Supplemental Nutrition Assistance Program (SNAP, formerly "food stamps"). Participants received an incentive of 30 cents per US$ spent on targeted fruit and vegetables (transferred back onto their SNAP card). The Pilot included 7500 individuals (52). In New York City and Philadelphia, "Health Bucks" are distributed to farmers markets. When customers use income support (e.g. Food Stamps) to purchase food at farmers markets, they receive one Health Buck worth 2USD for each 5USD spent, which can then be sued to purchase

fresh fruit and vegetable products at a farmers market (52). In Philadelphia, the programme has been expanded to other retail settings like supermarkets and corner stores.

US: In 2009, the U.S. Department of Agriculture's implemented revisions to the Special Supplemental Nutrition Program for Women, Infants, and Children (WIC) to improve the composition and quantities of WIC-provided foods from a health perspective. The revisions include: Increase the dollar amount for purchases of fruits and vegetables, expand whole-grain options, allow for yoghurt as a partial milk substitute, allow parents of older infants to buy fresh produce instead of jarred infant food and give states and local WIC agencies more flexibility in meeting the nutritional and cultural needs of WIC participants (52).

DOMAIN 5 - FOOD PROVISION (PROV)

The government ensures that there are healthy food service policies implemented in government-funded settings to ensure that food provision encourages healthy food choices.

Good Practice Indicator - PROV1: The government ensures that there are clear, consistent policies (including nutrition standards) implemented in schools and early childhood education services for food service activities (canteens, food at events, fundraising, promotions, vending machines etc.) to provide and promote healthy food choices.

Evidence of policy action for PROV1:
Kenya has an established school feeding programme, employed as a way to distribute food to school attending children. The programme consists of a lunch combined with health education and, when clinically indicated, vitamin or iron supplements. Positive impact owing to the programme has been observed on nutritional status of children and anemia (16). Early childhood education centres are currently not covered by this national programme; however, some counties are planning to establish feeding programmes for their centres.

The National Nutrition Action Plan (NNAP) (2012 - 2017), strategic objective 7 focuses on improved nutrition in schools, public and private institutions. Developing nutrition guidelines for schools and other institutions and integrating nutrition education in the school curriculum are stipulated as among the key priority areas.

A school health policy (in draft form) is under development by the government and other stakeholders. The Draft School Health Policy (2017) highlights nutrition among the key thematic areas, calling for the provision of diverse, safe, and nutritious food of good quality and in adequate quantities in schools, as a key strategy to optimise nutrition of children.

- School meals are also highlighted as a key strategy to optimising nutrition for children in schools, within which schools are required to have kitchens that provide nutritious and affordable meals to children, to actively promote healthy foods and to prohibit the promotion of unhealthy foods in schools.
- Schools shall ensure that unhealthy foods are not allowed or promoted in school, and that schools shall actively promote healthy foods.
- The policy also prohibits marketing of any foods and beverages within and around schools.
- Development of school gardens and keeping of small livestock are recommended as a way of supplementing produce for school meals.

The School Nutrition and Meals Strategy for Kenya (2016), provides a framework for the implementation of school meals in Kenya. Increasing the intake and awareness of adequate, culturally appropriate nutritious meals amongst school age children (in schools and ECD centres), is one of the key objectives.

- This strategy sets requirements (food composition) for a standard school meal menu, which provides about one-third of the macro and micronutrient needs of school children.
- It emphasises the need to ensure dietary diversity and nutrition standards, and consider local needs, traditions, and food habits in the provision of school meals.
- It emphasises the linkages between school meals, and health and nutrition education for school-aged children to make nutritious informed food choices through the lifecycle.
- The school meals strategy is being implemented in some schools, and particularly public schools in food insecure settings (arid and semi-arid areas).

School Curriculum reform is underway in Kenya, and food and nutrition content has been included in the revised curriculum to facilitate nutrition education among school going children/ adolescents, as stipulated in the National Nutrition Action Plan.

The National Food and Nutrition Security Policy (2011) establishes standards and regulations for school meals programmes, covering food storage, preparation, handling, and quantity of food served to students. It calls for improved quality and expansion of school meal programmes to

include pre-schools and boarding schools in collaboration with local communities. It discourages the selling of food in and around schools by unsolicited parties. It further calls for the provision of appropriate training on preparation and service of quality foods to food handlers in school kitchens.

International best practice examples (benchmarks) for PROV1:

CHILE: In 2012, the Chilean government approved a Law of Nutritional Composition of Food and Advertising (Ley 20, 606)(32). In June 2015, the Chilean authority approved the regulatory norms required for the law's implementation (Diario Oficial No 41.193). The regulatory norms define limits for calories, saturated fat, sugar and sodium content considered "high" in foods and beverages. The regulatory norms define limits for calories (275 calories/100g or 70 calories/100mL), saturated fat (4g/100g or 3g/100mL), sugar (10g/100g or 5g/100mL) and sodium (400mg/100g or 100mg/100mL) content considered "high" in foods and beverages. The law prohibits the sale of foods in the "high in" category in schools. These were scheduled to take effect 1 July 2016 (62).

FINLAND: In 2008, the National Nutrition Council approved nutrition recommendations for school meals. These include food and nutrient recommendations for salt, fibre, fat, starch, fat and salt maximums for meat and processed meat, and drinks. There are also criteria for snacks provided in schools (62).

AUSTRALIA: There are no national mandatory standards. However, six states and territories have implemented mandatory standards, which are either based on the national voluntary guidelines or nutrient and food criteria defined by the state: Australian Capital Territory (2015), New South Wales (2011), Northern Territory (2009), Queensland (2007), South Australia (2008), and Western Australia (2014). All of these states and territories identify 'red category' foods, which are either completely banned in schools or heavily restricted (e.g. offered no more than one or two times per term)(62). The New South Wales (NSW) policy for school canteens prohibits availability of red foods, high in saturated fats, sugars, or sodium. Foods provided in school canteens should be at least 50% green foods to ensure that canteens do not increase the number of "amber" foods. Green foods include low-fat carbohydrates, fruits and vegetables, and lean meat as well as small portions of pure fruit juice. Also Queensland's Smart Choices school nutrition standards ensure that "red" foods and drinks are eliminated across the whole school environment.

MAURITIUS*:* In 2009, a regulation was passed banning soft drinks, including diet soft drinks, and unhealthy snacks from canteens of pre-elementary, elementary and secondary schools (62).

UK*:* England, Scotland, Wales and Northern Ireland have mandatory nutritional standards for school food, which also apply to food provided in schools other than school lunches. These standards apply to most state schools (with the exception of around 4,000 academies established between September 2010 and June 2014, which are exempt) and restrict foods high in fat, salt and sugar, as well as low quality reformed or reconstituted foods (62).

BRAZIL*:* The national school feeding programme (63) places great emphasis on the availability of fresh, traditional and minimally processed foods. It mandates a weekly minimum of fruits and vegetables regulates sodium content and restricts the availability of sweets in school meals. A school food procurement law (64), approved in 2001, limits the amount of processed foods purchased by schools to 30%, and bans the procurement of drinks with low nutritional value, such as sugary drinks. The law requires schools to buy locally grown or manufactured products, supporting small farmers and stimulating the local economy. Resolution no 38 (16 July 2009) sets food- and nutrition-based standards for the foods available in the national school meal programme (Law 11.947/2009). Article 17 prohibits drinks of low nutritional value (e.g. soda), canned meats, confectionary and processed foods with a sodium and saturated fat content higher than a specified threshold.

COSTA RICA: Executive Decree No 36910-MEP-S (2012) of the Costa Rican Ministries of Health and Education sets restrictions on products sold to students in elementary and high schools, including food with high levels of fats, sugars and salt, such as chips cookies candy and carbonated sodas. Schools are only permitted to sell food and beverages that meet specific nutritional criteria. The restrictions were upheld by the Constitutional Court in 2012 following a challenge by the food industry(62).

***HUNGARY*:** Since 2012, food and beverages subject to the public health product tax may not be sold on school premises or at events organized for school children, including out of school events based on the Ministerial Decree 20/2012 (VIII.31) on the Operation of Public Education Institutions and the Use of Names of Public Education Institutions. Section 130(2) of the Decree requires the head of the educational institution to consult the school health service prior to entering into agreements with vending machine operators or food vending businesses. The school health service verifies whether the products to be sold meet the nutritional guidelines set by the National Institute of Pharmacy and Nutrition. Products that do not comply are prohibited (62).

URUGUAY: In September 2013 the government of Uruguay adopted Law No 19.140 on "healthy eating in schools". It mandated the Ministry of Health to develop standards for food available in canteens and kiosks in schools, prohibited advertising for these same foods and restricted the availability of salt shakers. The school food standards were elaborated in March 2014 in two further documents: Regulatory Decree 60/014 and the National Plan of Health Promoting Schools. The standards aimed to promote foods with natural nutritional value with a minimum degree of processing and to limit the intake of free sugars, saturated fat, trans fat and sodium. Limits are set per 100g of food, 100ml for drinks and also per 50g portion. Prohibited foods include sugary beverages and energy drinks, confectionery, salty snacks, cakes and chocolate. The school food standards and restrictions on advertising began to be implemented in public schools in 2015 and are being monitored for compliance (62).

Good Practice Indicator - PROV2: The government ensures that there are clear, consistent policies in other public sector settings for food service activities (canteens, hospitals, clinics, food at events, fundraising, promotions, vending machines, public procurement standards etc.) to provide and promote healthy food choices.

Evidence of policy action for PROV2:

The National Nutrition Action Plan (NNAP) (2012 - 2017), strategic objective 7 focuses on improved nutrition in schools, public, and private institutions. However, there is no evidence of implementation in other public and private institutions besides school settings.

International best practice examples (benchmarks) for PROV2:

LATVIA: In 2012, the government set salt levels for all foods served in hospitals and long-term social care institutions. Levels may not exceed 1.25g of salt per 100g of food product; fish products may contain up to 1.5g of salt per 100g of product (62).

BERMUDA: In 2008, the Government Vending Machine Policy was implemented in government offices and facilities to ensure access to healthy snacks and beverages for staff. The policy requires that all food and beverages in vending machines on government premises meet specific criteria based on levels of total fat, saturated fat, *trans* fat, sodium and sugar. Criteria exclude nuts & 100% fruit juices (62).

NEW YORK: New York City's Food Standards (enacted with Executive Order 122 of 2008) set nutritional standards for all food purchased or served by city agencies, which applies to prisons, hospitals and senior care centres. The Standards include: maximum and minimum levels of nutrients per serving; standards on specific food items (e.g. only no-fat or 1% milk); portion size requirements; the requirement that water be offered with food; a prohibition on the deep-frying of foods; and daily calorie and nutrient targets, including population-specific guidelines (e.g. children, seniors)(62, 68). As of 2015, 11 city agencies are subject to the NYC Food Standards, serving and selling almost 250 million meals a year. The Food Policy Coordinator has the

responsibility of ensuring adherence with the Food Standards. Self-reported compliance with the standard is 96%.

WALES: Vending machines dispensing crisps, chocolate and sugary drinks are prohibited in National Health Service hospitals in Wales. The Welsh government issued a guidance defining what is allowed and not allowed, and has liaised with major vending providers to find ways to introduce healthier food and drink options (Health Promoting Hospital Vending Directions and Guide 2008).

UK: The UK Government Buying Standard for Food and Catering Services (GBSF of 2014, updated March 2015) by the Department of Environment, Food and Rural Affairs, sets out standards for the public sector when buying food and catering services. It is supported by the Plan for Public Procurement: Food and Catering Services (2014), which includes a toolkit consistent of the mandatory GBSF, a balanced scorecard, an e-marketplace, case studies and access to centralised framework contacts in order to improve and facilitate procurement in the public sector. The nutrition requirements have to be followed by schools, hospitals, care homes, communities and the armed forces. To improve diets, the GBSF sets maximum levels for sugar in cereals and generally for saturated fat and salt, in addition to minimum content of fibre in cereals and fruit in desserts. Meal deals have to include vegetables and fruit as dessert and menus fish on a regular basis (59).

Good Practice Indicator - PROV3: The Government ensures that there are good support and training systems to help schools and other public sector organisations and their caterers meet the healthy food service policies and guidelines.

Evidence of policy action for PROV3:
The National Food and Nutrition Security Policy (2011) calls for the provision of appropriate training on preparation and service of quality foods to food handlers in school kitchens. We identified no further evidence of implementation.

International best practice examples (benchmarks) for PROV3:
AUSTRALIA: The Healthy Eating Advisory Service supports settings such as childcare centres, schools, workplaces, health services, food outlets, parks and sporting centres to provide healthy foods and drinks to the public in line with Victorian Government policies and guidelines. The Healthy Eating Advisory Service is delivered by experienced nutritionists and dieticians at Nutrition Australia Victorian Division. The support includes training cooks, chefs, foods service and other key staff, discovering healthier recipes, food ideas and other helpful resources to provide healthier menus and products (69).

JAPAN: In Japanese, “Shoku” means diet and “iku” means growth and education. In 2005, Basic Law on Shokuiku was enacted and it was the first law that regulates one’s diets and eating habits. It involved Cabinet Office as the leading office to plan, formulate and coordinate Shokuiku policy and strategy, in collaboration with Ministry of Health, Labour and Welfare, Ministry of Education, Culture, Sports, Science and Technology (MEXT) and Ministry of Agriculture, Forestry and Fisheries. The laws included several concepts, which are promotion of Shokuiku at home, schools or nursery schools and promotion of interaction between farm producers and consumers(70). Dietitian and registered dietitian are playing important roles to implement Shokuiku programs by providing dietary guidance in various setting. In Japan, at least one dietitian should be assigned at the facility with mass food service over 100 meals/time or over 250 meals/ day, whereas at least one registered dietitian needed when it is over 500 meals/time or 1500 meals/day. In specific setting such as school, the Ministry of Education, Culture, Sports, Science and Technology established the Diet and Nutrition Teacher System in 2007. Diet and Nutrition Teachers are responsible to supervise school lunch programs, formulate menus and ensure hygiene standards in public elementary schools and junior high schools in accordance with the needs of local communities. They also deal with dietary education issues in collaboration with nutrition experts such as registered dietitian and dietitian(71). Under the revised School Lunch Act 2008, it included School Lunch Practice Standard which stipulates proper school lunch including reference

intake values of energy and each nutrient as per age groups (72). Moreover, it outlined costs of facilities and manpower (e.g. cooks) to be covered by municipalities and guardians only cover the cost of ingredients which amounting an estimate of 4000 yen/month/student for school lunch program (73).

DOMAIN 6 - FOOD IN RETAIL (RETAIL)

The government has the power to implement policies and programmes to support the availability of healthy foods and limit the availability of unhealthy foods in communities (outlet density and locations) and in-store (product placement).

Good Practice Indicator - RETAIL1: Zoning laws and policies are robust enough and are being used, where needed, by local governments to place limits on the density or placement of quick serve restaurants or other outlets selling mainly unhealthy foods in communities, and to encourage the availability of outlets selling healthy options such as fresh fruit and vegetables.

Evidence of policy action for RETAIL1:
None found.

International best practice examples (benchmarks) for RETAIL1:
SOUTH KOREA: In 2010 the Special Act on Children's Dietary Life Safety Management established the creation of 'Green Food Zones' around schools, banning the sale of foods (fast food and soda) deemed unhealthy by the Food and Drug Administration of Korea within 200 metres of schools (45, 76). In 2016, Green Food Zones existed at over 10000 schools.

DUBLIN (IRELAND)**:** Fast-food takeaways will be banned from opening within 250 metres of schools, Dublin city councillors have ruled. The measure to enforce "no-fry zones" will be included in a draft version of the council's six-year development plan. City planners will be obliged to refuse planning permission to fast food businesses if the move is formally adopted after public consultation(77).

US: In Detroit, the zoning code prohibits the building of fast food restaurants within 500 ft. of all elementary, junior and senior high schools(13)

UK: Around 15 local authorities have developed "supplementary planning documents" on the development of hot food takeaways. The policies typically exclude hot food takeaways from a 400m zone around the target location (e.g. primary schools). For example, Barking and Dagenham's Local Borough Council, London, adopted a policy in 2010 restricting the clustering of hot food takeaways and banning them entirely from 400m exclusion zones around schools. In 2009, the Local Borough Council of Waltham Forest, London developed a planning policy in 2009 restricting the development of hot food takeaways in local centres, and excluding them completely from areas within 10min walks from schools, parks or other youth centres. St Helens Council adopted a planning document in 2011 and Halton in 2012 (13).

US: In February 2014 the US Congress formally established the Healthy Food Financing Initiative (following a three year pilot) which provides grants to states to provide financial and/or other types of assistance to attract healthier retail outlets to underserved areas. The pilot distributed over 140 million USD in grants to states to provide financial and other types of assistance to attract healthier retail outlets in underserved areas. To date, 23 US states have implemented financing initiatives (13). For example, the New Jersey Food Access Initiative provides affordable loans and grants for costs associated with building new supermarkets, expanding existing facilities, and purchasing and installing new equipment for supermarkets offering a full selection of unprepared, unprocessed, healthy foods in under-served areas; the initiative targets both for-profit and not-for-profit organisations and food cooperatives.

NEW YORK: The 'Green Cart Permit' was developed with reduced restrictions on zoning requirements to increase the availability of fresh fruits and vegetables in designated, underserved neighbourhoods(13). In 2008 New York City made 1000 licences for green carts available to street vendors who exclusively sell fresh fruit and vegetables in neighbourhoods with limited access to healthy foods(13). In addition, in 2009, New York City established the food retail expansion to support health program of New York City (FRESH). Under the programme, financial and zoning incentives are offered to promote neighbourhood grocery stores offering fresh meat, fruit and

vegetables in under-served communities. The financial benefits consist of an exemption or reduction of certain taxes. The zoning incentives consist of providing additional floor area in mixed buildings, reducing the amount of required parking, and permitting larger grocery stores in light manufacturing districts.

SCOTLAND: In 2004, a small group of suppliers and retailers in Scotland established a pilot project called Healthy Living Neighbourhood Shops to increase the availability of healthier food options throughout Scotland, in both deprived and affluent areas, where little or no option existed to buy. The programme received funding from the Scottish Executive and worked closely with the Scottish Grocers' Federation, which represents convenience stores throughout Scotland. Through a number of different trials, the programme established clear criteria for increasing sales and also developed bespoke equipment/point of sale (POS) materials which were given to participating retailers free of charge. This has led to around 600 convenience stores across Scotland improving their range, quality and stock of fresh fruit and vegetables and other healthier eating products (78).

Good Practice Indicator - RETAIL2: The Government ensures existing support systems are in place to encourage food stores and food service outlets to promote the availability of healthy foods and to limit the promotion and availability of unhealthy foods.

Evidence of policy action for RETAIL2:

The National Food and Nutrition Security Policy (2011) addresses micronutrient deficiencies by developing standards and supporting high quality commercial micronutrient fortification activities. It also promotes guidelines developed for the distribution and sale of over-the-counter vitamin and mineral supplements. The Ministry of Health - Unit of Nutrition coordinates the distribution/provision of micronutrients to vulnerable populations/groups. However, these initiatives are not specifically targeted at promoting the availability of healthy foods/limiting the availability of unhealthy foods associated with NCDs.

International best practice examples (benchmarks) for RETAIL2:

US: The Special Supplemental Nutrition Program for Women, Infants, and Children (WIC) requires WIC authorised stores to stock certain healthier products (e.g. wholegrain bread) (52).

DOMAIN 7 - FOOD TRADE AND INVESTMENT (TRADE)

The government ensures that trade and investment agreements protect the capacity of government to make decisions, favour healthy food environments, and are linked with domestic health and agricultural policies in ways that are consistent with health objectives, and do not promote unhealthy food environments.

Good Practice Indicator - TRADE1: The Government undertakes risk impact assessments before and during the negotiation of trade and investment agreements, to identify, evaluate and minimise the direct and indirect negative impacts of such agreements on population nutrition and health.

Evidence of policy action for TRADE1:
None found.

International best practice examples (benchmarks) for TRADE1:
US/EU: It is mandatory in the US and countries of the EU to undertake Environmental Impact Assessments for all new trade agreements. These assessments sometimes incorporate Health Impact Assessments (84).

Good Practice Indicator - TRADE2: The government adopts measures to manage investment and protect their regulatory capacity with respect to public health nutrition.

Evidence of policy action for TRADE2:
The National Food and Nutrition Security Policy (2011) states that to ensure adequate quantity and quality of food accessible to all Kenyans for a diverse and healthy diet, among other means, that it is important to promote food trade to ensure a predictable supply of commercially available food.

- Trade in agricultural commodities, without displacing local production, is a major determinant of national food security granted rapid population increase, declining per capita production and self-sufficiency, urbanisation and changing eating habits.
- More predictable and transparent involvement of state in markets, particularly in changes in import tariffs and purchase or sale prices, will reduce uncertainty and ensure a predictable supply of food imports and reduce costs of food.
- Official and informal cross-border trade with the neighbouring countries is common. Therefore, enhanced trade in food products among the EAC and COMESA member States will ensure regional food self-sufficiency, provided food safety is ensured.
- Following Government actions to improve regional food trade, the major challenges for Kenyan farmers is to reduce the cost of their production and develop marketing arrangements to make farm products competitive through facilitating the competitiveness of Kenya's agricultural sector; fostering regional trade by adhering to EAC and COMESA trade policies; contributing towards harmonisation of regional standards to provide a level playing field and fair trade practices for Kenyan farmers; and, controlling dumping of subsidised foods.
- The Policy further highlights that all international and regional treaties and conventions to which Kenya is a party related to agriculture, food, and nutrition security should be adapted to the Kenyan context, to ensure the achievement of food nutrition and security.

It is important to note that the focus above may be more heavily geared towards sustainable production and availability of food that is diversified, affordable, and helps meet basic nutrition requirements, rather than food/nutrition in relation to obesity and NCDs.

International best practice examples (benchmarks) for TRADE 2:
MANY COUNTRIES: Sanitary and phytosanitary (SPS) clauses in World Trade Organization (WTO) agreements. However, this usually does not apply to public health nutrition.

CANADA/EU: CETA makes clear that the EU and Canada preserve their right to regulation to achieve legitimate policy objectives, such as public health, safety, environment, public morals,

social or consumer protection and the promotion and protection of cultural diversity (Art. 8.9). CETA includes a clear definition for what constitutes “indirect expropriation” to avoid claims against legitimate public policy measures. This includes a clause that the sole fact that a measure increase costs for investors cannot give rise in itself to a finding of expropriation (Annex 8.12 and Annex 8-A).

GHANA: Ghana has set standards to limit the level of fats in beef, pork, mutton and poultry in response to rising imports of low quality meat following liberalization of trade. The relevant standards establish maximum percentage fat content for de-boned carcasses/cuts for beef (<25%), pork (<25%) and mutton (<25% or <30% where back fat is not removed), and maximum percentage fat content for dressed poultry and/or poultry parts (<15%)(85).

Food Environment Policy Index: INFRASTRUCTURE SUPPORT Domains

DOMAIN 8 - LEADERSHIP (LEAD)

The political leadership ensures that there is strong support for the vision, planning, communication, implementation and evaluation of policies and actions to create healthy food environments, improve population nutrition, and reduce diet-related inequalities.

Good Practice Indicator - LEAD1: There is strong, visible, political support (at the Head of Government/Cabinet level) for improving food environments, population nutrition, diet-related NCDs and their related inequalities.

Evidence of policy action for LEAD1:
The Kenya National Strategy for the Prevention and Control of Non-Communicable Diseases (2015-2020), highlights that in Kenya, NCDs account for more than 50% of total hospital admissions and over 55% of hospital deaths, and yet, NCD prevention and control receives inadequate attention. This document, developed by the government in collaboration with key stakeholders, provides a framework for reducing preventable NCD-related morbidity and mortality. Promotion of healthy lifestyles including healthy diets is highlighted as a key strategic objective towards reducing NCDs disease risks.

The Ministry of Health's Unit of Nutrition has established a department on Healthy Diets and Physical Activity, which works closely with the Division of Non-Communicable Diseases to implement the NCD strategy including promotion of healthy diets for improved prevention and management of diet related NCDs. Consequently, the National Guideline for Healthy Diets and Physical Activity (2017), developed by government in collaboration with other stakeholders, provides guidance and key messages on healthy diets and physical activities for the general population, as a key strategy to address NCDs mortality and risks among other nutrition related diseases. This step serves as a starting point for the development Kenya's Food-Based Dietary Guidelines.

- These documents have support from the highest levels of office at the Ministry of Health, including but not limited to the Cabinet Secretary for Health, Director of Medical Services, and Head of the Nutrition Department.

In the Draft School Health Policy document, the prevention and management of NCDs in schools is also a key thematic area. Promotion of healthy lifestyles including healthy diets and physical activity in schools are highlighted as key strategies to reducing the incidence of NCDs among children and adolescents.

The Kenya Health Sector Strategic and Investment Plan (KHSSP) 2013-2017, provides the health sector and related sectors with medium-term focus, objectives, and priorities to enable them to move towards attainment of the Kenya Health Policy Directions. It follows on from the 2nd National Health Sector Strategic Plan (NHSSP II), which had the overall goal to reduce inequalities in health care services and reverse the downward trend in health-related outcome indicators. Strategic objective 2 within the KHSSP is to halt and reverse the rising burden of NCDs by providing prevention activities; implementing interventions directly addressing marginalised and indigent populations affected by NCDs; integrating health service provision tools, mechanisms and processes for NCDs; and, establishing screening programmes in health facilities for major NCDs. Among major NCDs targeted are: mental health, type 2 diabetes, cardiovascular diseases, chronic obstructive airway conditions, blood disorders focusing on Sickle cell conditions, and cancers, which contribute the most to the NCD burden.

- The Statistical Review of Progress towards the Mid-Term Targets of the Kenya Health Sector Strategic Plan 2014-2018 reviewed the evidence for progress made during the first half (2014-mid 2016) of the implementation of the Kenya Health Sector Strategic and Investment Plan (KHSSP), using all available data sources, and paying special attention to county progress. Data from the STEPS 2015 showed that the prevalence of overweight and obesity had increased dramatically in the last decade, especially among women (38% overweight or obese), as observed in all counties with some variations in rates. Raised blood pressure

(>=140/90 mm Hg) affected almost a quarter of adults (23%), with few diagnosed and treated, and only 4% of those on treatment had their blood pressure controlled. Diabetes prevalence was 1.9% among persons 15-69 years but only 41% had been diagnosed and effective treatment. Cervical cancer screening rates were low throughout Kenya with only one in 7 women 25-49 years (ever) screened. All these findings indicated that greater efforts were required to curb the rapid rise of NCDs and their risk factors.

One of the priority areas identified in The National Nutrition Action Plan (NNAP) (2012 – 2017) is the development of comprehensive strategies and guidelines for the prevention, management and control of diet related NCDs, as well as creating public awareness on the importance of prevention, management and control of diet related NCDs through a national Advocacy, Communication and Social Mobilisation (ACSM) strategy. One of the strategies employed to improve nutritional knowledge, attitudes, and practise is to mark nutrition days nationally and in all counties. Nutrition days include World Breastfeeding Week, African Food and Nutrition Security Day, Iodine Deficiency Disorders Day, and *Malezi Bora.*

The Kenya Health Bill 2016 tasks National Government with promoting health and countering any influences that have adverse effects on the population, including promotion of safe foods in sufficient quality and quantity, and promotion of nutrition knowledge. However, it is not specific to food/nutrition in relation to NCDs.

The food security unit within the Office of the President led the convening of policy dialogues, which is visible political support for dealing with food prices, food security, and food environment issues; **however**, population nutrition promotion was not a key focus of policy formulation. The quality of their human resource and the organisational effectiveness to contribute to information generation and to monitoring and evaluation, as well as their ties with the policy leaders, helped hasten debates and decision-making (17).

The launch of the Scaling Up Nutrition (SUN) Movement in Kenya in 2012 and the adoption of the National Nutrition Action Plan (NNAP) (2012 – 2017) gave momentum to nutrition, now high on the government's political agenda (15).

Food and Nutrition security is among the four priority areas, identified by the President of Kenya, as the key development agenda (Big Four Agenda) for the country, between 2017 1nd 2022. To achieve this, several new national initiatives have been identified/ proposed including enhanced large-scale agricultural production of food, promote the productivity of small holders (to enhance food availability) and reduction of food costs (to improve food availability)

International best practice examples (benchmarks) for LEAD1:

NEW YORK: As Mayor of New York City, Michael Bloomberg prioritised food policy and introduced a number of ground breaking policy initiatives including 'Health Bucks', a restriction on trans fats, establishment of an obesity taskforce, a portion size restriction on sugar-sweetened beverages, public awareness campaigns, etc. He showed strong and consistent leadership and a commitment to innovative approaches and cross-sectoral collaboration (86).

BRAZIL: The Minister of Health showed leadership in developing new dietary guidelines that are drastically different from the majority of dietary guidelines created by any nation to date, and align with some of the most commonly cited recommendations for healthy eating (87).

CARICOM COUNTRIES: Active NCD commissions exist in six of the 20 CARICOM member states (Bahamas, Barbados, Bermuda, British Virgin Islands, Dominica, Grenada) which are all housed in their Ministries of Health, with members recommended by the Minister of Health and appointed by the Cabinet of Government for a fixed duration; all include government agencies and to a varying degree, civil society and the private sector.

Good Practice Indicator - LEAD2: Clear population intake targets have been established by the government for the nutrients of concern to meet WHO and national recommended dietary intake levels.

Evidence of policy action on LEAD2:

The Food, Drugs and Chemical Substances Act Cap 254 (Amendment) 2015 dictates that edible vegetable fats and oils shall be free from trans fatty acids, and that food deemed to be free from trans fatty acids shall contain such amounts of trans fatty acids as may be permitted by the Codex Allimentarius (0.1 g trans-fat per 100 g or per 100 ml).

- The WHO Department of Nutrition for Health and Development (NHD), through the work of the Nutrition Guidance Expert Advisory Group (NUGAG) Subgroup on Diet and Health, is updating the population nutrient intake goals for the prevention of NCDs established in 1989 (by the WHO Study Group on Diet, Nutrition and Prevention of NCDs) and later updated in 2002 (by the Joint WHO/FAO Expert Consultation on Diet, Nutrition and the Prevention of Chronic Diseases). As a result of this work, WHO has already released updated guidance on intakes of sodium, potassium, and sugar. Furthering this work, guidelines on saturated fatty acid and trans fatty acid intake have been developed to provide recommendations on the intake of these fatty acids to reduce the risk of CVDs in adults and children, and for use by policy-makers and programmers to assess current intake levels relative to a benchmark, with a view to develop measures to reduce their intake. As part of this process, WHO Member States (including Kenya) and all relevant stakeholders (members of the community of nutrition and NCDs) have been invited to participate in this public consultation (May to June 2018). The finalisation of these guidelines may further action on this practice as relates to saturated and trans fatty acids.

International best practice examples (benchmarks) for LEAD2:

BRAZIL: The "Strategic Action Plan for Confronting NCDs in Brazil, 2011-2022 specifies a target of increasing adequate consumption of fruits and vegetables, from 18.2% to 24.3 % between 2010 and 2022 and reduction of the average salt intake of 12 g to 5 g, between 2010 and 2022(90).

SOUTH AFRICA: The South African plan for the prevention and control of non-communicable diseases includes a target on reducing mean population intake of salt to <5 grams per day by 2020 (91).

UK: In July 2015, the government adopted as official dietary advice the recommendation of the Advisory Committee on Nutrition that sugar should make up no more than 5% of daily calorie intake (30g or 7 cubes of sugar per day). Current sugar intake makes up 12 to 15% of energy. An evidence review by Public Health England outlines a number of strategies and interventions (92).

Good Practice Indicator - LEAD3: Clear, interpretive, evidence-informed food-based dietary guidelines have been established and implemented.

Evidence of policy action for LEAD3:

The Ministry of Health has developed National Guidelines for Healthy Diets and Physical Activity (which largely provide general guidance on healthy diets and physical activity among the general population). These national guidelines have clear dietary intake levels for salt, sugar, and fats for the elderly, but general statements on reduction of sugar, fat, and salt for other population groups (no specific dietary intake levels).The guidelines are a starting point for the development of Kenya's Food-Based Dietary Guidelines, which will provide population intake targets for nutrients of concern in relations to NCDs. In addition, a food composition table for Kenya is currently under development.

International best practice examples (benchmarks) for LEAD3:

BRAZIL: The national dietary guidelines of Brazil address healthy eating from a cultural, ethical and environmental perspective, rather than based on number of servings per food group. The main

recommendations are: 'Make natural or minimally processed foods the basis of your diet'; 'use oils, fats, salt, and sugar in small amounts for seasoning and cooking foods'; 'use processed foods in small amounts'; 'avoid ultra-processed foods'. They also provide advice on planning, shopping and sharing meals, as well as warning people to be wary of food marketing and advertising (93, 94).

Good Practice Indicator - LEAD4: There is a comprehensive, transparent, up-to-date and government-owned implementation plan, linked to national needs and priorities, to improve food environments, reduce the intake of the nutrients of concern to meet WHO and national recommended dietary intake levels, and reduce diet-related NCDs.

Evidence of policy action for LEAD4:

The Kenya National Strategy for the Prevention and Control of Non-Communicable Diseases (2015-2020), highlights that in Kenya, NCDs account for more than 50% of total hospital admissions and over 55% of hospital deaths, and yet, NCD prevention and control receives inadequate attention. This document, developed by the government in collaboration with key stakeholders, provides a framework for reducing preventable NCD-related morbidity and mortality. Promotion of healthy lifestyles including healthy diets is highlighted as a key strategic objective towards reducing NCDs disease risks.

The Ministry of Health's Unit of Nutrition has established a department on Healthy Diets and Physical Activity, which works closely with the Division of Non-Communicable Diseases to implement the NCD strategy including promotion of healthy diets for improved prevention and management of diet related NCDs. Consequently, the National Guideline for Healthy Diets and Physical Activity (2017), developed by government in collaboration with other stakeholders, provides guidance and key messages on healthy diets and physical activities for the general population, as a key strategy to address NCDs mortality and risks among other nutrition related diseases. This step serves as a starting point for the development Kenya's Food-Based Dietary Guidelines.

- These documents have support from the highest levels of office at the Ministry of Health, including but not limited to the Cabinet Secretary for Health, Director of Medical Services, and Head of the Nutrition Department.

In the Draft School Health Policy document, the prevention and management of NCDs in schools is also a key thematic area. Promotion of healthy lifestyles including healthy diets and physical activity in schools are highlighted as key strategies to reducing the incidence of NCDs among children and adolescents.

The Kenya Health Sector Strategic and Investment Plan (KHSSP) 2013-2017, provides the health sector and related sectors with medium-term focus, objectives, and priorities to enable them to move towards attainment of the Kenya Health Policy Directions. It follows on from the 2nd National Health Sector Strategic Plan (NHSSP II), which had the overall goal to reduce inequalities in health care services and reverse the downward trend in health-related outcome indicators. Strategic objective 2 within the KHSSP is to halt and reverse the rising burden of NCDs by providing prevention activities; implementing interventions directly addressing marginalised and indigent populations affected by NCDs; integrating health service provision tools, mechanisms and processes for NCDs; and, establishing screening programmes in health facilities for major NCDs. Among major NCDs targeted are: mental health, type 2 diabetes, cardiovascular diseases, chronic obstructive airway conditions, blood disorders focusing on Sickle cell conditions, and cancers, which contribute the most to the NCD burden.

- The Statistical Review of Progress towards the Mid-Term Targets of the Kenya Health Sector Strategic Plan 2014-2018 reviewed the evidence for progress made during the first half (2014-mid 2016) of the implementation of the Kenya Health Sector Strategic and Investment Plan (KHSSP), using all available data sources, and paying special attention to county progress. Data from the STEPS 2015 showed that the prevalence of overweight and obesity had increased dramatically in the last decade, especially among women (38% overweight or

obese), as observed in all counties with some variations in rates. Raised blood pressure (>=140/90 mm Hg) affected almost a quarter of adults (23%), with few diagnosed and treated, and only 4% of those on treatment had their blood pressure controlled. Diabetes prevalence was 1.9% among persons 15-69 years but only 41% had been diagnosed and effective treatment. Cervical cancer screening rates were low throughout Kenya with only one in 7 women 25-49 years (ever) screened. All these findings indicated that greater efforts were required to curb the rapid rise of NCDs and their risk factors.

One of the priority areas identified in The National Nutrition Action Plan (NNAP) (2012 – 2017) is the development of comprehensive strategies and guidelines for the prevention, management and control of diet related NCDs, as well as creating public awareness on the importance of prevention, management and control of diet related NCDs through a national Advocacy, Communication and Social Mobilisation (ACSM) strategy. One of the strategies employed to improve nutritional knowledge, attitudes, and practise is to mark nutrition days nationally and in all counties. Nutrition days include World Breastfeeding Week, African Food and Nutrition Security Day, Iodine Deficiency Disorders Day, and *Malezi Bora.*

The Kenya Health Bill 2016 tasks National Government with promoting health and countering any influences that have adverse effects on the population, including promotion of safe foods in sufficient quality and quantity, and promotion of nutrition knowledge. However, it is not specific to food/nutrition in relation to NCDs.

The food security unit within the Office of the President led the convening of policy dialogues, which is visible political support for dealing with food prices, food security, and food environment issues; **however**, population nutrition promotion was not a key focus of policy formulation. The quality of their human resource and the organisational effectiveness to contribute to information generation and to monitoring and evaluation, as well as their ties with the policy leaders, helped hasten debates and decision-making (17).

The launch of the Scaling Up Nutrition (SUN) Movement in Kenya in 2012 and the adoption of the National Nutrition Action Plan (NNAP) (2012 – 2017) gave momentum to nutrition, now high on the government's political agenda (15).

International best practice examples (benchmarks) for LEAD4:
EU: The European Food and Nutrition Action Plan 2015-20 outlines clear strategic goals, guiding principles, objectives, priorities and tools. The Plan aligns with the WHO Global Action Plan and under 'Objective 1 – Create healthy food and drink environments' there are clear policy and program actions identified (95).

Good Practice Indicator - LEAD5: Government priorities have been established to reduce inequalities or protect vulnerable populations in relation to diet, nutrition, obesity and NCDs.

Evidence of policy action for LEAD5:
The National Food and Nutrition Security Policy (2011) specifically identifies the importance of the protection of vulnerable populations using innovative and cost-effective safety nets linked to long-term development, since for the poorest and most vulnerable (e.g. in emergency relief situations), their health, nutrition status, and overall well-being, is at imminent and serious risk.

The National Nutrition Action Plan (NNAP) (2012 – 2017), managed by the Ministry of Health is being implemented nationwide and mainly focuses on nutrition-specific interventions, such as Vitamin A and Zinc supplementation or food fortification, targeting women of reproductive age and children under five (15).

These strategies address hunger and food insecurity concerns relating to diet and nutrition, rather than inequalities in food/nutrition in relation to obesity and NCDs.

Food and Nutrition security is among the four priority areas, identified by the President of Kenya, as the key development agenda (Big Four Agenda) for the country, between 2017 and 2022.To achieve this, several new national initiatives have been identified/ proposed including enhanced large-scale agricultural production of food, promote the productivity of small holders (to enhance food availability) and reduction of food costs (to improve food availability).'The big four' food nutrition and security agenda however, focuses mainly on food availability/ security rather than obesity and NCDs.

The Kenya Breast milk Substitutes Act (BMS act), was established in 2012, This is an act of Parliament, to direction for appropriate marketing and distribution of breast milk substitutes; to provide for safe and adequate nutrition for infants through the promotion of breastfeeding and proper use of breast milk substitutes, where necessary, and for connected purposes. The Act contains provisions for restriction on advertisement, promotion, packaging and educational and information material on breast milk substitutes and complementary feeding products. It also provides for the establishment and functions of a National Standing Committee to advise the Cabinet Secretary on the policy adoption and implementation in relation to the production, manufacture, sale, advertising, promotion and use of designated or complementary food products. This steering committee was established in 2014.

International best practice examples (benchmarks) for LEAD5:

NEW ZEALAND: The Ministry of Health reports the estimates derived from health surveys and nutrition surveys by four subpopulation groups including age group, gender, ethnic group and an area level deprivation index. Similarly, estimates derived from other data types (e.g. mortality) are presented by these subpopulation groups. The contracts between MoH and NGOs or other institutions include a section on Maori Health and state: "An overarching aim of the health and disability sector is the improvement of Maori health outcomes and the reduction of Maori health inequalities. You must comply with any: a) Maori specific service requirements, b) Maori specific quality requirements and c) Maori specific monitoring requirements". In addition, the provider quality specifications for public health services include specific requirements for Maori:" C1 Services meet needs of Maori, C2 Maori participation at all levels of strategic and service planning, development and implementation within organisation at governance, management and service delivery levels, C3: support for Maori accessing services". In the specific contract between the Ministry of Health and Agencies for Nutrition Action the first clause is on Maori Health: "you must comply with any Maori specific service requirements, Maori specific quality requirements and Maori specific monitoring requirements contained in the Service specifications to this agreement".

AUSTRALIA: The National Indigenous Reform Agreement (Closing the Gap) is an agreement between the Commonwealth of Australia and the States and Territories. The objective of this agreement is to work together with Indigenous Australians to Close the Gap in Indigenous disadvantage. The targets agreed to by COAG relate to health or social determinants of health. For the target 'Closing the life expectancy gap within a generation (by 2031)', one of the performance indicators is the prevalence of overweight and obesity.

DOMAIN 9 – GOVERNANCE (GOVER)

Governments have structures in place to ensure transparency and accountability, and encourage broad community participation and inclusion when formulating and implementing policies and actions to create healthy food environments, improve population nutrition, and reduce diet-related inequalities.

Good Practice Indicator - GOVER1: There are robust procedures to restrict commercial influences on the development of policies related to food environments where they have conflicts of interest with improving population nutrition.

Evidence of policy action for GOVER1:
The Kenya National Strategy for the Prevention and Control of Non-Communicable Diseases 2015-2020 states that public health policies, strategies, and multi-sectoral action for the prevention and control of NCDs must be protected from undue influence by any form of vested interest. Real, perceived or potential conflicts of interest must be acknowledged and managed.

The Kenya Health Bill 2016 tasks National Government with promoting health and countering any influences that have adverse effects on the population, including promotion of safe foods in sufficient quality and quantity, and promotion of nutrition knowledge. However, it is not specific to food/nutrition in relation to NCDs. This bill is still under review in Parliament.

We identified no further action specifically related to procedures for the restriction of commercial influences on the development of policies related to food environments.

International best practice examples (benchmarks) for GOVER1:
US**:** Mandatory and publicly accessible lobby registers exist at the federal level, as well as in nearly every state. Financial information must be disclosed, and the register is enforced through significant sanctions. A number of pieces of legislation uphold compliance with the register including Lobbying Disclosure Act of 1995 and the Honest Leadership and Open Government Act 2007.

NEW ZEALAND: The State Services Commission has published Best Practice Guidelines for Departments Responsible for Regulatory Processes with Significant Commercial Implications. They cover the development and operation of a regulatory process and include specific references to principles around stakeholder relationship management (96).

AUSTRALIA: The Australian Public Service Commission's Values and Code of Conduct includes a number of relevant sections such as the Conflict of Interest, Working with the Private Sector and other Stakeholders and the Lobbying Code of Conduct.

Good Practice Indicator - GOVER2: Policies and procedures are implemented for using evidence in the development of food policies.

Evidence of policy action for GOVER2:
The National Nutrition Action Plan (NNAP) (2012 – 2017) highlights enhancing evidence-based decision-making through operations research as a strategic objective.

The Kenya National Strategy for the Prevention and Control of Non-Communicable Diseases 2015-2020 recognises that interventions and implementation should focus on the achievement of well-formulated objectives, cost-effective, and evidence based targets, and that integrating research into national and county health programmes would generate actionable evidence about the effectiveness of interventions or policies.

The Ministry of Health's Department of Nutrition and Dietetics has a Research Unit, which is a platform for policy makers, implementers, and researchers (from government and non-

governmental institutions) to deliberate on research priorities, design research projects, validate external research concepts, and collate research evidence for policy formulation/influence.

Some nutrition guidelines and policy documents, such as the Breastfeeding Mother's Bill 2017, and the Kenya Baby Friendly Community Initiative (BFCI) have been developed with evidence from observational and implementation research.

Academic researchers in universities and think tanks have been a key source of evidence on policy relating to food security and during food price crisis (17).

International best practice examples (benchmarks) for GOVER2:
***AUSTRALIA*: ** The National Health and Medical Research Council Act 1992 (NHMRC Act) requires NHMRC to develop evidence-based guidelines. These national guidelines are developed by teams of specialists following a rigorous nine-step development process (97).

Good Practice Indicator - GOVER3: Policies and procedures are implemented for ensuring transparency in the development of food policies.

Evidence of policy action for GOVER3:
The National Food and Nutrition Security Policy (2011) calls for more predictable and transparent involvement of the state in the regulation of food trade and markets, particularly where changes to import tariffs, and/or, purchase and sale prices are under consideration.

Though not documented officially, there is clear involvement of non- governmental stakeholders in policy formulation processes, this is done through consultative meetings, technical working groups and forums throughout the policy formulation process, such working groups include the national IYCF steering committee, the MIYCN technical working group, the nutrition in research technical working group etc.

For transparency and democracy, the Kenyan constitution (public participation bill) allows for public participation in legislation and policy development, however, it is not specific on food policies

International best practice examples (benchmarks) for GOVER3:
AUSTRALIA/NEW ZEALAND: Food Standards Australia New Zealand (FSANZ) is required by the Food Standards Australia New Zealand Act 1991 to engage stakeholders in the development of new standards. This process is open to everyone in the community including consumers, public health professionals, and industry and government representatives. FSANZ has developed a Stakeholder Engagement Strategy 2013-16 that outlines the scope and processes for engagement. Under the Stakeholder Engagement Priorities 2013-16, it outlined "maintain our open and transparent approach" as one of the first priorities (98).

Good Practice Indicator - GOVER4: The government ensures access to comprehensive nutrition information and key documents (e.g. budget documents, annual performance reviews and health indicators) for the public.

Evidence of policy action for GOVER4:
The National Food and Nutrition Security Policy (2011) states that the government will ensure achievement of adequate nutrition for optimum health of all Kenyans through creating public awareness on relevant issues, and ensuring that all Kenyans are well informed about proper basic nutrition required to live a healthy and active lifestyle throughout the life cycle.

Some nutrition information (for example, maternal, infant and young child nutrition, food security and emergency nutrition, or nutrition and NCDs) is made available to the public though government websites, and at various government health, schooling, and community facilities (usually in the form of posters, briefs, or pamphlets). The government's unit of Nutrition and

Dietetics has a free access website, in which all food and nutrition related policies, National Survey Reports, and other indicators are available to the public. Various educative and communication materials such as pamphlets with a focus on health and nutrition have been developed by the government for use by the public, and issued at government-funded and other settings (for example, promoting exclusive breastfeeding, promoting appropriate complementary feeding after 6 months, nutrition in management of diabetes etc.)

We identified limited publicly available information on budget/spending in relation to health and nutrition.

Kenya's constitution, Article 35 of the Constitution and Section 96 of the County Government Act, 2012 provide for the right to access of information. Article 35(1) particularly guarantees all Kenyan citizens the right to state or information held by another person and required for the exercise or protection of any right or fundamental freedom.

The Access to Information Bill, 2015 covers public bodies or information held by another person and required for the exercise or protection of any right or fundamental freedom. It also applies to private entities that receive public resources and benefits, utilise public funds, engage in public functions, provide public services or have exclusive contracts to exploit natural resources. These documents are however not specific to nutrition information.

International best practice examples (benchmarks) for GOVER4:
AUSTRALIA/NEW ZEALAND: The Freedom of Information Act provides a legally enforceable right of the public to access documents of government departments and most agencies.

DOMAIN 10 - MONITORING AND EVALUATION (MONIT)

The government's monitoring and evaluation systems (surveillance, evaluation, research and reporting) are comprehensive and regular enough to assess the status of food environments, population nutrition and diet-related NCDs and their inequalities, and to measure progress on achieving the goals of nutrition and health plans.

Good Practice Indicator - MONIT1: Monitoring systems, implemented by the government, are in place to regularly monitor food environments (especially for food composition for nutrients of concern, food promotion to children, and nutritional quality of food in schools and other public sector settings), against codes/guidelines/standards/targets.

Evidence of policy action for MONIT1:

There were no polices found specifically targeting the monitoring of food environments and quality of food for nutrients of concern as relates to obesity and NCDs. The National Nutrition Action Plan (NNAP) (2012 – 2017) includes objectives to:

- Strengthen monitoring and evaluation of systems for diet-related NCDs so as to improve the prevention, management and control of diet related NCDs.
- Strengthen monitoring and evaluation of nutrition intervention in schools and other institutions.

Development of an updated Food Composition Table for Kenya has been completed and will be launched in August 2018, led by the Ministry of Health and the Ministry of Agriculture, Livestock and Fisheries in Kenya and the Food and Agriculture Organization of the United Nations (FAO) in consultation with contributors from various academic and research institutions, laboratories, and programmes. The updated food composition table has more elaborate presentation of the nutrient information and contains more nutrient components compared to the older version that was developed in 1993.

The **Kenya Bureau of Standards** (**KEBS**) is a government agency responsible for governing and maintaining the standards and practices relating to products, measurements, materials, and processes, as well as their promotion at national, regional, and international levels. This includes food products and processes. **However**, we found no evidence of monitoring of the food environment and quality of food for nutrients of concern as relates to obesity and NCDs.

A national Maternal Infant and Young Child Committee was established to facilitate the implementation and monitoring of the breast milk substitutes bill.

International best practice examples (benchmarks) for MONIT1:

MANY COUNTRIES: Many countries do have food composition databases available. For example, the New Zealand Institute for Plant & Food Research Limited and the Ministry of Health jointly own the New Zealand Food Composition Database (NZFCD) which is a comprehensive collection of nutrient data in New Zealand containing nutrient information on more than 2600 foods.

NEW ZEALAND: A national School and Early Childhood Education (ECE) Services Food and Nutrition Environment Survey was organised in all Schools and ECES across New Zealand in 2007 and 2009 by the Ministry of Health to measure the food environments in schools and ECEs in New Zealand.

UK: in October 2005, the School Food Trust ('the Trust'; now called the Children's Food Trust) was established to provide independent support and advice to schools, caterers, manufacturers and others on improving the standard of school meals. They perform annual surveys, including the latest information on how many children are having school meals in England, how much they cost and how they're being provided (99).

Good Practice Indicator - MONIT2: There is regular monitoring of adult and childhood nutrition status and population intakes against specified intake targets or recommended daily intake levels.

Evidence of policy action for MONIT2:

The National Nutrition Action Plan's (2012 – 2017) strategic objective 6 focuses on improved prevention management and control of diet related non- communicable diseases, one of the indicators in this objective is to conduct regular population screening for non- communicable diseases and food consumption.

In addition, standard tools and methodologies for conducting food consumption and nutrition surveys have been developed, including the SMART survey, the Knowledge Attitude Behaviour and Practices (KABP) tools. The health Information Systems (HIS) also enables data collection within the health system (Health facilities) that could be used for monitoring, evaluation and programming, however the quality of the data is often compromised and it is also not representative as it is collected from the sick population visiting the health facilities and not from the general population.

There are examples of national surveys which have assessed population nutrition status and dietary intakes against specific recommended targets.

- The Kenya Stepwise Survey for Non-Communicable Diseases (2015)
- The Kenya Demographic and Health Survey (2014)
- The Kenya Micronutrient Survey (2011)

These have not been completed regularly, further, there is no evidence that the recommendations within these reports that have been implemented.

International best practice examples (benchmarks) for MONIT2:
US: The National Health and Nutrition Examination Survey (NHANES) is a program of studies designed to assess the health and nutritional status of adults and children in the United States. The survey is unique in that it combines interviews and physical examinations (100). The NHANES program began in the early 1960s and has been conducted as a series of surveys focusing on different population groups or health topics. In 1999, the survey became a continuous program that has a changing focus on a variety of health and nutrition measurements to meet emerging needs. The survey examines a nationally representative sample of about 5,000 persons each year. These persons are located in counties across the country, 15 of which are visited each year.

Good Practice Indicator - MONIT3: There is regular monitoring of adult and childhood overweight and obesity prevalence using anthropometric measurements.

Evidence of policy action for MONIT3:
The Kenya National Strategy for the Prevention and Control of Non-Communicable Diseases 2015-2020 calls for periodic surveys of NCDs and their risk factors (including anthropometry), and allocation of resources for the same at county level. Examples of surveys that have been completed include:

- The Kenya Stepwise Survey for Non-Communicable Diseases (2015) was the first nationally representative survey aimed at collecting comprehensive information on risk factors for NCDs in adults aged 18–69 years in the country (not including children). Anthropometric measurements were taken for the determination of overweight and obesity. **However**, these measures are not undertaken regularly.
- The Kenya Demographic and Health Surveys include anthropometric data collection for children, adolescents, and women of reproductive age. Obesity is reported as among the key health indicators in the country.
- The Kenya National Micronutrient Survey (last conducted in 2011 - 2012), includes anthropometric data collection for both children and adolescent and overweight/obesity is report among the key health indicators in the country.

International best practice examples (benchmarks) for MONIT3:
UK: England's National Child Measurement Programme was established in 2006 and aims to measure all children in England in the first (4-5 years) and last years (10-11 years) of primary school. In 2011-2012, 565,662 children at reception and 491,118 children 10-11 years were measured (101).

Good Practice Indicator - MONIT4: There is regular monitoring of the prevalence of NCD risk factors and occurrence rates (e.g. prevalence, incidence, mortality) for the main diet-related NCDs.

Evidence of policy action for MONIT4:
The Kenya National Strategy for the Prevention and Control of Non-Communicable Diseases 2015-2020 calls for periodic surveys of NCDs and their risk factors, and allocation of resources for the same at county level.

The Kenya Stepwise Survey for Non-Communicable Diseases (2015) was the first nationally representative survey aimed at collecting comprehensive information on risk factors for NCDs among adults aged 18–69 years in the country. **However**, these measures are not done regularly.

International best practice examples (benchmarks) for MONIT4:
OECD COUNTRIES: Most OECD countries have regular and robust prevalence, incidence and mortality data for the main diet-related NCDs and NCD risk factors

Good Practice Indicator - MONIT5: There is sufficient evaluation of major programmes and policies to assess effectiveness and contribution to achieving the goals of the nutrition and health plans.

Evidence of policy action for MONIT5:
The National Nutrition Action Plan (NNAP) (2012 – 2017) highlights the need for enhanced quality and timeliness of data collected so as to ensure effective decision making, through:

- Operationalization of the nutrition monitoring and evaluation framework for the nutrition sector
- Reviewing, development and dissemination of the appropriate guidelines and tools for surveillance, monitoring and evaluation
- Strengthening feedback mechanisms on nutrition information among nutrition stakeholders
- Training managers and service providers on use of DHIS and interpretation of monitoring and evaluation data
- Strengthening the integration of nutrition indicators in the existing integrated disease surveillance system
- Promoting use of appropriate technology to enhance quality of data collected

A nutrition information technical working group (NITWG), and the Research in Nutrition department within the Ministry of Health's Unit of Human Nutrition and Dietetics is charged with the responsibility of monitoring and evaluation of key nutrition interventions in the country.

International best practice examples (benchmarks) for MONIT5:
US: The National Institutes for Health (NIH) provide funding for rapid assessments of natural experiments. The funding establishes an accelerated review/award process to support time-sensitive research to evaluate a new policy or program expected to influence obesity related behaviours (e.g., dietary intake, physical activity, or sedentary behaviour) and/or weight outcomes in an effort to prevent or reduce obesity(102).

Good Practice Indicator - MONIT6: Progress towards reducing health inequalities or health impacts in vulnerable populations and societal and economic determinants of health are regularly monitored.

Evidence of policy action for MONIT6:

A number of key national strategies/policies have a focus on monitoring vulnerable populations; however, we identified less evidence of regular monitoring:

- The Kenya National Strategy for the Prevention and Control of Non-Communicable Diseases 2015-2020 relies on the guiding principle of an "equity-based approach", since the disparity in occurrence of NCDs is due to unequal distribution of social determinants of health.
- The National Food and Nutrition Security Policy (2011) specifically identifies the importance of the protection of vulnerable populations as linked to long-term development, since their health and nutrition status and overall well-being is at imminent and serious risk.
- The Kenya National Bureau of Statistics (KNBS) conducts periodic surveys on health indicator disparities (e.g. mortality and fertility) through the Kenya Demographic and Health Survey, and socioeconomic determinants of health (e.g. through their economic survey), both of which have shown critical geographic and gender disparities in some health indicators.
- The National Health Insurance Fund is a government's scheme, aimed at providing accessible, affordable, sustainable, and equitable and quality social health insurance through optimal utilization of resources. The national scheme seeks to ensure that every individual and/or family enjoy comprehensive In-patient and Out-patient covers to save them from the effects of unpredictable and catastrophic spending on medical bills, and has special subsidies for vulnerable populations including the poor, and elderly, people with severe disabilities and pregnant women and infants. The NCD's related treatment costs including dialysis, oncology and radiology are among the costs covered by NHIF.
- There has been limited focus on the reduction of health inequalities as relates to the disparity in occurrence of NCDs.

International best practice examples (benchmarks) for MONIT6:

NEW ZEALAND: All annual Ministry of Health Surveys report estimates by subpopulations in particular by ethnicity (including Maori and Pacific peoples), by age, by gender, and by New Zealand area deprivation

DOMAIN 11 - FUNDING AND RESOURCES (FUND)

Sufficient funding is invested in 'Population Nutrition Promotion' (as estimated from the investments in population promotion of healthy eating and healthy food environments for the prevention of obesity and diet-related NCDs) to create healthy food environments, improved population nutrition, reductions in obesity, diet-related NCDs and their related inequalities.

This excludes funding investments in one-on-one promotion (e.g. primary care, antenatal services, maternal and child nursing services etc.), food safety, micronutrient deficiencies (e.g. folate fortification) and under nutrition).

Good Practice Indicator - FUND1: Funding for the promotion of healthy eating and healthy food environments, as a proportion of total health spending and/or in relation to the diet-related NCD burden is sufficient to reduce obesity and diet-related NCDs.

Evidence of policy action for FUND1:

We identified limited, clear information on the level of funding for the promotion of healthy eating and healthy food environments, as a proportion of total nutrition spending (14). Some evidence provided insight into more general commitments for/spending on nutrition:

- The National Nutrition Action Plan (NNAP) (2012 – 2017) recommends that the government allocates 2.0% of the health budget for nutrition.
- The total cost of the NNAP over five years is Kshs 70 billion (approximately US$826 million), averaging US$165 million per year, with a per capita annual cost of less than US$4. The costing of the NNAP, conducted over a three month period, was spearheaded by the Kenyan Government and UNICEF, with input from additional stakeholders (14).
- Government spending on nutrition-specific interventions through the health sector was estimated at 0.5% of the annual health budget, an investment that has been growing at a rate of 0.1- 0.2 % annually for the past five years. Nutrition budgets are now mainstreamed as the NNAP is aligned to the government's broader Medium Term Development Plan. The previous year's annual operating budget for the Department of Nutrition was around US$10 million. Alignment of donor funding with the national nutrition plan is underway. DFID announced a £16.8 million (US$26 million) three-year funding package for nutrition-specific interventions targeting emergency-prone areas of the country when the NNAP was launched. Additional humanitarian multi-year funding will be provided through the €250 million (US$ 326 million) European Union initiative, called Supporting Horn of Africa Resilience (SHARE) (14).

International best practice examples (benchmarks)

NEW ZEALAND: The total funding for population nutrition was estimated at about $67 million or 0.6% of the health budget during 2008/09 Healthy Eating Healthy Action period. Dietary risk factors account for 11.4% of health loss in New Zealand.

THAILAND**:** According to the most recent report on health expenditure in 2012 the government greatly increased budget spent on policies and actions related to nutrition (excluding food, hygiene and drinking water control). Total expenditure on health related to nutrition specifically from local governments was 29,434.5 million Baht (about 840 million USD) (7.57% of total health expenditure from public funding agencies), which was ten times over the budget spending on nutrition in 2011. Dietary risk factors account for about 10% of health loss in Thailand.

Good Practice Indicator - FUND2: Government funded research is targeted at improving food environments, reducing obesity, NCDs and their related inequalities.

Evidence of policy action for FUND2:

The National Food and Nutrition Security Policy (2011) states that the government will initiate appropriate measures, including research, aimed at improving food quality and safety. Further, that the government will also build capacity and ensure the availability of quality and timely food security and nutrition data, information, and analysis, for better formulation and management of integrated food security and nutrition policies, programmes, and action. **However**, the focus above

seems to be geared towards sustainable production and availability of food that is diversified, affordable, and helps meet basic nutrition requirements, rather than food/nutrition in relation to obesity and NCDs.

The Science, Technology and Innovation Act (2013), was passed by the Parliament to facilitate the promotion, co-ordination and regulation of the progress of science, technology and innovation of the country; to assign priority to the development of science, technology and innovation; to entrench science, technology and innovation into the national production system and for connected purposes. The act provides for the establishment of a national research fund, that consist of a sum of money amounting to two per cent of the country's gross domestic product, provided by the treasury every financial year, the objective of the fund is to facilitate research for the advancement of science, technology and innovation.

The National Research Fund was established in 2015, to support advancement of scientific research, inventions and Innovations and build capacity in ST&I sector for national development.

The fund is however not specific to research on NCDs or the food environment

International best practice examples (benchmarks) for FUND2
AUSTRALIA: The National Health and Medical Research Council (NHMRC) Act requires the CEO to identify major national health issues likely to arise. The National Health Priority Areas (NHPAs) articulate priorities for research and investment and have been designated by Australian governments as key targets because of their contribution to the burden of disease in Australia. For the 2015-16 Corporate Plan, obesity, diabetes and cardiovascular health are three of these NHPAs.

THAILAND: The National Research Council funded more research projects on obesity and diet-related chronic diseases (such as diabetes, cardiovascular diseases and hypertension) in 2014, accountable for almost six times over the research funding in 2013 (from 6,875,028 Baht in 2013 to 37,872,416 baht in 2014).

Good Practice Indicator - FUND3: There is a statutory health promotion agency in place that includes an objective to improve population nutrition, with a secure funding stream.

Evidence of policy action for FUND3:
The Kenya National Strategy for the Prevention and Control of Non-Communicable Diseases 2015-2020 states that action on NCDs must happen in 3 domains:
(i) Disease Prevention and Health Promotion,
(ii) Early Diagnosis and Control of NCDs through Health Systems Strengthening,
(iii) Monitoring, Surveillance and Research.

There is a dedicated Ministry of Health - Unit of Health Promotion, working with other health units including NCDs and Nutrition on health promotion. However, there is no funding specific for promotion of optimal population nutrition.

An Act of Parliament to establish machinery for making available to the Government advice upon all matters relating to the scientific and technological activities and research necessary for the proper development of the Republic; and for the co-ordination of research and experimental development.

International best practice examples (benchmarks) for FUND3:
AUSTRALIA: The Victorian Health Promotion Foundation (VicHealth) was the world's first health promotion foundation, established by the Victorian Parliament as part of the Tobacco Act of 1987 (for the first 10 years through a hypothecated tobacco tax) through which the objectives of VicHealth are stipulated. VicHealth continues to maintain bipartisan support.

DOMAIN 12 - PLATFORMS FOR INTERACTION (PLATF)

There are coordination platforms and opportunities for synergies across government departments, levels of government, and other sectors (NGOs, private sector, and academia) such that policies and actions in food and nutrition are coherent, efficient and effective in improving food environments, population nutrition, diet-related NCDs and their related inequalities.

Good Practice Indicator - PLATF1: There are coordination mechanisms across departments and levels of government (national and local) to ensure policy coherence, alignment, and integration of food, obesity, and diet-related NCD prevention policies across governments.

Evidence of policy action for PLATF1:
Some policy documents related to food, obesity, and diet-related NCD prevention have been developed by collaborating government ministries, with input from wider stakeholders. It has been reported that, despite an increasing consensus on the need to adopt a holistic approach to nutrition, formal multi-sectoral and inter-ministerial collaboration is lacking and nutrition-sensitive interventions are not adequately promoted (15). It has also been reported that responsibilities for agriculture, nutrition, and food security lie in different ministries, with minimal formal coordination among them.

However, discussions are ongoing regarding the proposal to develop a road map for the establishment of a coordination structure and implementation framework for the Food Security and Nutrition Policy. Such a structure would ensure coordination across sectors with the aim of overcoming the existing inter-ministerial divide and promoting an agreed agenda and work plan for the implementation of food security and nutrition policies and interventions (15).

International best practice examples (benchmarks) for PLATF1:
FINLAND: The Finnish National Nutrition Council is an inter-governmental expert body under the Ministry of Agriculture and Forestry with advisory, coordinating and monitoring functions. It is composed of representatives elected for three-year terms from government authorities dealing with nutrition, food safety, health promotion, catering, food industry, trade and agriculture (59).

MALTA: Based on the Healthy Lifestyle Promotion and Care of NCDs Act (2016), Malta established an inter-ministerial Advisory Council on Healthy Lifestyles in August 2016 to advise the Minister of Health on any matter related to healthy lifestyles. In particular, the Advisory Council advises on a life course approach to physical activity and nutrition, and on policies, action plans and regulations intended to reduce the occurrence of NCDs. The prime minister appoints the chair and the secretary of the Advisory Council, while the ministers of education, health, finance, social policy, sports, local government, and home affairs appoint one member each (59).

PACIFIC COUNTRIES: In 2014, the Pacifici Non-Communicable Disease Partnership was established to encourage a multi-sector approach to prevent and control non-communicable diseases. The partnership includes Pacific Island Forum Leaders, Pacific Ministers of Health, Pacific Islands Permanent Missions at the United Nations, Pacific Island Countries and Territories, Secretariat of the Pacific Community, World Health Organization, United Nations Development Programme, World Bank, Australia Department of Foreign Affairs and Trade, New Zealand Aid Programme, US Department of State, Pacific Island Health Officers' Association and the NCD Alliance. The partnership aims to strengthen and coordinate capacity and expertise to support Pacific Island countries achieve globally agreed NCD targets and implement the Pacific Islands NCD Roadmap.

AUSTRALIA: There are several forums and committees for the purpose of strengthening food regulation with representation from New Zealand and Health Ministers from Australian States and Territories, the Australian Government, as well as other Ministers from related portfolios (e.g. Primary Industries). Where relevant, there is also representation from the Australian Local Government Association.

Good Practice Indicator - PLATF2: There are formal platforms between government and the commercial food sector to implement healthy food policies.

Evidence of policy action for PLATF2:

The **Kenya Private Sector Alliance (KEPSA)** is a proponent of national nutrition plans, and attended a meeting by the **Kenya SUN Nutrition Private Sector Network (SNPSN)** also referred to as Scaling up **Nutrition Business Network**. The meeting was convened by the Global Alliance for Improved Nutrition (GAIN) and the Ministry of Health (MOH) under the theme of **'Enhancing collaboration with the private sector in Kenya'** focused on recruiting more members with an aim of improving contribution towards enhanced nutrition in Kenya.

- The goal of SNPSN is to act as the leading platform and centre of expertise for promoting action by business to scale-up and sustain the delivery of, and access to, improved nutrition for public health impact. SNPSN aims to achieve this through the establishment of a community which champions and mobilises business behind the National Nutrition Action Plan and supports the SUN Movement, to ensure that all people have access to good food and nutrition in Kenya. A SBPSN meeting was held on 15 September 2016 – the second workshop on sensitisation of the business, since Kenya joined the SUN movement in 2012.

International best practice examples (benchmarks) for PLATF2:

UK: The UK 'Responsibility Deal' was a UK government initiative to bring together food companies and non-government organisations to take steps (through voluntary pledges) to address NCDs during 2010-2015. It was chaired by the Secretary of State for Health and included senior representatives from the business community (as well as NGOs, public health organisations and local government). A number of other subgroups were responsible for driving specific programs relevant to the commercial food sector.

Good Practice Indicator - PLATF3: There are formal platforms for regular interactions between government and civil society on food policies and other strategies to improve population nutrition.

Evidence of policy action for PLATF3:

In 2012, the Republic of Kenya joined the **SUN Movement** with a letter of commitment from then Minister for Public Health and Sanitation. At the time, Kenya had adopted strategic policy initiatives and had been instigating multi-sectoral, legislative and constitutional courses of action. The National Nutrition Action Plan was in the process of formation and in nutrition legislation, and a Breast Milk Substitutes Regulation and Control Bill had been submitted to parliament. Scaling up Nutrition (SUN) is a global movement which unites governments, civil society, the United Nations, donors, businesses, and researchers in a collective effort to improve nutrition.

- Efforts to scale up nutrition in Kenya are coordinated by the Nutrition Inter Agency Coordination Committee (NICC) and supported by the Nutrition Forum and a number of technical working groups. A Scaling Up Nutrition Secretariat falls under the NICC to strengthen cross-sector coordination. The European Union serves as the SUN Donor Convenor, with a focus on mapping and financial tracking (14).

International best practice examples (benchmarks) for PLATF3:

BRAZIL: The National Council of Food and Nutrition Security (CONSEA) is a body made up of civil society and government representatives, which advises the President's office on matters involving food and nutrition security (103). CONSEA is made up from one-third government and two-thirds non-government executives and workers. It has special powers. It is housed in and reports to the office of the president of the republic. It is responsible for formulating and proposing public policies whose purpose is to guarantee the human right to healthy and adequate food. There are also CONSEAs at state and municipal levels that deal with specific issues, also responsible for organising CONSEA conferences at their levels. CONSEAs are charged to represent Brazilian social, regional, racial and cultural diversity at municipal, state or national level. The elected politicians in Brazil's parliament formally have the power to challenge and even overturn proposals made by CONSEA. In practice it is most unlikely that any Brazilian government whether of the

left or right would wish to do so, partly because of the constitutional status of the CONSEA system, and also because, being so carefully representative of all sectors and levels of society, it remains strong and popular.

DOMAIN 13 - HEALTH IN ALL POLICIES (HIAP)

Processes are in place to ensure policy coherence and alignment, and that population health impacts are explicitly considered in the development of government policies.

Good Practice Indicator - HIAP1: There are processes in place to ensure that development of all government policies relating to food are sensitive to nutrition, public health, and reducing health inequalities in vulnerable populations.

Evidence of policy action for HIAP1:
A number of different population health policies recognise the importance of a HiAP approach:

- The **Kenya Health Policy 2014-2030** is designed to be all-inclusive, balanced, and rational. It concentrates on two major obligations of health, including contribution to economic development provided in the New Constitution of Kenya 2010 and Kenya Vision 2030. It ensures equity, efficiency, and social accountability in the delivery of health services, and gives directions to ensure significant improvement in overall status of health in Kenya, demonstrating the health sector's commitment, under the government's stewardship, to ensuring that the country attains the highest possible standards of health, in a manner responsive to the needs of the population.
- The Kenya National Cancer Control Strategy 2017-2022 highlights the need for a multi-sectoral approach, the need to involve both health and non-health sectors, and the need to incorporate health in all sectors to effectively address cancer and associated risk factors. There also needs to be ownership and involvement of all stakeholders at all stages of implementation.
- The Kenya Health Sector Strategic and Investment Plan (KHSSP) 2013-2017, provides the Health Sector and related Sectors objectives and priorities to enable them move towards attainment of the Kenya Health Policy Directions. It is not restricted to the actions of the Health Ministry, but includes all actions in other related sectors that have an impact on health. An overall goal was to reduce inequalities in health care services and reverse the downward trend in health-related outcome indicators.
- Statistical Review of Progress towards the Mid-Term Targets of the Kenya Health Sector Strategic Plan 2014-2018 reviewed evidence for progress made during the first half of the implementation of the Kenya Health Sector Strategic and Investment Plan (KHSSP), and found some sex-disparities in the prevalence of risk for NCDs. The report concludes that greater efforts are required to curb the rapid rise of NCDs and their risk factors.

Other specific activities that have taken place on HiAP includes the Health in All Policies: Report on perspectives and intersectoral action in the African Region (2013), which was prepared by the Social Determinants of Health Unit under the Health Promotion Cluster - WHO African Region, with financial support from the Rockefeller Foundation. Dr Doris Kirigia (Kenya) represented Kenya on the technical writing group.

- Evidence in the report indicates that Kenya was among the countries that had conducted national seminars/workshops on initial training of different stakeholders involved in the coordination and implementation of interventions on social determinants of health. This included a call to action.
- That Kenya ranked high among countries with existing platforms for intersectoral partnerships and actions, including the involvement of wider stakeholders outside government.
- That among respondents surveyed from respective countries, 55% of Kenyan respondents mentioned the existence of various types of intersectoral coordination and leadership mechanisms in the country.
- In Kenya, health issues were particularly included in education policies.

International best practice examples (benchmarks) for HIAP1:
SLOVENIA: Slovenia undertook a Health Impact Assessment (HIA) in relation to agricultural policy at the national level. This was the first time that the health effects of an agricultural policy were assessed at the country level. The HIA followed a six-stage process: policy analysis; rapid appraisal workshops with stakeholders from a range of backgrounds; review of research evidence relevant to the agricultural policy; analysis of Slovenian data for key health-related indicators; a report on the findings to a key cross-government group; and evaluation (107).

AUSTRALIA: Established in 2007, the successful implementation of Health in All Policies (HiAP) in South Australia has been supported by a high level mandate from central government, an overarching framework which is supportive of a diverse program of work, a commitment to work collaboratively and in partnership across agencies, and a strong evaluation process. The government has established a dedicated HiAP team within South Australia Health to build workforce capacity and support Health lens Analysis projects (108). Since 2007, the South Australian HiAP approach has evolved to remain relevant in a changing context. However, the purpose and core principles of the approach remain unchanged. There have been five phases to the work of HiAP in South Australia between 2007 and 2016: 1) Prove concept and practice emerges (2007-2008), 2) Establish and apply methodology (2008-2009), 3) Consolidate and grow (2009-2013), 4) Adapt and review (2014) and 5) Strengthen and systematise (2015-2016).

FINLAND: Finland worked towards a Health in All Policies (HiAP) approach over the past four decades (109). In the early 1970s, improving public health became a political priority, and the need to influence key determinants of health through sectors beyond the health sector became evident. The work began with policy on nutrition, smoking and accident prevention. Finland adopted HiAP as the health theme for its EU Presidency in 2006.

POLICY AND STATEGY DOCUMENTS REVIEWED

INCLUDED		
**Name of Document**	**Date Published**	**Evidence Type**
Kenya Food and Nutrition Security Policy	2011	POLICY/STRATEGY/GUIDELINE
Food Drugs and Chemical Substance Act	1978	LEGISLATION
East African Standards - DRAFT	2015	POLICY/STRATEGY/GUIDELINE
Advertising Standards Body of Kenya - Code of Advertising Practice and Direct Marketing (Parts I and II)	2003	POLICY/STRATEGY/GUIDELINE
National School Health Policy - DRAFT	DRAFT	POLICY/STRATEGY/GUIDELINE
Breast Milk Substitutes Act (Regulation and Control) Act No.34 of 2012	2012	LEGISLATION
Budget Statement for the Fiscal Year 2017-2018	2017	BUDGETARY DOCUMENT
Excise Duty Act	2015	LEGISLATION
National Nutrition Action Plan (NNAP)	2012	POLICY/STRATEGY/GUIDELINE
School Nutrition and Meals Strategy for Kenya	2016	POLICY/STRATEGY/GUIDELINE
Kenya National Strategy for the Prevention and Control of Non-Communicable Diseases	2015	POLICY/STRATEGY/GUIDELINE
National Guideline for Healthy Diets and Physical Activity	2017	POLICY/STRATEGY/GUIDELINE
Kenya Health Bill	2016	LEGISLATION
Kenya Health Sector Strategic and Investment Plan (KHSSP)	2013	POLICY/STRATEGY/GUIDELINE
Statistical Review of Progress Towards the Mid-Term Targets of the Kenya Health Sector Strategic Plan	2014	EVALUATION/AUDIT/RESEARCH REPORT
Breastfeeding Mothers Bill	2007	LEGISLATION
Kenya Health Policy 2014-2030	2014	POLICY/STRATEGY/GUIDELINE
New Constitution of Kenya	2010	LEGISLATION
Kenya Vision 2030	2008	POLICY/STRATEGY/GUIDELINE
Kenya National Cancer Control Strategy	2017	POLICY/STRATEGY/GUIDELINE
Health in All Policies: Report on Perspectives and Intersectoral Actions in the African Region	2013	PRACTICAL/PROGRAMME GUIDANCE
Nutrition Labelling - Requirements [KS EAS 803:2014]	2014	POLICY/STRATEGY/GUIDELINE
Use of Nutrition and Health Claims - Requirements [KS EAS 805:2014]	2014	POLICY/STRATEGY/GUIDELINE
Labeling of Prepackaged Foods - Specification [KS EAS 38:2014]	2014	POLICY/STRATEGY/GUIDELINE
Food Drugs and Chemical Substance Act (Food Labelling, Additives and Standard (Amendment) Regulation 2015)	2015	LEGISLATION
Big 4 Agenda		WEBPAGE
National Health Insurance Fund		WEBPAGE
Draft school health policy 2017		POLICY/STRATEGY/GUIDELINE

REFERENCES

1. Vorster HH, Kruger A, Margetts BM. The nutrition transition in Africa: can it be steered into a more positive direction? Nutrients. 2011 Apr;3(4):429–41.
2. NIH National Institute of Diabetes and Digestive and Kidney Diseases. Overweight and Obesity Statistics. WIN Weight Inf Netw. 2010;04(4158):6.
3. WHO. WHO | Obesity and overweight. World Heal Organ Media Cent Fact Sheet No 311. 2012;
4. Troesch B, Biesalski HK, Bos R, Buskens E, Calder PC, Saris WHM, et al. Increased Intake of Foods with High Nutrient Density Can Help to Break the Intergenerational Cycle of Malnutrition and Obesity. Nutrients. 2015 Jul;7(7):6016–37.
5. FAO. Population nutrient intake goals for preventing diet-related chronic diseases. 2017.
6. Ministry of Health. Kenya National Strategy for the prevention and control of Non-communicable Disease, 2015–2020. 2015.
7. Vorley B, Lançon F. Food consumption , urbanisation and rural transformation The trade dimensions. 2016;(May).
8. Hawkes C. Globalization and Health Uneven dietary development : linking the policies and processes of globalization with the nutrition transition , obesity and diet-related chronic diseases. 2006;18:1–18.
9. Shetty PS. Nutrition transition in India. 2017;5:175–82.
10. Kenya National Bureau of Statistics (KNBS); ICF Macro. Kenya Demographic and Health Survey 2014. Heal (San Fr. 2014;1–314.
11. Vandevijvere S, Dominick C, Devi A, Swinburn B. The healthy food environment policy index: findings of an expert panel in New Zealand. Bull World Health Organ [Internet]. 2015;93(5):294–302. Available from: http://www.who.int/entity/bulletin/volumes/93/5/14-145540.pdf
12. d'Hôtel EM, Le Cotty T, Jayne, T. Trade policy inconsistency and maize price volatility: An ARCH approach in Kenya. African Development Review. 2013;25(4):607-620.
13. Nzuma JM. The political economy of food price policy: The case of Kenya (No. 2013/026). WIDER Working paper. 2013.
14. Scaling Up Nutrition [SUN] Movement. Planning and costing for the acceleration of actions for nutrition: experiences of countries in the Movement for Scaling Up Nutrition. 2014.
15. FAO. Country fact sheet on food and agriculture policy trends. [Internet]. 2015. Available from http://www.fao.org/3/a-i4911e.pdf
16. Neervoort F, von Rosenstiel I, Bongers K, et al. Effect of a school feeding programme on nutritional status and anaemia in an urban slum: A preliminary evaluation in Kenya. Journal of Tropical Pediatrics 2013;59(3):165-74. doi: 10.1093/tropej/fms070.
17. Babu SC. Policy process and food price crisis: A framework for analysis and lessons from country studies (No. 2013/070). WIDER Working paper. 2013.
18. WHO. Marketing of Breast-milk substitutes: National implementation of the International Code Status Report 2018. 2018. Available from: http://apps.who.int/iris/bitstream/handle/10665/272649/9789241565592-eng.pdf

References for international best practice examples

1. Allemandi L, Tiscornia MV, Ponce M, Castronuovo L, Dunford E, Schoj V. Sodium content in processed foods in Argentina: compliance with the national law. Cardiovasc Diagn Ther. 2015;5(3):197-206.
2. World Cancer Research Fund. NOURISHING Framework - Improving the food supply: World Cancer Research Fund; 2016 [19/02/2016]. Available from: http://www.wcrf.org/int/policy/nourishing-framework/improve-food-supply
3. Ministerio de Salud. Ley 26.905 Consumo de sodio. Valores Máximos. 2013 [16/06/2016]. Available from: http://www.msal.gob.ar/ent/images/stories/programas/pdf/2014-08_Ley26905-Ley-Sodio.pdf
4. Hofman KJ, Tollman SM. Population health in South Africa: a view from the salt mines. The Lancet Global health. 2013;1(2):e66-7.
5. Department of Health. Regulations relating to the reduction of sodium in certain foodstuffs and related matters: Staatskoerant; 2013 [16/06/2016]. Available from: http://www.heartfoundation.co.za/sites/default/files/articles/South%20Africa%20salt%20legislation.pdf
6. Astrup A. The trans fatty acid story in Denmark. Atheroscler Suppl. 2006;7(2):43-6.
7. Official Journal of the European Union. 2012. Directives – Directive 2012/12/EU of The European Parliament and of The Council of 19 April 2012 amending Council Directive 2001/112/EC relating to fruit juices and certain similar products intended for human consumption. 2012 [27/03/2016]. Available from: http://eur-lex.europa.eu/legal-content/EN/TXT/PDF/?uri=CELEX:32012L0012&qid=1459049485105&from=EN
8. Department of Environment Food & Rural Affairs. Producing and distributing food-guidance. Food Standards: labelling, durability and composition. 2015 [27/03/2016]. Available from: https://www.gov.uk/guidance/food-standards-labelling-durability-and-composition#fruit-juices-and-nectars
9. Chauliac M, Hercberg S. Changing the food environment: the French experience. Advances in nutrition. 2012;3(4):605S-10S.
10. Watkins DA, Olson ZD, Verguet S, Nugent RA, Jamison DT. Cardiovascular disease and impoverishment averted due to a salt reduction policy in South Africa: an extended cost-effectiveness analysis. Health policy and planning. 2016;31(1):75-82.
11. Restrepo BJ, Rieger M. Denmark's Policy on Artificial Trans Fat and Cardiovascular Disease. American journal of preventive medicine. 2016;50(1):69-76.
12. Leth T, Jensen HG, Mikkelsen AA, Bysted A. The effect of the regulation on trans fatty acid content in Danish food. Atheroscler Suppl. 2006;7(2):53-6.
13. ?
14. New York City Health. National Salt Reduction Initiative (NSRI) 2017 [13/03/2017]. Available from: https://www1.nyc.gov/site/doh/health/health-topics/national-salt-reduction-initiative.page
15. The Chip Group. The Chip Group: Helping you make better chips 2016 [22/02/2016]. Available from: http://blog.thechipgroup.co.nz/
16. National Agreement to improve Product Composition 2017 [13/03/2017]. Available from: http://www.akkoordverbeteringproductsamenstelling.nl/dsresource?type=pdf&disposition=inline&objectid=rivmp:303682&versionid=&subobjectname=
17. Angell SY, Cobb LK, Curtis CJ, Konty KJ, Silver LD. Change in trans fatty acid content of fast-food purchases associated with New York City's restaurant regulation: a pre-post study. Annals of internal medicine. 2012;157(2):81-6.
18. Curtis CJ, Clapp J, Niederman SA, Ng SW, Angell SY. US Food Industry Progress During the National Salt Reduction Initiative: 2009-2014. Am J Public Health. 2016;106(10):1815-9.
19. Chip Group Approved Oil List: The Chip Group; 2017 [13/03/2017]. Available from: http://www.thechipgroup.co.nz/approved-oils-1/the-list/
20. World Cancer Research Fund. NOURISHING Framework - Nutrition labels: World Cancer Research Fund; 2016 [22/02/2016]. Available from: http://www.wcrf.org/int/policy/nourishing-framework/nutrition-labels
21. US Food and Drug Administration. Proposed changes to the nutrition facts label 2016 [22/02/2016]. Available from: http://www.fda.gov/Food/GuidanceRegulation/GuidanceDocumentsRegulatoryInformation/LabelingNutrition/ucm385663.htm
22. Doell D, Folmer D, Lee H, Honigfort M, Carberry S. Updated estimate of trans fat intake by the US population. Food Additives & Contaminants: Part A. 2012;29(6):861-74.
23. Van Camp D, Hooker NH, Lin CT. Changes in fat contents of US snack foods in response to mandatory trans fat labelling. Public Health Nutr. 2012;15(6):1130-7.

24. Lee JH, Adhikari P, Kim SA, Yoon T, Kim IH, Lee KT. Trans Fatty Acids Content and Fatty Acid Profiles in the Selected Food Products from Korea between 2005 and 2008. J Food Sci. 2010;75(7):C647-C52.
25. Australian Government. Australia New Zealand Food Standards Code - Standard 1.2.7 - Nutrition, Health and Related Claims - F2013L00054 2013 [22/02/2016]. Available from: https://www.comlaw.gov.au/Details/F2013L00054
26. Food Standard Australia New Zealand. Nutrient Profiling Scoring Calculator for Standard 1.2.7 2016 [16/06/2016]. Available from: http://www.foodstandards.gov.au/industry/labelling/pages/nutrientprofilingcalculator/Default.aspx
27. Policy - Regulation HK.03.1.23.11.11.09909 (2011) on the Control of Claims on Processed Food Labeling and Advertisements 2011 [16/06/2016]. Available from: https://extranet.who.int/nutrition/gina/en/node/22946
28. U.S. Food and Drug Administration. Guidance for Industry: A Food Labeling Guide (8. Claims) [16/06/2016]. Available from: http://www.fda.gov/Food/GuidanceRegulation/GuidanceDocumentsRegulatoryInformation/LabelingNutrition/ucm064908.htm
29. Deparment of Health. Guide to creating a front of pack (FoP) nutrition label for pre-packed products sold through retail outlets: Department of Health; 2013 [17/04/2016]. Available from: https://www.gov.uk/government/uploads/system/uploads/attachment_data/file/300886/2902158_FoP_Nutrition_2014.pdf
30. Ministerio de Salud Publica. 2013 [16/06/2016]. Available from: http://www.produccion.gob.ec/wp-content/uploads/2013/11/reglamento-de-etiquetado-de-alimentos-procesados-para-el-consumo-humano.pdf
31. Commonwealth of Australia. Health Star Rating System 2016 [22/02/2016]. Available from: http://healthstarrating.gov.au/internet/healthstarrating/publishing.nsf/content/home
32. Diario Oficial de La Republica de Chile. Decreto 13 – Reglamento Ley 20.606 sobre Composición Nutricional de los Alimentos y su Publicidad 2015 [16/06/2016]. Available from: http://www.dinta.cl/wp-dintacl/wp-content/uploads/Decreto-13_Ley-super8_do-20150626.pdf
33. Scarborough P, Matthews A, Eyles H, Kaur A, Hodgkins C, Raats MM, et al. Reds are more important than greens: how UK supermarket shoppers use the different information on a traffic light nutrition label in a choice experiment. The international journal of behavioral nutrition and physical activity. 2015;12:151.
34. Ni Mhurchu C, Volkova E, Jiang Y, Eyles H, Michie J, Neal B, et al. Effects of interpretive nutrition labels on consumer food purchases: the Starlight randomized controlled trial. Am J Clin Nutr. 2017;105(3):695-704.
35. Office of the Federal Register. Food Labeling: Nutrition Labeling of Standard Menu Items in Restaurants and Similar Retail Food Establishments 2013 [16/06/2016]. Available from: https://www.federalregister.gov/regulations/0910-AG57/food-labeling-nutrition-labeling-of-standard-menu-items-in-restaurants-and-similar-retail-food-estab
36. Department of Health and Mental Hygiene. Food Preparation and Food Establishments (Article 81, Section 49) - Sodium Warning [16/06/2016]. Available from: http://rules.cityofnewyork.us/content/food-preparation-and-food-establishments-article-81-section-49-sodium-warning-0
37. VanEpps EM, Roberto CA, Park S, Economos CD, Bleich SN. Restaurant Menu Labeling Policy: Review of Evidence and Controversies. Current obesity reports. 2016;5(1):72-80.
38. Cantor J, Torres A, Abrams C, Elbel B. Five Years Later: Awareness Of New York City's Calorie Labels Declined, With No Changes In Calories Purchased. Health affairs. 2015;34(11):1893-900.
39. Bleich SN, Wolfson JA, Jarlenski MP, Block JP. Restaurants With Calories Displayed On Menus Had Lower Calorie Counts Compared To Restaurants Without Such Labels. Health affairs. 2015;34(11):1877-84.
40. Menu labeling regulations and calories purchased at chain restaurants. American Journal of Preventive Medicine. 2013;44(6):595-604.
41. World Cancer Research Fund. NOURISHING Framework - Restrict food marketing 2016 [22/02/2016]. Available from: http://www.wcrf.org/int/policy/nourishing-framework/restrict-food-marketing
42. Office de la protection du consommateur. Advertising targeted at children under 13 years of age. Guide to the Application of Sections 248 and 249 Consumer Protection Act 2013 [16/06/2016]. Available from: https://www.opc.gouv.qc.ca/fileadmin/media/documents/consommateur/sujet/publicite-pratique-illegale/EN_Guide_publicite_moins_de_13_ans_vf.pdf
43. Kent MP, Dubois L, Wanless A. Food marketing on children's television in two different policy environments. International journal of pediatric obesity : IJPO : an official journal of the International Association for the Study of Obesity. 2011;6(2-2):e433-41.

44. Chile Bans ‘Kinder Suprise’ and ‘Happy Meals’ to Combat Child Obesity. The Argentina Independent. 2016 30/05/2016. Available from: http://www.argentinaindependent.com/currentaffairs/latest-news/newsfromlatinamerica/chile-bans-kinder-suprise-and-happy-meals-to-combat-child-obesity/
45. Ministry of Food and Drug Safety. The Special Act on the Safety Management of Children's Dietary Life 2017 [13/03/2017]. Available from: https://www.mfds.go.kr/eng/index.do?nMenuCode=66
46. Bugge AB. Food advertising towards children and young people in Norway. Appetite. 2016;98:12-8.
47. Kent MP, Dubois L, Wanless A. A nutritional comparison of foods and beverages marketed to children in two advertising policy environments. Obesity. 2012;20:1829-37.
48. Kim S, Lee Y, Yoon J, Chung SJ, Lee SK, Kim H. Restriction of television food advertising in South Korea: impact on advertising of food companies. Health Promot Int. 2013;28(1):17-25.
49. Kelly B, Hebden L, King L, Xiao Y, Yu Y, He G, et al. Children's exposure to food advertising on free-to-air television: an Asia-Pacific perspective. Health promotion international. 2016;31(1):144-52.
50. Clark Williams C. Toward a Taxonomy of Corporate Reporting Strategies. Journal of Business Communication. 2008;45(3):232-64.
51. Veerman JL, Cobiac LJ. Removing the GST exemption for fresh fruits and vegetables could cost lives. The Medical journal of Australia. 2013;199(8):534-5.
52. World Cancer Research Fund. NOURISHING Framework - Use economic tools 2016 [22/02/2016]. Available from: http://www.wcrf.org/int/policy/nourishing-framework/use-economic-tools
53. Policies to promote healthy eating in Europe: A structured review of policies and their effectiveness. Nutrition Reviews. 2012;70(3):188-200.
54. Colchero MA, Popkin BM, Rivera JA, Ng SW. Beverage purchases from stores in Mexico under the excise tax on sugar sweetened beverages: observational study. BMJ. 2016;352:h6704.
55. Biro A. Did the junk food tax make the Hungarians eat healthier? Food Policy. 2015;54:107-15.
56. Triggle N. Sugar tax: How it will work? : BBC News; 2016 [17/04/2016]. Available from: http://www.bbc.com/news/health-35824071
57. Colchero MA, Rivera J, Popkin B, Ng S. In Mexico, Evidence Of Sustained Consumer Response Two Years After Implementing A Sugar-Sweetened Beverage Tax. Health affairs. 2017;36(3):564-71.
58. Batis C, Rivera JA, Popkin BM, Taillie LS. First-Year Evaluation of Mexico's Tax on Nonessential Energy-Dense Foods: An Observational Study. PLoS Med. 2016;13(7):e1002057.
59. World Cancer Research Fund. NOURISHING Framework - Harness food supply chain 2016 [22/02/2016]. Available from: http://www.wcrf.org/int/policy/nourishing-framework/harness-food-supply-chain
60. McFadden A, Green JM, Williams V, McLeish J, McCormick F, Fox-Rushby J, et al. Can food vouchers improve nutrition and reduce health inequalities in low-income mothers and young children: a multi-method evaluation of the experiences of beneficiaries and practitioners of the Healthy Start programme in England. BMC public health. 2014;14:148.
61. Young CR, Aquilante JL, Solomon S, Colby L, Kawinzi MA, Uy N, et al. Improving fruit and vegetable consumption among low-income customers at farmers markets: Philly Food Bucks, Philadelphia, Pennsylvania, 2011. Preventing chronic disease. 2013;10:E166.
62. World Cancer Research Fund. NOURISHING Framework - Offer healthy foods 2016 [22/02/2016]. Available from: http://www.wcrf.org/int/policy/nourishing-framework/offer-healthy-foods
63. Fundo Nacional de Desinvolvimento da Educacao. Sobre o PNAE 2016 [16/06/2016]. Available from: http://www.fnde.gov.br/programas/alimentacao-escolar
64. Ministry of Education. National Fund for Education Development, Resolution No 26 of 17 June 2013 (Ministério da Educação, Fundo Nacional de Desenvolvimento da Educação, Resolução No 26 de 17 de junho de 2013). Brazil: Ministry of Education; 2016.
65. Hills A, Nathan N, Robinson K, Fox D, Wolfenden L. Improvement in primary school adherence to the NSW Healthy School Canteen Strategy in 2007 and 2010. Health promotion journal of Australia : official journal of Australian Association of Health Promotion Professionals. 2015;26(2):89-92.
66. Spence S, Delve J, Stamp E, Matthews JN, White M, Adamson AJ. The impact of food and nutrient-based standards on primary school children's lunch and total dietary intake: a natural experimental evaluation of government policy in England. PloS one. 2013;8(10):e78298.
67. Spence S, Delve J, Stamp E, Matthews JN, White M, Adamson AJ. Did school food and nutrient-based standards in England impact on 11-12Y olds nutrient intake at lunchtime and in total diet? Repeat cross-sectional study. PloS one. 2014;9(11):e112648.
68. Lederer A, Curtis CJ, Silver LD, Angell SY. Toward a healthier city: nutrition standards for New York City government. American journal of preventive medicine. 2014;46(4):423-8.
69. Healthy Eating Advisory Service. Improving wellbeing through healthy eating. 2017.

70. Miyoshi M, Tsuboyama-Kasaoka N, Nishi N. School-based "Shokuiku" program in Japan: application to nutrition education in Asian countries. Asia Pacific journal of clinical nutrition. 2012;21(1):159-62.
71. Tanaka N, Miyoshi M. School lunch program for health promotion among children in Japan. Asia Pacific journal of clinical nutrition. 2012;21(1):155-8.
72. Ministry of Education Culture Sports Science and Technology. Reference Intake Values for School Lunch. . Ministry of Education, Culture, Sports, Science and Technology.; 2009.
73. Ministry of Education Culture Sports Science and Technology. A Study on the Implementation Status of School Lunch Program 2008. . Ministry of Education, Culture, Sports, Science and Technology. ; 2009.
74. Department of Health. Public Health Responsibility Deal. Pledges [02/04/2014]. Available from: https://responsibilitydeal.dh.gov.uk/pledges/
75. Victoria State Government. Healthy Eating Policy and Catering Guide for Workplaces 2017 [16/03/2017]. Available from: https://www2.health.vic.gov.au/getfile//?sc_itemid=%7BD5628D86-B37D-42F7-8B9A-687F4A3E85FB%7D
76. Bae SG, Kim JY, Kim KY, Park SW, Bae J, Lee WK. Changes in dietary behavior among adolescents and their association with government nutrition policies in Korea, 2005-2009. Journal of preventive medicine and public health = Yebang Uihakhoe chi. 2012;45(1):47-59.
77. Times T. Ban on fast food near schools approved 2016 [16/06/2016]. Available from: http://www.thetimes.co.uk/article/ban-on-fast-food-near-schools-approved-prm823vpp
78. Department of Health. Change4Life Convenience Stores Evaluation Report [27/03/2016]. Available from: https://www.gov.uk/government/uploads/system/uploads/attachment_data/file/215984/dh_120801.pdf
79. Li KY, Cromley EK, Fox AM, Horowitz CR. Evaluation of the placement of mobile fruit and vegetable vendors to alleviate food deserts in New York City. Preventing chronic disease. 2014;11:E158.
80. Lu W, McKyer EL, Dowdy D, Evans A, Ory M, Hoelscher DM, et al. Evaluating the Influence of the Revised Special Supplemental Nutrition Program for Women, Infants, and Children (WIC) Food Allocation Package on Healthy Food Availability, Accessibility, and Affordability in Texas. J Acad Nutr Diet. 2016;116(2):292-301.
81. Cobb LK, Anderson CA, Appel L, Jones-Smith J, Bilal U, Gittelsohn J, et al. Baltimore City Stores Increased The Availability Of Healthy Food After WIC Policy Change. Health affairs. 2015;34(11):1849-57.
82. Otten JJ, Hekler EB, Krukowski RA, Buman MP, Saelens BE, Gardner CD, et al. Food marketing to children through toys: response of restaurants to the first U.S. toy ordinance. Am J Prev Med. 2012;42(1):56-60.
83. Otten JJ, Saelens BE, Kapphahn KI, Hekler EB, Buman MP, Goldstein BA, et al. Impact of San Francisco's toy ordinance on restaurants and children's food purchases, 2011-2012. Preventing chronic disease. 2014;11:E122.
84. Friel S, Hattersley L, Snowdon W, Thow AM, Lobstein T, Sanders D, et al. Monitoring the impacts of trade agreements on food environments. Obesity reviews : an official journal of the International Association for the Study of Obesity. 2013;14 Suppl 1:120-34.
85. Thow AM, Annan R, Mensah L, Chowdhury SN. Development, implementation and outcome of standards to restrict fatty meat in the food supply and prevent NCDs: learning from an innovative trade/food policy in Ghana. BMC public health. 2014;14:249.
86. Kelly PM, Davies A, Greig AJ, Lee KK. Obesity Prevention in a City State: Lessons from New York City during the Bloomberg Administration. Frontiers in public health. 2016;4:60.
87. World Public Health Nutrition Association Update team. Brazilian dietary guidelines. At last! Guidelines based on food and meals! : World Nutrition; 2014. Available from: http://wphna.org/wp-content/uploads/2015/01/WN-2014-05-12-1050-1051-Update-Guia.pdf
88. Kansagra SM, Kennelly MO, Nonas CA, Curtis CJ, Van Wye G, Goodman A, et al. Reducing sugary drink consumption: New York City's approach. American journal of public health. 2015;105(4):e61-4.
89. Pan American Health Organization. Pan American Health Organization Nutrient Profile Model Washington D.C.: Pan American Health Organization; 2016 [17/04/2016]. Available from: http://www.paho.org/hq/index.php?option=com_content&view=article&id=11662%3Apaho-nutrient-profile-model&catid=1370%3Amicronutrients&Itemid=41739&lang=en
90. Ministry of Health Brazil. Health Surveillance Secretariat: Health situation analysis department. Strategic action plan to tackle noncommunicable diseases in Brazil 2011-2022. Brazil: Ministry of Health; 2011.
91. Ministry of Health South Africa. Strategic Plan for the Prevention and Control of Non-Communicable Diseases 2013-17 2013 [22/02/2016]. Available from: http://www.hsrc.ac.za/uploads/pageContent/3893/NCDs%20STRAT%20PLAN%20%20CONTENT%208%20april%20proof.pdf

92. Public Health England. Sugar reduction: From evidence to action. London: Public Health England; 2015.
93. Monteiro CA, Cannon G, Moubarac JC, Martins AP, Martins CA, Garzillo J, et al. Dietary guidelines to nourish humanity and the planet in the twenty-first century. A blueprint from Brazil. Public health nutrition. 2015;18(13):2311-22.
94. Ministry of Health Brazil. Dietary guidelines for the Brazilian population 2014 [22/02/2016]. Available from: http://www.foodpolitics.com/wp-content/uploads/Brazilian-Dietary-Guidelines-2014.pdf
95. World Health Organization Europe. European Food and Nutrition Action Plan 2015–2020. Copenhagen: WHO Europe; 2015.
96. State Services Commission. Best Practice Guidelines for Departments Responsible for Regulatory Processes with Significant Commercial Implications [17/04/2016]. Available from: http://www.ssc.govt.nz/resources/2814/all-pages
97. Australian Government Federal Register of Legislation. National Health and Medical Research Council Act 1992 [cited 16/06/2016]. Available from: https://www.legislation.gov.au/Details/C2006C00354
98. Food Standards Australia New Zealand. Stakeholder Engagement Strategy 2013-2016. . Wellington: FSANZ New Zealand.; 2013.
99. Children's Food Trust. Our research 2016 [22/02/2016]. Available from: http://www.childrensfoodtrust.org.uk/childrens-food-trust/our-research/
100. Centres for Disease Control. National Health and Nutrition Examination Survey 2016 [22/02/2016]. Available from: http://www.cdc.gov/nchs/nhanes.htm
101. Health and Social Care Information Centre. National Child Measurement Programme 2016 [22/02/2016]. Available from: http://www.hscic.gov.uk/ncmp
102. US National Institutes of Health. Time-Sensitive Obesity Policy and Program Evaluation (R01) 2016 [22/02/2016]. Available from: http://grants.nih.gov/grants/guide/pa-files/PAR-12-257.html
103. CONSEA. Building up the National Policy and System for Food and Nutrition Security: the Brazilian experience [08/03/2016]. Available from: https://www.fao.org.br/download/Seguranca_Alimentar_Ingles.pdf
104. Government VS. What is Healthy Together Victoria 2017 [16/03/2017]. Available from: https://www2.health.vic.gov.au/about/publications/policiesandguidelines/What%20is%20Healthy%20Together%20Victoria
105. Strugnell C, Millar L, Churchill A, Jacka F, Bell C, Malakellis M, et al. Healthy together Victoria and childhood obesity-a methodology for measuring changes in childhood obesity in response to a community-based, whole of system cluster randomized control trial. Archives of public health = Archives belges de sante publique. 2016;74(Electronic-eCollection):16.
106. Ministry of Health New Zealand. Healthy Families NZ 2016 [17/04/2016]. Available from: http://www.health.govt.nz/our-work/preventative-health-wellness/healthy-families-nz
107. Lock K, Gabrijelcic-Blenkus M, Martuzzi M, Otorepec P, Wallace P, Dora C, et al. Health impact assessment of agriculture and food policies: lessons learnt from the Republic of Slovenia. Bull World Health Organ. 2003;81(6):391-8.
108. Government of South Australia. Health in All Policies: The South Australian Approach 2016 [22/02/2016]. Available from: http://www.sahealth.sa.gov.au/wps/wcm/connect/public+content/sa+health+internet/health+reform/health+in+all+policies
109. Health in all policies as a priority in Finnish health policy: a case study on national health policy development. Scandinavian journal of public health. 2013;41(11 Suppl):3-28.
110. Delany T, Lawless A, Baum F, Popay J, Jones L, McDermott D, et al. Health in All Policies in South Australia: what has supported early implementation? Health Promot International. 2016;31(4):888-98.
